# A longitudinal footprint of genetic epilepsies using automated electronic medical record interpretation

**DOI:** 10.1038/s41436-020-0923-1

**Published:** 2020-08-10

**Authors:** Shiva Ganesan, Peter D. Galer, Katherine L. Helbig, Sarah E. McKeown, Margaret O’Brien, Alexander K. Gonzalez, Alex S. Felmeister, Pouya Khankhanian, Colin A. Ellis, Ingo Helbig

**Affiliations:** 1 Division of Neurology, Children’s Hospital of Philadelphia, Philadelphia, PA USA; 2 The Epilepsy NeuroGenetics Initiative (ENGIN), Children’s Hospital of Philadelphia, Philadelphia, PA USA; 3 Department of Biomedical and Health Informatics (DBHi), Children’s Hospital of Philadelphia, Philadelphia, PA USA; 4 Department of Neurology, University of Pennsylvania, Perelman School of Medicine, Philadelphia, PA USA

**Keywords:** electronic medical records, Human Phenotype Ontology, childhood epilepsy, neurogenetics

## Abstract

**Purpose:**

Childhood epilepsies have a strong genetic contribution, but the disease trajectory for many genetic etiologies remains unknown. Electronic medical record (EMR) data potentially allow for the analysis of longitudinal clinical information but this has not yet been explored.

**Methods:**

We analyzed provider-entered neurological diagnoses made at 62,104 patient encounters from 658 individuals with known or presumed genetic epilepsies. To harmonize clinical terminology, we mapped clinical descriptors to Human Phenotype Ontology (HPO) terms and inferred higher-level phenotypic concepts. We then binned the resulting 286,085 HPO terms to 100 3-month time intervals and assessed gene–phenotype associations at each interval.

**Results:**

We analyzed a median follow-up of 6.9 years per patient and a cumulative 3251 patient years. Correcting for multiple testing, we identified significant associations between “Status epilepticus” with *SCN1A* at 1.0 years, “Severe intellectual disability” with *PURA* at 9.75 years, and “Infantile spasms” and “Epileptic spasms” with *STXBP1* at 0.5 years. The identified associations reflect known clinical features of these conditions, and manual chart review excluded provider bias.

**Conclusion:**

Some aspects of the longitudinal disease histories can be reconstructed through EMR data and reveal significant gene–phenotype associations, even within closely related conditions. Gene-specific EMR footprints may enable outcome studies and clinical decision support.

## INTRODUCTION

Genetic factors are increasingly implicated in childhood epilepsies, and with the advent of massive parallel sequencing technologies more than 200 novel genetic etiologies have been identified in the last decade.^1–3^ Identification of an underlying genetic etiology is particularly relevant in the developmental and epileptic encephalopathies (DEE), which represent the severe end of the spectrum of the childhood epilepsies.^4–6^ Causative genetic etiologies can be identified in up to 30% of individuals with DEE without explanatory structural lesions or metabolic findings.^7–11^ The genetic architecture of the childhood epilepsies is characterized by prominent heterogeneity; even the most common genetic etiologies including *SCN1A*, *SCN2A*, or *STXBP1* only account for 1% or less of the patient population.^9,12,13^ In contrast to massive parallel sequencing studies that are performed on tens of thousands of individuals, understanding phenotypic data at this scale remains a major obstacle. The disease course in childhood epilepsies is dynamic over time,^14,15^ and longitudinal information on natural history and outcome is limited due to the rarity of each genetic cause. Furthermore, clinical characterization of rare genetic entities is often restricted to case series,^16–19^ which cannot distinguish clinical features associated with a specific gene from clinical features shared between related diseases.

The adoption of electronic medical records (EMR) provides a new opportunity to leverage clinical data for genomic research. Large national and international initiatives have started to link biorepositories with EMR data,^20,21^ and several phenotyping algorithms are already validated to extract clinical features.^22–24^ However, the longitudinal aspect of EMR data that is relevant to assess disease histories over time largely has been unexplored. Maintaining the temporal relationship between clinical features is critical in disorders that follow prominent age-related patterns, such as the childhood epilepsies. More importantly, the overall quality of EMR data is unexplored, and clinical data entry into EMR systems is often considered a nuisance by providers. Therefore, accuracy and precision of clinical phenotypes in a system primary created for billing purposes may rightfully be questioned. Nevertheless, given the magnitude and availability of EMR data, even limited reliability would allow for conclusions about longitudinal disease histories that would otherwise require time-consuming manual chart review.

Here, we mapped EMR data in individuals with childhood epilepsies who underwent genetic testing to Human Phenotype Ontology (HPO) terms. We analyzed data of 658 individuals followed for a median 6.9 years with a cumulative 3251 patient years, including 232 individuals with a definite genetic diagnosis. We assessed and identified significant gene–phenotype associations, demonstrating that EMR data can be used to identify gene-specific signatures even in clinically closely related disease entities such as the childhood epilepsies.

## MATERIALS AND METHODS

### Ethics statement

Informed consent for participation in this study was obtained from subjects themselves or parents of all probands in agreement with the Declaration of Helsinki, and the study was completed per protocol with local approval by the Children’s Hospital of Philadelphia (CHOP) Institutional Review Board (IRB 15-12226).

### Patient recruitment

The current analysis was performed on individuals enrolled in the Epilepsy Genetics Research Project (EGRP) at Children’s Hospital of Philadelphia, which has enrolled patients with known or presumed genetic epilepsies since 2014. Genetic etiologies in the EGRP cohort were assessed in a clinical and research setting, including gene panel sequencing (*n* = 100), exome sequencing (*n* = 109), or other testing modalities including single-nucleotide polymorphism (SNP) arrays (*n* = 9) or single-gene tests (*n* = 14). Genetic results were reviewed and, if necessary, reclassified according to the criteria of the American College of Medical Genetics and Genomics (ACMG).^25^

### Electronic medical record data extraction

During the time period of this study, all patients were followed within the CHOP Care Network, including the main hospital inpatient and outpatient unit and 50 satellite clinics. Encounters outside this network could not be captured through the medical records and were unavailable for this study. All providers within the CHOP network use a single unified EMR system (EPIC, Verona, WI) that can be accessed via the Clarity database (EPIC). Every provider contact is documented within the EMR, including clinic and emergency room visits, hospital admissions, telephone calls, refills, and visits for laboratory work and imaging. All documented contacts are referred to as “encounters” within the EMR. At every encounter, the medical personnel is responsible for updating a current list of all active medical diagnoses, which is termed the “problem list.” Additionally, at a subset of encounters including inpatient and outpatient visits, providers are required to assign “encounter diagnoses,” which are the medical problems associated with or addressed in that encounter. Problem lists and encounter diagnoses within the Clarity database are encoded in Intelligent Medical Objects terms (IMO, Northbrook, IL) that are mapped to International Classification of Diseases, Ninth/Tenth Revision (ICD9/10) codes. In contrast to ICD9/10 codes, IMO provides an intuitive language interface that includes common clinical terminology such as “Absence seizure” or “Generalized epilepsy” rather than ICD codes. For our study, we extracted encounter diagnoses and problem lists for all individuals enrolled in the study for every encounter documented in the EMR. All information included in our study was derived from routine clinical care; encounters for research purposes only were not included. From each encounter, we extracted the problem lists, encounter diagnoses, and age of the patient at the encounter. We included IMO problems and diagnosis terms based on a selection of ICD10 codes related to neurological diagnoses (F00-F99, G00-G99, P90, Q00-Q07, R25-R29, R40-R49, R56, R62, R90, and R94.01) and merged IMO terms on the diagnosis and problem list.

### Construction of a dictionary for mapping to HPO terms

For the cumulative list of diagnoses and problems, we created a dictionary that mapped IMO terms to terms in the HPO.^26^ This custom dictionary was created by a team of providers and researchers who reviewed each of the 1479 IMO terms associated with neurology-related ICD9/ICD10 codes mentioned above. We used the Clinical Text Analysis and Knowledge Extraction System (cTAKES) natural language processing algorithm^27^ to generate a preliminary dictionary, which was subsequently reviewed and adapted manually. To avoid false annotations, we limited the annotation of epilepsy syndromes to high-level phenotypic terms (Supplementary Data, Table S2).

### Inferring higher-level clinical concepts through parental terms in the HPO tree (propagation)

**Fig. 1:**
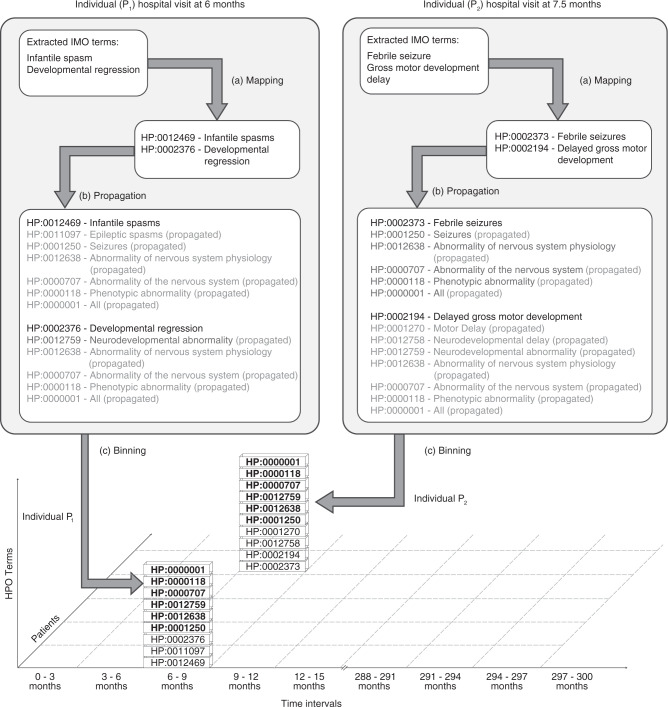
Mapping, propagation, and binning as a three-step process for clinical data harmonization from electronic medical records. We present the hypothetical example of two individuals seen for an outpatient encounter or admission at 6 months or 7.5 months respectively. By (**a**) mapping clinical diagnosis terms to Human Phenotype Ontology (HPO) terms, the clinical data are harmonized to a joint ontological framework. By adding all higher-level (ancestral) terms, it is now possible to identify common shared higher-level terms, a method we refer to as (**b**) propagation. Finally, by (**c**) binning the propagated unique phenotype terms (removing duplicated terms) into discrete time intervals (3 months), phenotypes can be compared across both individuals. Shared HPO terms (e.g., “Seizures,” HP:0001250) are highlighted in bold. *IMO* Intelligent Medical Objects terminology.

In contrast to clinical terms, the structure of the HPO assigns each clinical concept a place in its ontological tree. This allows for the identification of higher-level terms, which may be common in two individuals if two lower-level terms are distinct (Fig. 1). For example, “Focal seizures” (HP:0007359) and “Generalized seizures” (HP:0002197) both have “Seizures” (HP:0001250) as a common parent term. Identifying and assigning parental, higher-level terms therefore enables the identification of shared phenotypic features. In addition to the assigned HPO term derived from the merged diagnosis and problem lists, we added all higher-level terms for each encounter, a method that we refer to as propagation (Fig. 1). Consistent with the general use in the literature, we use parental terms to refer to immediate superterms, e.g., “Seizures” (HP:0001250) is a parent term for “Generalized seizures” (HP:0002197), which is in turn a parent of “Absence seizures” (HP:0002121). We use ancestors and ancestral terms to refer to higher-level terms more generally, e.g., both “Seizures” (HP:0002197) and “Generalized seizures” (HP:0002197) are ancestors of “Absence seizures” (HP:0002121).

### Mapping of HPO data to time intervals

For the final analysis, the age of the patient at each encounter was placed into 3-month time bins ranging from age 0 to 25 years, including a total of 100 bins. For example, the first time bin includes all encounters between birth and 3 months of age. For each time bin, all assigned and propagated HPO terms per individual were merged and duplicates per individual removed, duplicates referring to situations where the individual had multiple encounters within the time bin or had multiple IMO terms that mapped onto the same HPO term. This resulted in a set of HPO terms per individuals per each time bin, including all higher-level ancestral terms (Fig. 1).

### Assessment of EMR usage

Every individual had a unique time span during which treatment was provided within the care network. We defined “EMR usage” as the time period between the minimum age and maximum age at documented patient encounters. This definition of EMR usage is largely operational to define time intervals where information on a given individual was definitely unavailable and where a given individual did not contribute to the overall analysis, i.e., outside of the EMR usage window. For our study, we assumed that EMR usage was uninterrupted between the minimum and maximum age of encounters. However, this assumption does not imply that all medical information was fully documented within the period of EMR usage.

Each HPO term for every individual at any of the 100 time points was coded as “present” if the time point was within the window of an individual’s EMR usage. A term was coded as “absent” if the time point was within an individual’s EMR usage window, but the term was not coded for this individual. Finally, the term was coded as “not applicable” if the time point was outside the EMR usage window for an individual.

### Genotype–phenotype associations

Frequencies for each HPO term were determined using the number of individuals with available data at each time point, including both the initially assigned HPO terms and the propagated higher-level terms. For each causative genetic etiology in the cohort, frequencies for each HPO term at each time point were assessed and compared with the frequency of each HPO term in the remainder of the cohort. The significance of the association was determined using a two-sided Fisher’s exact test. Subsequently, for each gene–phenotype combination the 3-month interval with the most significant association was identified (p_gxp_max_). For example, for the association of *SCN1A* and “Status epilepticus” (HP:0002133), the most significant association was at 1 year with −log_10_(p_gxp_max_) = 6.74. Each phenotype at each time point was analyzed independently and no information was used from past or future time points. Correction for multiple testing was subsequently performed using the Benjamini–Hochberg method with a false discovery rate (FDR) of 0.05. All statistical tests were performed using the R Statistical Framework, including the ggplot2 package.

## RESULTS

### Electronic medical record data captures longitudinal features

**Fig. 2:**
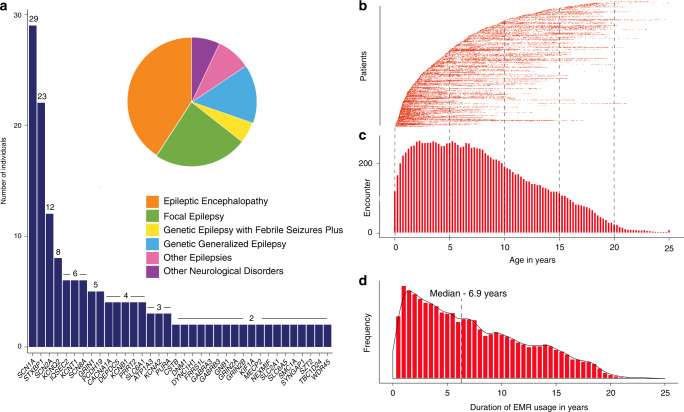
Electronic medical record (EMR) data in genetic epilepsies can be mapped to discrete time intervals. (**a**) Genetic etiologies in 658 individuals with known or presumed genetic epilepsies and distribution of epilepsy syndromes are shown (insert). (**b**) Individual data points in the electronic medical records used for the current analysis (*n* = 62,104). *X*-axis displays age at the given data point, individual patients are stacked (*y*-axis) and sorted by age at earliest encounter. (**c**) Binning of EMR data into 3-month intervals between birth and age 25 (100 bins). Only a subset of individuals are informative at each 3-month interval with a peak between 2 and 7 years of age and a maximum of 266/658 contributing to a given time interval at 2.25 years. Individuals not contributing to EMR usage at a given time point have not yet had contact with the health network, have left the network, or have not yet reached the given age. (**d**) Duration of EMR usage in the overall cohort is shown, defined as the time interval between the first and last encounter captured in the EMR, collectively adding up to 3251 years.

We analyzed data from 658 individuals with a wide range of epilepsy syndromes and genetic etiologies, including 336 male and 322 female individuals. Epileptic encephalopathies (*n* = 268) were the most common genetic etiologies, followed by focal epilepsies (*n* = 156) and genetic generalized epilepsies (*n* = 97). In our cohort, 102 distinct genetic etiologies were identified, including 36 causative genes identified in two or more individuals (Fig. 2a, Table S3). The most common genetic etiologies in our cohort included *SCN1A* (*n* = 29), *STXBP1* (*n* = 22), *SCN2A* (*n* = 12), *KCNQ2* (*n* = 8), and *KCNT1* (*n* = 6). The median age of seizure onset was 1.34 years (range 0 to 18 years). We restricted the analysis to neurology-related diagnoses and problems coded by clinicians during patient care encounters, comprising 62,104 data points (Fig. 2b). For each subject, we defined the window of EMR usage based on the age at first and last patient encounters, the ages at which the individual’s disease course was captured in the EMR. We then binned EMR usage and neurology-related diagnoses and problems into 3-month intervals (Fig. 2c). The number of individuals contributing to each of the 100 time points ranged from 5 to 266 with a median of 142 individuals per time point. EMR usage in the cohort was highest between age 2 and age 7. The median duration of EMR usage was 6.9 years (range 0–25) with a cumulative EMR usage of 3251 patient years (Fig. 2d).

### Diagnoses and active medical problems at 62,104 patient encounters are mapped to 286,085 HPO terms across 3-month time intervals

**Fig. 3:**
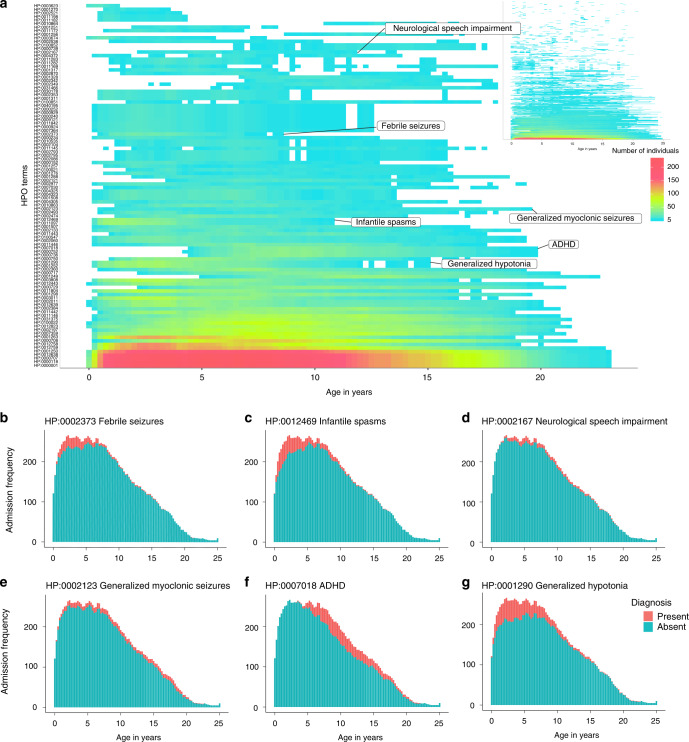
Clinical features have characteristic time-dependent distributions. Diagnosis and problem lists in 658 individuals are mapped to 528 distinct clinical concepts that show unique longitudinal distributions in the patient cohort. (**a**) Phenotypic features are distributed across time in the cohort. *X*-axis denotes age, *y*-axis displays phenotypic features sorted by frequency of each Human Phenotype Ontology (HPO) term, color indicates number of individuals with a certain HPO term at each time point. The 100 most common HPO terms are shown; the inset shows all the HPO terms. The frequency of various HPO terms reflects the longitudinal trajectory of these features in neurodevelopmental disorders, including (**b**) “Febrile seizures” (HP:0002373), (**c**) “Infantile spasms” (HP:0012469), (**d**) “Neurological speech impairment” (HP:0002167), (**e**) “Myoclonic seizures” (HP:0002123), (**f**) “Attention deficit–hyperactivity disorder” (ADHD, HP:0007018), and (**g**) “Generalized hypotonia” (HP:0001290).

For each of the 62,104 patient encounters, we extracted the clinical terms assigned as a diagnosis or active medical problem from the EMR, representing the diagnosis and active problems that appear in the official patient letter generated from this patient encounter. We then mapped the 1479 unique neurologic diagnoses and problems coded by providers to 350 discrete terms in the HPO after binning into 100 discrete 3-month intervals. The HPO represents a controlled dictionary with defined semantic relationships.^26^ For every individual and at each time interval, we also added all higher-level parental (ancestral) terms in the HPO, generating a total of 528 discrete HPO terms. In total, the mapping to defined HPO terms, inclusion of higher-level terms, and binning to 100 discrete time intervals resulted in 286,085 HPO terms.^24,28^ This mapping allowed us to identify common higher-level terms between individuals at each time point and to determine the true frequency of each phenotypic term in the cohort, as more specific terms resulted in an inclusion of all higher-level phenotypic terms, even though these higher-level terms may not have been directly mapped (Fig. 3a, Supplementary Data).

### Clinical features in genetic epilepsies have characteristic time-dependent distributions

We next assessed the distribution of each phenotypic feature over time to determine whether known phenotypic features are represented correctly in the EMR. We found that the distribution of phenotypic features reflects the known age-dependent distribution of many clinical diagnoses associated with neurodevelopmental disorders. For example, febrile seizures (HP:0002373) typically occur between 6 months and 6 years and did, in fact, map to the corresponding time intervals (Fig. 3b).^29^ Likewise, infantile spasms (HP:0012469) represent a distinct seizure type that manifests in infancy but may continue throughout early childhood in genetic epilepsies, which is reflected in the mapping of this term based on EMR data (Fig. 3c).^30^ Further examples include neurological speech impairment (HP:0002167; Fig. 3d),^31^ generalized myoclonic seizures (HP:0002123; Fig. 3e),^32^ attention deficit–hyperactivity disorder (ADHD, HP:0007018; Fig. 3f),^33^ and generalized hypotonia (HP:0001290; Fig. 3g).^34,35^ The observed time-dependent distribution of the above clinical features and other phenotypic features (Supplementary Data) suggests that within the wider cohort, our mapping and EMR diagnoses capture the age-dependent distribution of these phenotypes correctly, even though individual terms may have been inadequately assigned by the treatment providers.

### EMR data allows for the identification of time-dependent gene–phenotype associations

**Fig. 4:**
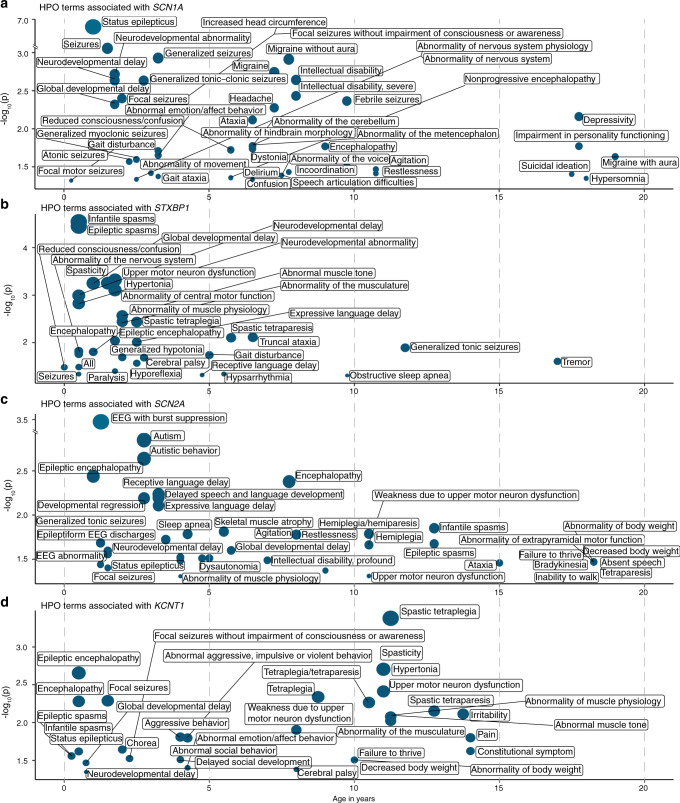
Genetic etiologies demonstrate time-dependent phenotypic associations. Phenotypic features associate with discrete genetic etiologies at specific time points when binned into 3-month intervals. Clinical terms associated with individual genes occur at different time intervals. The Human Phenotype Ontology (HPO) terms associated with (**a**) *SCN1A*, (**b**) *STXBP1*, (**c**) *SCN2A*, and (**d**) *KCNQ2* are shown as an example with only the time interval with the most significant association for each HPO term shown. *X*-axis denotes patient age, *y*-axis denotes −log_10_ of the *p* value (Fisher’s exact test). *EEG* electroencephalogram.

We next analyzed the association of the 528 HPO terms with the 36 genetic etiologies identified in two or more individuals included in our study (Table S4). When limiting the analysis to the most significant time interval for each gene–phenotype association and excluding HPO modifier terms that specify age of onset, severity, or specific quality of phenotypic features, 859 nominally significant associations were identified (Table S5). The nominally significant associations were used to reconstruct longitudinal phenotype maps for each genetic etiology (Fig. 4 and Supplementary Data). For the global analysis of gene–phenotype associations, we corrected for multiple testing using the Benjamini–Hochberg procedure with an FDR of 0.05. Four associations were significant after multiple testing, including “Status epilepticus” (HP:0002133; *p* = 1.84e−7) with *SCN1A* at 1.0 years, “Severe intellectual disability” (HP:0010864; *p* = 2.96e−6) with *PURA* at 9.75 years, and “Infantile spasms” (HP:0012469; *p* = 2.85e−5) and “Epileptic spasms” (HP:0011097; *p* = 3.54e−5) with *STXBP1* at 0.5 years. These gene–phenotype associations replicate the known natural history for *SCN1A*,^36^ *PURA*,^37^ and *STXBP1*,^16^ and demonstrate that our EMR data mapping and harmonization approach correctly identifies known gene–phenotype associations in our cohort that were previously reported. We manually reviewed patient charts for *SCN1A* at 1.0 years, *PURA* at 9.75 years, and *STXBP1* at 0.5 years and found that that phenotypic terms were accurate and not biased by individual providers.

### Factors driving gene–phenotype associations can be identified from EMR data

**Fig. 5:**
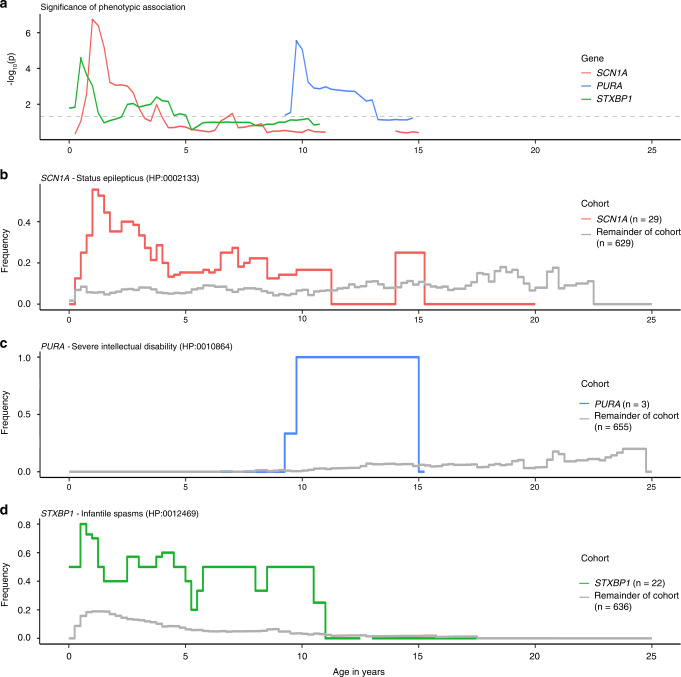
Phenotypic associations are time-dependent. Associations between genetic etiologies and specific phenotypic terms differ over time, including the association with *SCN1A* and “Status epilepticus” (HP:0002133, red, **a**, **b**), *PURA* and “Severe Intellectual Disability” (HP:0010864, blue, **a**, **c**), and *STXBP1* and “Infantile spasms” (HP:0012469, green, **a**, **d**). (**a**) Distribution of the strength of the association over time. (**b**–**d**) Frequency of the specific HPO terms in patients with and without the genetic etiology over time that give rise to the observed associations.

We next examined how significant gene–phenotype associations emerged from our cohort. As our primary analysis only included the most significant association across time for each gene–phenotype combination, we expanded the analysis for three gene–phenotype associations across all time intervals (Fig. 5a). We included the association of “Status epilepticus” (HP:0002133) with *SCN1A*, “Severe intellectual disability” (HP:0010864) with *PURA*, and “Infantile spasms” (HP:0012469) with *STXBP1*. We excluded the association of “Epileptic spasms” (HP:0011097) with *STXBP1* as this phenotypic term is the direct parent term of “Infantile spasms” (HP:0012469) and therefore added no additional information. The association of the significance over time demonstrates that the association peak for all three associations occurs at discrete time points. To better understand features leading to the observed patterns, we examined the frequencies of the specific phenotypes in individuals with and without the genetic etiology (Fig. 5b–d).

We found two patterns. For “Status epilepticus” (HP:0002133) with *SCN1A* and “Infantile spasms” (HP:0012469) with *STXBP1*, the phenotypic features show a high frequency in the gene-positive group for *SCN1A* at 1.0 years and *STXBP1* at 0.5 years. The *PURA* signal is only based on three individuals between 10 and 15 years, all individuals assigned the phenotypic term “Severe intellectual disability” (HP:0010864). The frequency of this phenotypic term increased in the overall cohort over time, but only at the age of 9.75 years is this discrepancy large enough to generate the most extreme *p* value (*p* = 2.96e−6). The phenotypic associations with *PURA* further highlight that we are only able to make assertions about phenotypic associations within the range of EMR usage. EMR usage for all three *PURA*-related disorders only overlapped between 4.75 years and 10 years, indicating that important phenotypic associations outside this age window were largely inaccessible to us and were likely missed.

## DISCUSSION

In our study, we assessed whether data derived from EMR might aid in identifying longitudinal phenotypic patterns for genetic epilepsies. Within the limitations of this study, we demonstrate that heterogeneous EMR can be harmonized and mapped through a common framework such as the HPO. Using this tool, we discovered time-dependent associations between genetic etiologies and phenotypes that recapitulate essential aspects of the natural history of these conditions. Our study may therefore provide a general framework to reconstruct age-dependent phenotypes in genetic epilepsies and neurodevelopmental disorders from EMR data. While this approach can only capture a subset of the phenotypic depth, EMR data are ubiquitously available, and such a framework can assist in supplementing growing genetic data sets with longitudinal phenotypes.

The age dependence of clinical features in our study demonstrates two important properties of longitudinal phenotypes. First, even though our study included 658 individuals, no more than 266 individuals contribute to each time point (Fig. 2b, c). We expect that similar limitations will apply to many studies performed in pediatric settings and emphasize the importance of controlling for the time window when individuals received care within the health-care system. In our study, we refer to this window of health-care utilization as EMR usage. Second, clinical features have characteristic trajectories in our cohort (Fig. 3), reflecting the time-dependent nature of clinical characteristics such as febrile seizures (1–5 years), infantile spasms (6 months–5 years), and ADHD (5 years and older). The distinct patterns of 528 phenotypic features generate a complex pattern over time that drives associations between features and specific genetic etiologies. The time-dependent associations for specific phenotypes potentially can be used as a quality control mechanism to assess the validity of additional data sets given concerns that true associations in EMR data sets may be contaminated by templated notes and copy-forward mechanisms.

Given the rarity of individual genetic epilepsy syndromes, knowledge about the natural history of these disorders is typically acquired through case series. While this method is well suited to delineate the phenotypic range of specific genetic etiologies, comparisons between disorders are challenging. We reasoned that capturing phenotypic features longitudinally across a large patient cohort would allow significantly associated clinical features to emerge. Applying this framework, we identified that “Infantile spasms” (HP:0012469), present in 12% of our cohort, only shows a significant association with *STXBP1*. In addition, this most significant association was limited to a relative narrow time interval around 6–9 months. Our framework therefore allows us to identify significant gene–phenotype associations in conditions with a broad phenotypic range, using real-world data derived from an ongoing collection of clinical data captured in the EMR. We acknowledge that many other genetic etiologies included in our study are known to be associated with infantile spasms. However, these etiologies were too rare in our cohort or the age when infantile spasms typically emerge was outside the period of EMR usage for individuals with other genetic etiologies. While our study was primarily focused on identifying phenotype association with single genes, we observed that further associations can be captured when genes are grouped (Supplementary Data).

The data extraction and mapping algorithms applied in our study have several limitations, including the restriction to diagnosis and problem lists related to neurology-related ICD9/10 codes and our inability to assess negated phenotypes.^38,39^ Despite these conceptual limitations, our study provides a general model that outlines the three critical components of any framework to capture longitudinal phenotypic data: data extraction, mapping/harmonization techniques, and strategies for temporal binning. Addressing these limitations can be conceptualized as improving one of these components within this framework.

There are additional notable limitations of an automated EMR extraction approach compared with a traditional retrospective chart review. These limitations must be weighed against the potential benefit of the automated extraction approach, which allows for significantly larger sample sizes due to the prohibitive cost of manual chart review. For example, the encounter diagnoses and problem lists are entered by various health-care providers, which may introduce bias, including a potential lack of detail in the coded features. This limitation becomes particularly relevant when trying to assess the absence of significant associations. We therefore do not claim that our approach generates a detailed representation of the overall phenotypic landscape but identifies features that emerge despite the inherent limitations of EMR data.

A further limitation is our focus on a single health-care network. While this allowed us to include homogeneous data and perform manual chart review, we cannot claim that our methods are immediately generalizable to other data sets of combined genomic and EMR data. However, our study presents a first attempt to show that EMR data can reconstruct some aspects of the disease history in genetic epilepsies. This may be further tested in future EMR/exome data sets once these resources become available.

To harmonize EMR data, we had to commit to several arbitrary decisions that may have affected our results and could be modified and improved in future studies. For example, we assumed that care during the window of EMR usage is uninterrupted within our health-care system. In principle, we cannot exclude that a subset of individuals received care at other institutions within this period that was not documented. However, both our manual chart review and clinical experience suggested that this assumption was correct for the majority of individuals and that the proportion of individuals with interrupted or parallel outside care was relatively small. A further deliberate decision was the choice of bin width, which we set at 3 months based on our assumption of the relevant time frame in which changes in neurological phenotypes would manifest. We explored the effect of bin width (Supplementary Data) and found that adjusting bin width may result in better detection of some phenotypic associations, as is the case of stronger associations of neonatal seizure phenotypes with *KCNQ2* when decreasing bin width (Supplementary Data).

We believe that the methodology developed in our study is widely generalizable to multicenter data, where information on complex phenotypic histories can be provided in a de-identified format. In parallel to collaborative data sets in genomic studies, this would allow for joint analysis of large cohorts to provide information about the natural history of rare disorders, supplementing the role of manual phenotyping in such studies. Likewise, we expect that our method can further be refined by adding more granular phenotypic data, such as phenotypic terms derived from full-text patient notes or standardized data elements within the EMR. As our method is built to identify associations with rare genetic conditions at specific time intervals, our tools can be validated in a guided manner, allowing for a review of a small subset of patient charts only at specific time points.

Finally, we chose HPO version 1.2 (release format version: 1.2; data version: releases/2017–12–12; downloaded on 10 March 2018) for our analysis, which was the most recent version at the time of initial data analysis. This HPO version does not yet fully reflect the latest seizure classifications of the International League Against Epilepsy (ILAE).^4^ Efforts to update the HPO are currently underway within the epilepsy community. Such improvements could easily be integrated into future iterations of our general framework.

In summary, our study demonstrates that EMR data can be used to elucidate aspects of the longitudinal disease histories in epilepsies and neurodevelopmental disorders. This is accomplished in our study through harmonization of clinical terminology through the HPO framework and binning into discrete time intervals. Using this method, we show that several genetic etiologies including *SCN1A*-, *STXBP1*-, and *PURA*-related disorders have time-dependent associations with distinct clinical features that stand out from the wider group of known or presumed genetic epilepsies. Identifying disease trajectories using large-scale phenotypic data may become a critical component for clinical decision support and learning health-care systems, particularly in rare genetic neurological disorders where available clinical information is limited.

## Supplementary information

Supplementary Data

Supplementary Table 2

Supplementary Table 3

Supplementary Table 4

Supplementary Table 5

## Data Availability

All computer code is made available at https://github.com/shiva-g/The-Cube.
