# Supplementary material for: A longitudinal footprint of genetic epilepsies using automated electronic medical record interpretation
Source: Genet Med. 2020 Aug 10;22(12):2060–70. doi: 10.1038/s41436-020-0923-1 (PMC7708303; doi:10.1038/s41436-020-0923-1)
Supplement: Supplementary file 1 — Supplementary Data [file 41436_2020_923_MOESM1_ESM.pdf]

A longitudinal footprint of genetic epilepsies using automated electronic medical record interpretation

Shiva Ganesan, MS, Peter D. Galer, MSc, Katherine L. Helbig, MS, Sarah E. McKeown, MS, Margaret O'Brien, BS, Alexander K. Gonzalez, MS, MBA, Alex S. Felmeister, PhD, Pouya Khankhanian, MD, Colin A. Ellis, MD, and Ingo Helbig, MD

Supplementary Data

Supplementary Data 1
Study cohort 2
Window of EMR usage 2
Rationale for HPO dictionary 3
Delineating syndromes versus symptoms within the HPO dictionary 4
Effect of bin width on phenotypic associations 5
Effect of increasing bin width to 6 months, 12 months, and 24 months 5
Effect of decreasing bin width to 1 week, 1 month, and 6 weeks 7
Predictive power of gene-phenotype associations in *SCN1A* 8
Effect of gene grouping 10
Additional supplementary figures as cited in main manuscript 17
Supplementary References 19

Study cohort

Using the EGRP protocol, patients were consented for use of bio samples, existing genetic data, and clinical data including data derived from the Electronic Medical Records (EMR) in a research context. Informed consent for participation in this study was obtained from subjects themselves or parents of all probands in agreement with the Declaration of Helsinki, and the study was completed per protocol with local approval by the local institutional review board (IRB). For research enrollment, individuals and families were approached in an inpatient setting (neurology floor, epilepsy monitoring unit, pediatric intensive care unit, and neonatal intensive care unit) and outpatient setting (epilepsy clinic and neurogenetics/epilepsy genetics clinic). For the current study, we also included 49 individuals without epilepsy, mainly when individuals had genetic etiologies typically associated with seizures, such as *STXBP1* or *SCN2A,* and were seen in the neurogenetics clinic of the Children's Hospital of Philadelphia.

Window of EMR usage

To assess phenotypic data, the current study used electronic medical record (EMR) data derived from the EPIC (1979 Milky Way, Verona, WI 53593) database at Children's Hospital of Philadelphia, extracting encounter diagnoses and problem lists for all individuals included in the study. In order to capture the entire duration of medical care that individuals received within the CHOP Network, we also extracted all medication prescriptions and refills. At any given time point, the proportion of individuals with EMR usage without assigned phenotype terms reflects individuals followed within the healthcare network for non-neurological issues prior to developing neurological problems, for example routine pediatric care. The proportion of individuals with EMR use without assigned neurology-related phenotype terms was 0.82 (537/658 individuals) at birth and decreased over time.

Rationale for HPO dictionary

As the HPO is an ontology of phenotypic findings, we mapped Intelligent Medical Objects (IMO) terms related to diseases and epilepsy syndromes (e.g. “Dravet Syndrome” and “Juvenile Myoclonic Epilepsy”) to high-level terms in the HPO (e.g. “Abnormality of central nervous system”) to avoid false positive annotation. For example, patients with “Dravet Syndrome” frequently have myoclonic seizures (HP:0002123), but a significant subset of individuals do not have this seizures type at the time point that the syndromal diagnosis is coded in the EMR. Accordingly, the HPO term for myoclonic seizures was not inferred from the epilepsy syndrome diagnosis but was only added if listed separately on the merged problem and diagnosis list.

A method that we refer to as “propagation” was utilized during the assignment of HPO terms to participants. In addition to HPO terms that were assigned to a participant from the merged diagnosis and problem list, all higher-level HPO terms were added for a particular time point for each individual. For example, if Individual 1 was assigned the term “Focal impaired awareness seizure” (HP:0002384), this individual would also be assigned the following higher-level terms: “Focal-onset seizure” (HP:0007359), “Seizures” (HP:0001250), “Abnormality of nervous system physiology” (HP:0012638), “Abnormality of the nervous system” (HP:0000707), “Phenotypic abnormality” (HP:0000118), and “All” (HP:0000001). Propagation was performed to capture phenotypic similarity between individuals if related, but not identical, HPO terms are assigned. In the example above, Individual 1 with the assigned term “Focal-onset seizure” (HP:0007359) and Individual 2 with the assigned term “Absence seizure” (HP:0002121) would both have “Seizures” (HP:0001250) and all higher-level terms assigned as shared ancestral terms, capturing the Most Informative Common Ancestor (MICA) terms for both individuals [1].

The HPO version used for this study was HPO version 1.2 (release format-version: 1.2; data-version: releases/2017-12-12; downloaded on 3/10/18).

Delineating syndromes versus symptoms within the HPO dictionary

Given that problem and diagnosis lists in the EMR only capture some degree of the complete phenotypic information, we expected a substantial amount of clinical information related to patient similarity to be excluded by our framework. Some of this omission was expected, as we purposefully either ignored syndrome-based terms or mapped them to high-level concepts in the ontology. For example, we mapped terms such as "*PURA*-related neurodevelopmental disorder" [2, 3] to "CNS abnormality" (HP:0000707), a relatively high-level and uninformative term. This mapping was based on our decision to maintain a strict separation between "symptoms" and "syndromes" to avoid diagnostic terms associated with epilepsy syndromes that artificially inflate the association with the underlying genetic etiologies even though specific features were not observed in all individuals.

In our study, delineating syndromes versus symptoms was particularly relevant in *SCN1A*-related epilepsies, where patients were frequently labelled with "Dravet Syndrome" rather than using descriptions of the clinical symptoms. To remain constant in our approach, we only coded "Dravet Syndrome" to the higher-level term "CNS abnormality" (HP:0000707). Accordingly, even though the clinical history of Dravet Syndrome, with fever-related episodes of status epilepticus or hemiclonic seizures, is one of the most significant gene-phenotype associations in the genetic epilepsies [4, 5], the first significant association with specific HPO terms occurred only at 1.0 years. From a clinical standpoint, patients typically have frequent prolonged fever-related seizures in the first year of life that initially lead to the genetic diagnosis. This discrepancy suggests that our current methods to map phenotypic data from EMR is imperfect and incompletely represents some aspects of the natural history. We expect that with improvements in technologies to map clinical data, some of these issues will be overcome.

Effect of bin width on phenotypic associations

For our current study, we chose an arbitrary bin width of 3 months assuming that this bin width represents an adequate trade-off between the overall number of comparisons and sufficient granularity to assess changes in phenotypes. While our main analysis is based on a 3-months bin width, we subsequently explored the effect of widening and shortening the bin width on the phenotypic associations observed in our study. In summary, we find that the majority of gene-phenotype associations remain unchanged. However, some additional associations may emerge that could be beneficial during specific age groups.

Effect of increasing bin width to 6 months, 12 months, and 24 months

We expanded the bin width and assessed the gene-phenotype associations at all time intervals compared to the 3-months binning (**Figure S1**). We observed that while majority gene-phenotype associations at each time point remain correlated, the overall correlation between 3 month and the selected bin decrease with larger bin width.

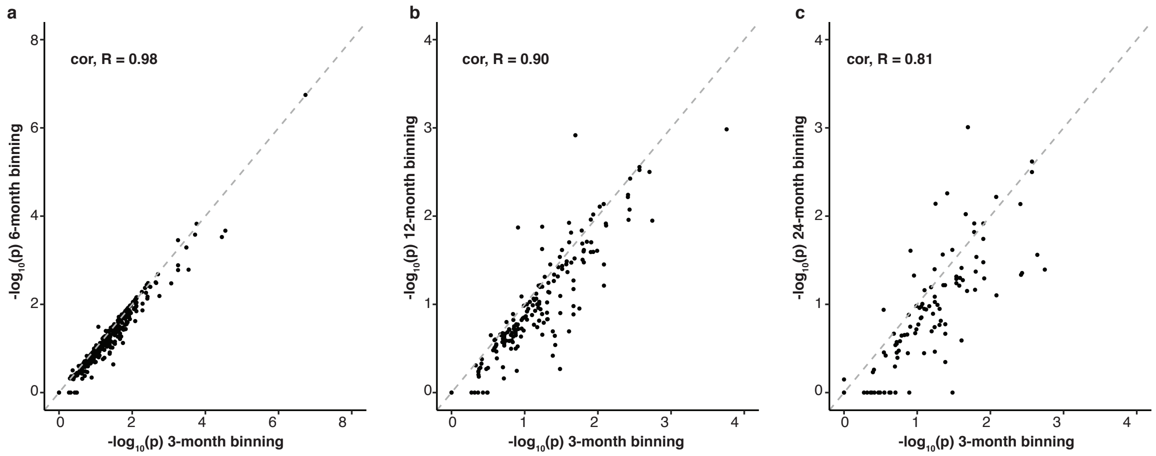

Figure S1. Gene-phenotype associations comparing the binning for 3 months versus 6 months (**a**), 12 months (**b**), and 18 months (**c**). Values for gene-phenotype associations are plotted as -$\log_{10}$(p-values) comparing values for 3 months (y-axis) and the other time intervals on the x-axis. With increasing bin width, the correlation between the gene-phenotype associations at 3 month and the selected bin width become less prominent. However, the majority of gene-phenotype associations remain correlated.

We then examined gene-phenotype associations where the 3-months association deviated from other time intervals most prominently (**Figure S2**). When comparing the 3-months and 24-months binning, we observed that the association of *TBC1D24* and symptomatic seizures (HP:0011145) from birth to three months represented one of the most prominent outliers and was no longer significant with the 24-months binning. The reason for this drop in association was the higher frequency of this HPO term in the overall cohort at birth to three months, which resulted in this reduction of significance.

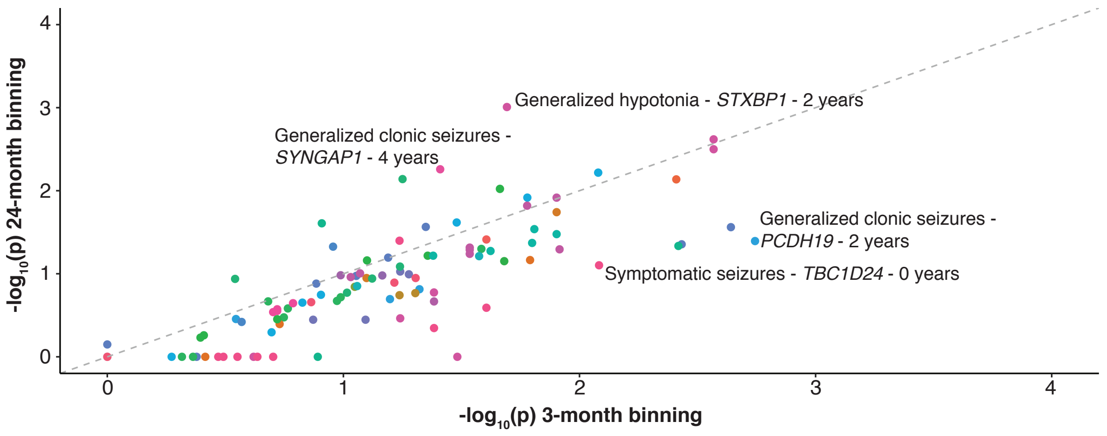

Figure S2. Gene-phenotype associations comparing the binning for 3 months versus 24 months, highlighting outliers that indicate gene-phenotype associations that are different between both binning sizes. Dots in the upper left reflect associations that become stronger with the 24 months bin size, the lower right corner captures gene-phenotype associations that are more prominent in the 3-months bin but decrease in significance with the 24-months bin. Dots represent $-\log_{10}$(p-values) of gene-phenotype associations.

No single gene-phenotype association at either time prominently emerged as a new association, but some existing associations became stronger. The association of generalized hypotonia (HP:0001290) with *STXBP1* became more prominent as the larger binning was able to capture clinical data from individuals who had EMR usage at non-overlapping time intervals, e.g. patients who were followed at our institution at non-overlapping ages. Capturing individuals with non-overlapping EMR usage window represents one advantage of adjusting the binning window, especially for individuals presenting beyond infancy.

Effect of decreasing bin width to 1 week, 1 month, and 6 weeks

We subsequently decreased the bin width to 6 weeks, 1 month, and 1 week (**Figure S3**). Overall, we observed that none of the associations at 3 months became more significant as indicated by the lack of points in the lower right quadrant in **Figure S4**. However, a number of gene-phenotype associations became more significant, most prominently the association of *KCNQ2* with seizures in the first time interval (birth to one month). This increased association of seizures with *KCNQ2* follows the natural history of *KCNQ2*-related seizures that typically occur in two distinct phenotypic groups, benign neonatal seizures (OMIM121200) and *KCNQ2* encephalopathy (OMIM613720). Both disease groups start with seizures approximately at the same age (5 days), but seizures only persist in the subset of individuals with *KCNQ2* encephalopathy. It is therefore reasonable to expect that smaller binning around the time when all individuals have seizures capture the strong association, as is seen in *KCNQ2*.

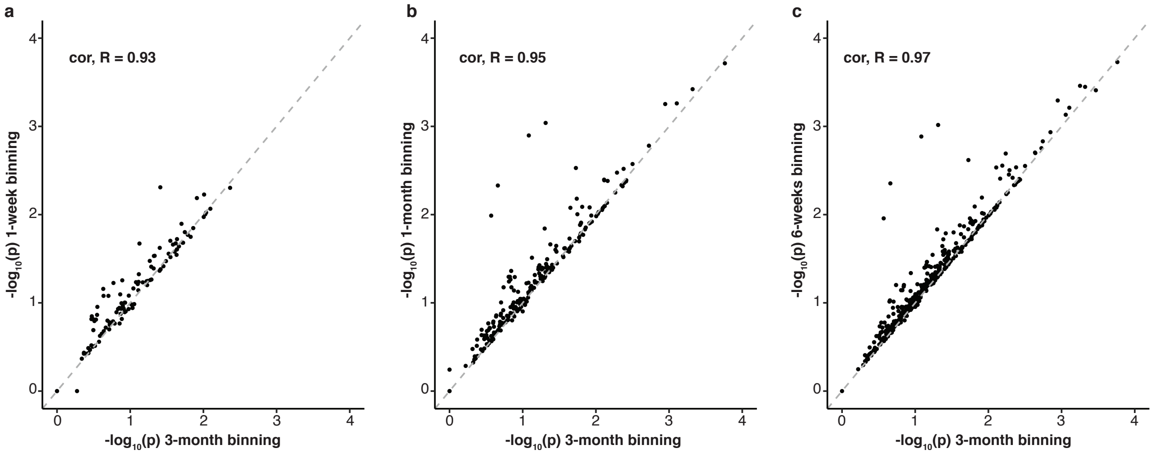

Figure S3. Gene-phenotype associations comparing the binning for 3 months versus 1 week (**a**), 1 month (**b**), and 6 weeks (**c**). Values for gene-phenotype associations are plotted as -$\log_{10}$(p-values) comparing values for 3 months (x-axis) and the other time intervals on the y-axis. With increasing bin width, the correlation between the gene-phenotype associations at 3-month and the selected bin width become less prominent. However, the majority of gene-phenotype associations remain correlated.

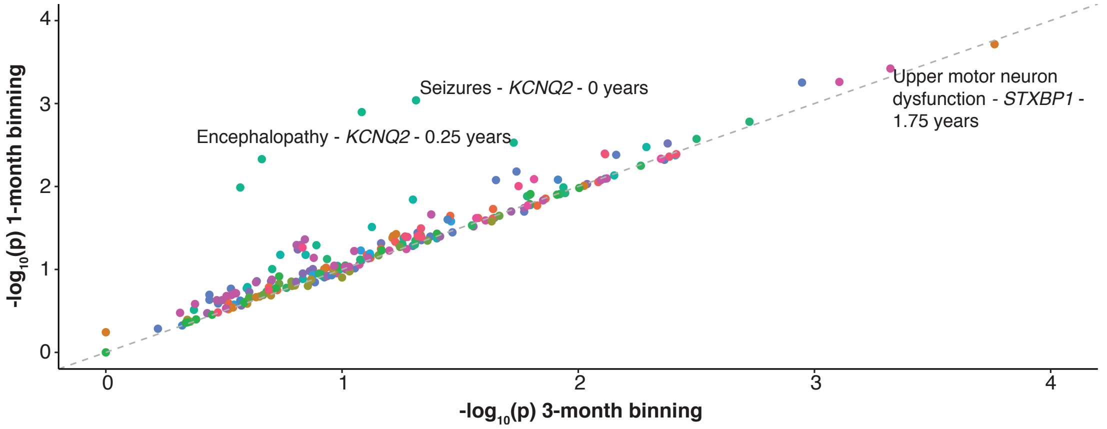

Figure S4. Gene-phenotype associations comparing the binning for 3 months versus 1 month, highlighting outliers that indicate gene-phenotype associations that differ between both binning sizes. Dots in the lower left reflect associations that become stronger with the 3-months bin size; the upper left captures gene-phenotype associations that are more prominent in the 1-months bin but decrease in significance with the 24-months bin. Dots represent -$\log_{10}$(p-values) of gene-phenotype associations. The most prominent change is seen with the association of seizures and *KCNQ2*, which increases in significance with smaller bins.

Predictive power of gene-phenotype associations in *SCN1A*

In order to assess whether gene-phenotype association may be predictive of future phenotypic features, we assessed the association between phenotypes at a given time interval (t_1) and the subsequent time interval (t_2). A high odds ratio (OR) for phenotype HP_1 at t_1 and phenotype HP_2 at t_2 suggests that that HP_1 is predictive of HP_2 at the subsequent time interval. We compared the OR for HP_1 at t_1 and phenotype HP_2 at t_2 in individuals with disease causing variants in *SCN1A* to the remainder of the cohort, assessing all pair-wise combination of HPO terms at all 100 time intervals. We then assessed the t_1-HP_1/t_2-HP_2 combinations where the 95% CI intervals for the OR did not overlap. Of the 260,100 comparisons, only 11 comparisons had non-overlapping ORs for the *SCN1A* cohort and the remainder of the cohort. Of these comparisons 5/11 had non-informative associations between parent and child terms (e.g. “Neurodevelopmental abnormality”; HP:0012759 and “Global developmental delay”; HP:0001263) or modifier terms, **the remaining 6 associations indicated scenarios where HPO terms had a high** OR in the remaining cohort but a low OR in the *SCN1A* cohort (**Table S1**) between 1.75 and 2.75 years.

t_1 (years)	t_2 (years)	HPO term at t_1	HPO term at t_2	OR *SCN1A* (95% CI)	OR cohort (95% CI)
1.75	2	Focal-onset seizure HP:0007359	Neurodevelopmental abnormality HP:0012759	**0** (0-1.45)	**3.71** (1.60-9.42)
1.75	2	Focal-onset seizure HP:0007359	Neurodevelopmental delay HP:0012758	**0** (0-1.45)	**3.96** (1.71-10.04)
2.25	2.5	Focal-onset seizure HP:0007359	Neurodevelopmental abnormality HP:0012759	**0.08** (0.00-1.34)	**3.88** (1.70-9.72)
2.25	2.5	Focal-onset seizure HP:0007359	Neurodevelopmental delay HP:0012758	**0.08** (0-1.34)	**4.04** (1.77-10.12)
2.5	2.75	Neurodevelopmental delay HP:0012758	Focal-onset seizure HP:0007359	**0** (0-0.92)	**2.64** (1.28-5.69)
2.5	2.75	Neurodevelopmental abnormality HP:0012759	Focal-onset seizure HP:0007359	**0** (0-0.92)	**2.86** (1.37-6.29)

Table S1. Comparison of HPO term associations between neighboring bins, assessing the association between HPO terms at bin 1 (t_1) and bin 2 (t_2) for combinations of HPO terms, comparing the odds ratios (OR) in the *SCN1A* cohort and the remaining cohort. The table shows the associations where the 95% confidence intervals do not overlap.

The observed associations can be interpreted as follows: Between the age of 1.75 and 2.75 years, there is a strong association between focal seizures and developmental delay in the overall cohort where presence of one phenotype is predictive for the presence of the other phenotype in the next time interval, i.e. three months later. The odds ratios in the wider cohorts for these associations are between 2.5 and 4, suggesting a 2-4 times increased risk of having one phenotype three months later if the other phenotype had been assigned at a given time point. However, this association is not true for individuals with *SCN1A*-related epilepsies, who have a significantly lower risk for either having focal seizures followed by developmental delay or developmental delay followed by focal seizures.

One explanation for this observation is the fact that focal seizures are commonly seen in genetic epilepsies that also have developmental delay as a presenting feature. However, individuals with *SCN1A*-related epilepsies tend to have more generalized seizures and focal seizures when they possess a disease-causing variant in *SCN1A* without prominent developmental features.

It is worth noting that only a very small subset of phenotype associations were captured using this analysis and that *SCN1A* was the most common genetic etiology in our cohort (n=29). Given the relatively modest findings in *SCN1A*, we did not pursue a similar analysis in the other genetic etiologies that were less common.

Effect of gene grouping

Given the identified gene-phenotype association, we explored whether novel associations emerge when genes are grouped. We grouped genes in the OMIM Early Infantile Epileptic Encephalopathy (EIEE) phenotypic series (230 genes, 29 genes in the cohort with at least one individual, a total of 125 individuals), voltage gated ion channels as group by Gene Ontology (GO) term GO:0005216 (688 genes, 24 genes in 96 individuals), and all genetic etiologies seen in our cohort (102 genes, 232 individuals). For prominent gene-phenotype associations, we then compared the frequency in cases and controls across the entire age span.

In brief, we made the following observations when combining genes into groups. When grouping OMIM EIEE genes, we observed stronger associations between genetic etiologies and phenotypes (**Figure S5**) along three major phenotypes, including seizures (HP:0001250), global developmental delay (HP:0001263), and spastic tetraplegia (HP:0002510). These three phenotypic associations occurred in distinct time intervals with seizures in the first three years of life, developmental delay between the ages of 2.5 years and 7 years, and spastic tetraplegia between 7.5 and 15 years. While not all phenotype associations are true for each genetic etiology (for example, individuals with *SCN1A*-related epilepsies typically do not have spastic tetraplegia), this gene grouping delineates three distinct age-related phenotype associations with known OMIM EIEE genes (**Figure S6**).

We observed a related pattern when assessing the effect of grouping ion channel genes, which overlap significantly with the OMIM EIEE genes (**Figures S7**, **S8**), including an association with encephalopathy (HP:0001298).

When comparing individuals with an explanatory genetic etiology in our cohort (102 distinct genetic etiologies in a total of 232 individuals), we observed a strong association with global developmental delay (HP:0001263) in the first years of life (**Figures S9**, **S10**), indicating that the presence of any causative genetic etiology increases the risk of developmental issues in our cohort by a factor of two (74% vs. 32% being the highest frequency in both groups).

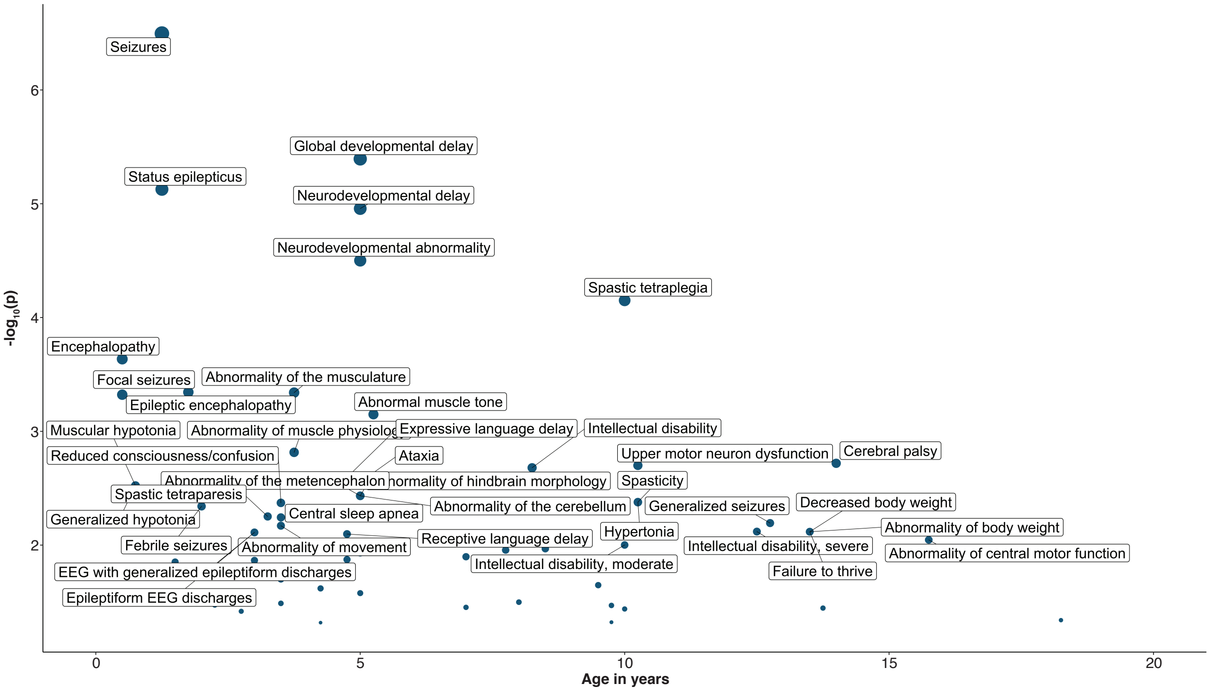

Figure S5. The HPO terms associated with 29 genetic etiologies out of the 230 Early Infantile Epileptic Encephalopathy (EIEE) genes as listed in the OMIM phenotypic series are shown with only the time interval with the most significant association for each HPO term displayed. X-axis denotes patient age, y-axis denotes $-\log_{10}$ of the p-value (Fisher's exact test). HPO term annotations for the most significant associations with $-\log_{10}$(p-value) greater than 2 are shown.

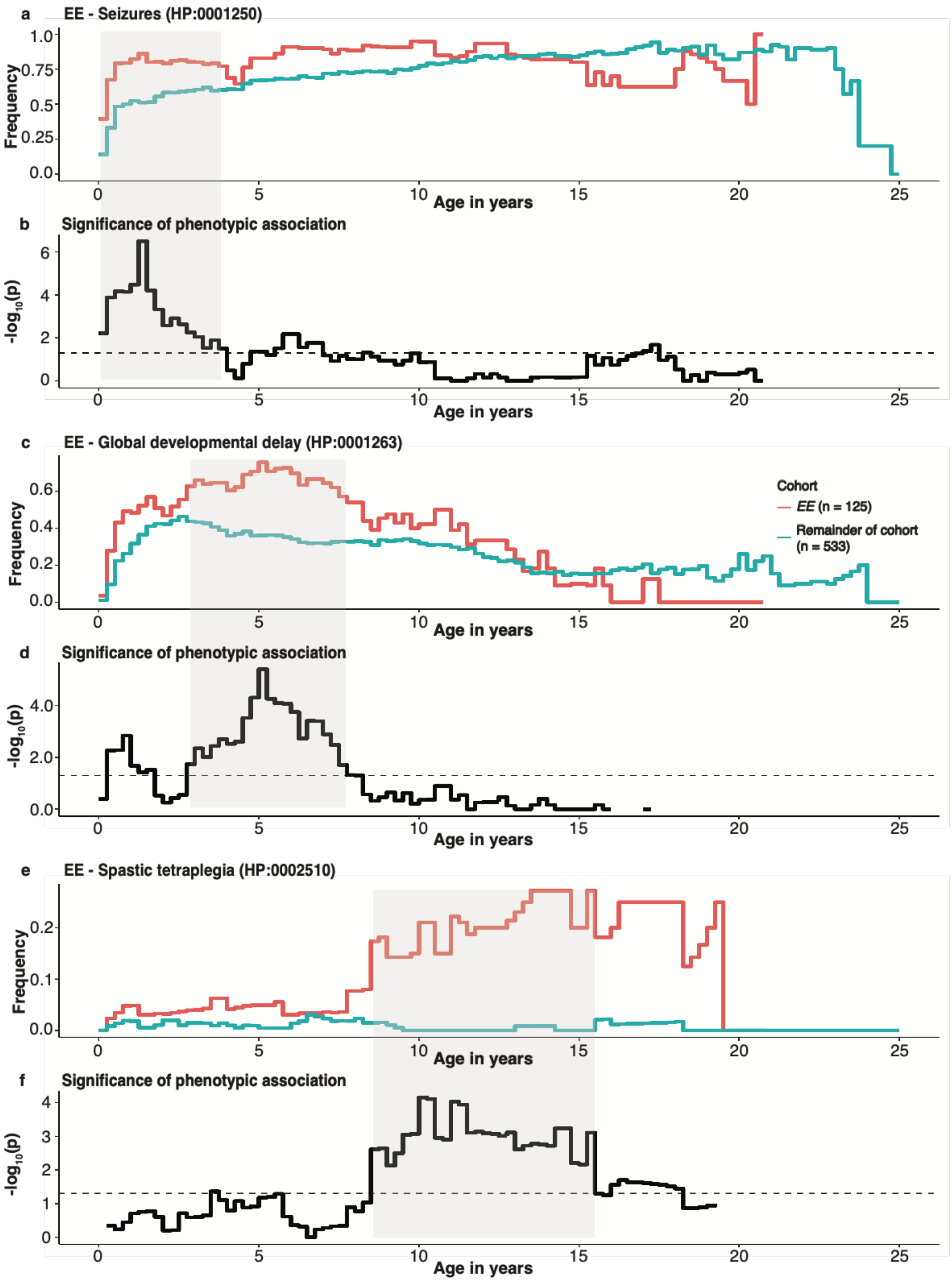

Figure S6. Associations between OMIM EIEE genes and specific phenotypic terms differ over time, including "Seizures" (HP:0001250), "Global developmental delay" (HP:0001263), and "Spastic tetraplegia" (HP:0002510). Grey bars indicate age ranges when phenotypic associations are significant.

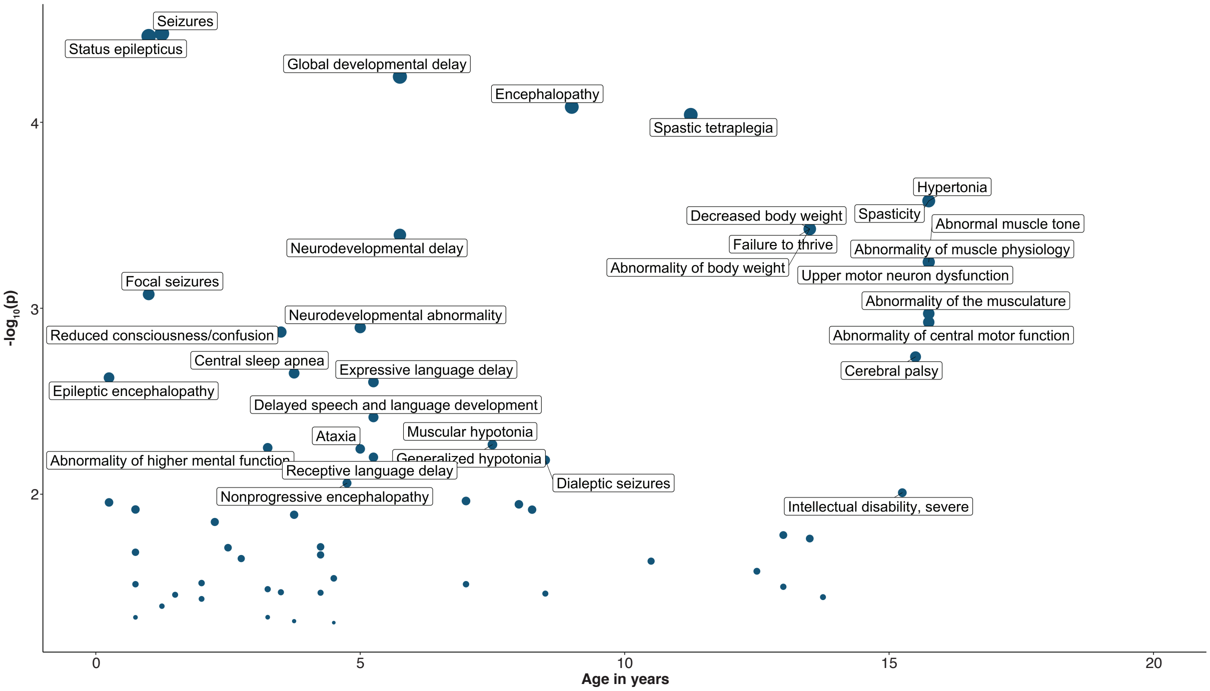

Figure S7. The HPO terms associated with 24 genes out of 96 genetic etiologies with a GO term of "ion channel activity" are shown with only the time interval with the most significant association for each HPO term displayed. X-axis denotes patient age, y-axis denotes -$\log_{10}$ of the p-value (Fisher's exact test). HPO term annotations for the most significant associations with -$\log_{10}$(p-value) greater than 2 are shown.

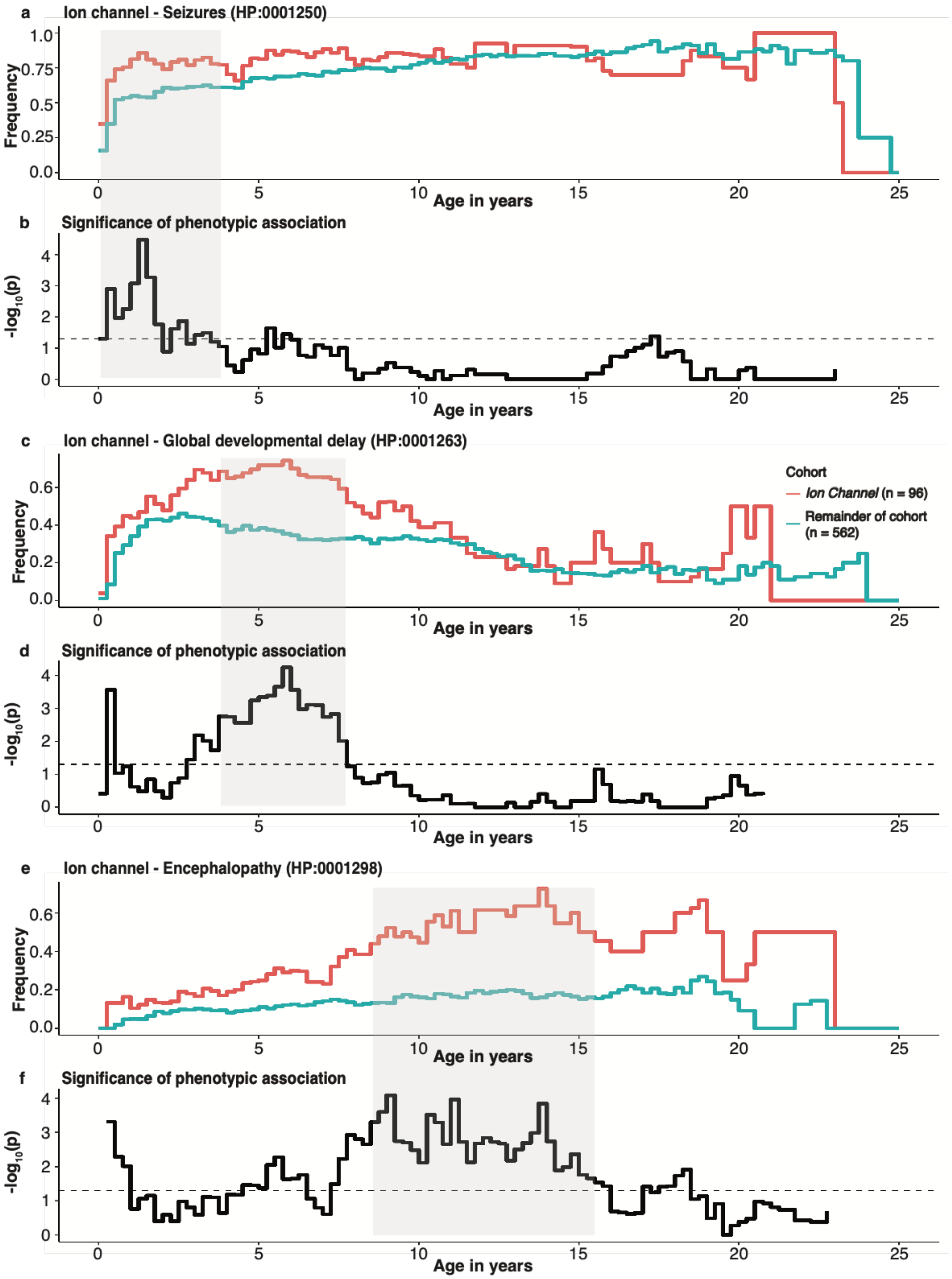

Figure S8. Associations between genetic etiologies with a GO term of "ion channel activity" (GO:0005216) and specific phenotypic terms differ over time, including "Seizures" (HP:0001250), "Global developmental delay" (HP:0001263), and "Spastic tetraplegia" (HP:0002510). Grey bars indicate age ranges when phenotypic associations are significant.

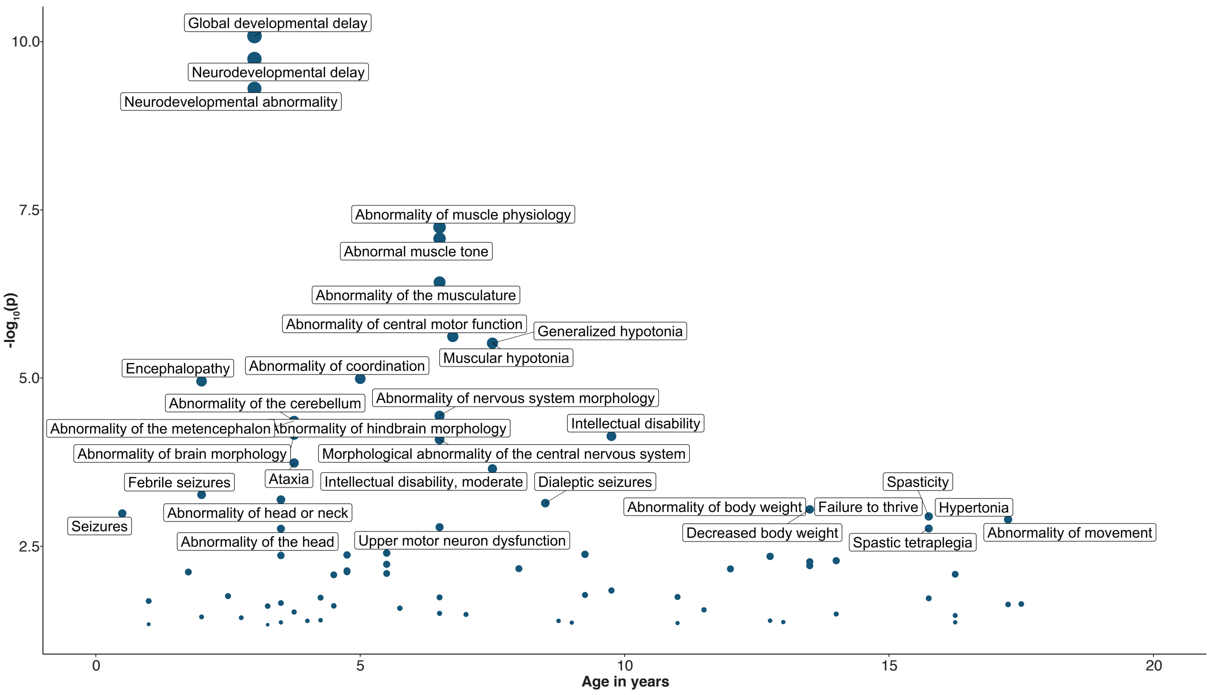

Figure S9. The HPO terms associated with all genetic etiologies in our cohort (102 distinct genetic etiologies in a total of 232 individuals) are shown with only the time interval with the most significant association for each HPO term displayed. X-axis denotes patient age, y-axis denotes -$\log_{10}$ of the p-value (Fisher's exact test). HPO term annotations for the most significant associations with -$\log_{10}$(p-value) greater than 2.5 are shown.

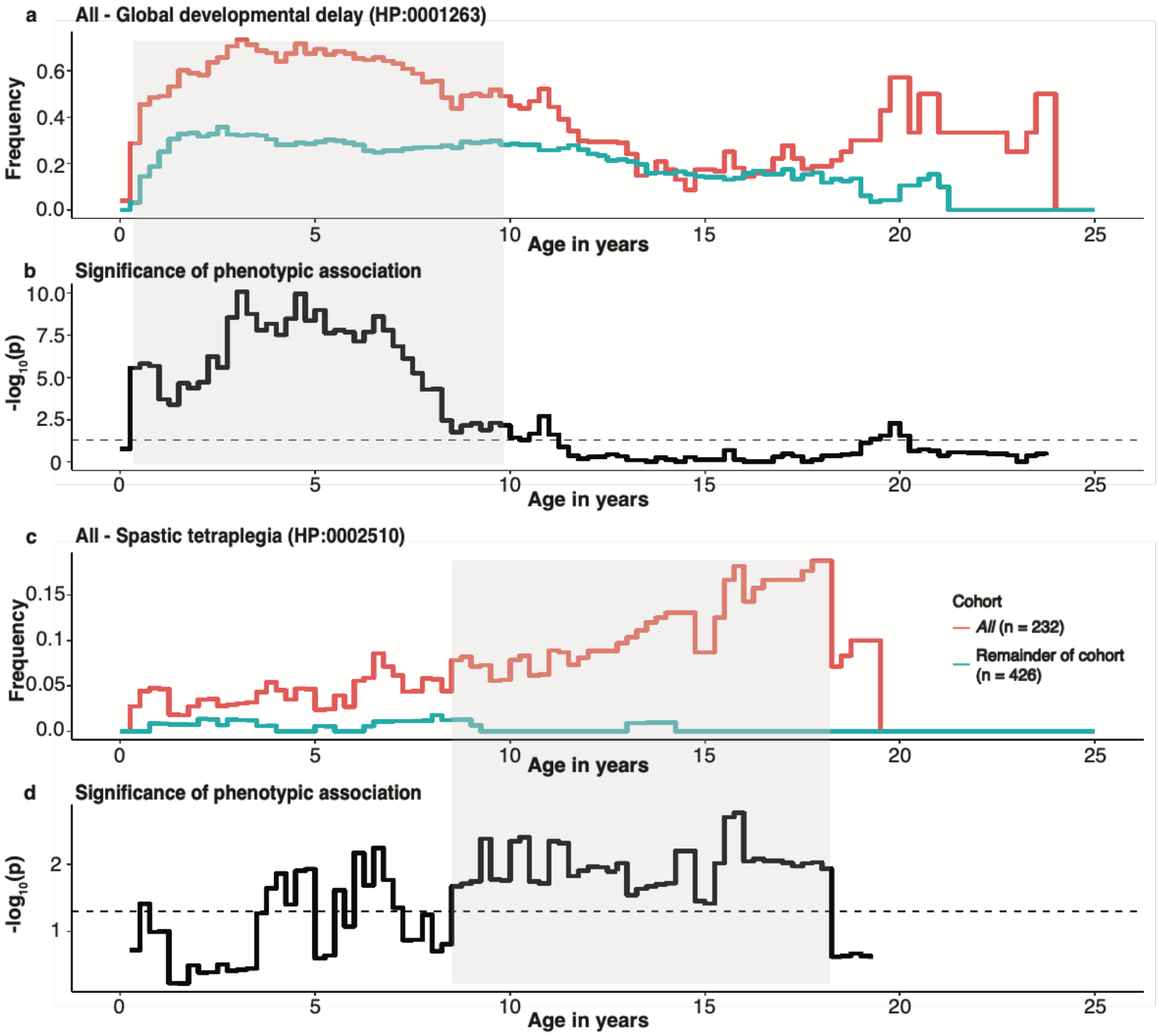

Figure S10. Associations between all genetic etiologies (232 individuals, 102 genetic etiologies) and "Global developmental delay" (HP:0001263) and "Spastic tetraplegia (HP:0002510). Grey bar indicates age range when the phenotypic association is significant.

Additional supplementary figures as cited in main manuscript

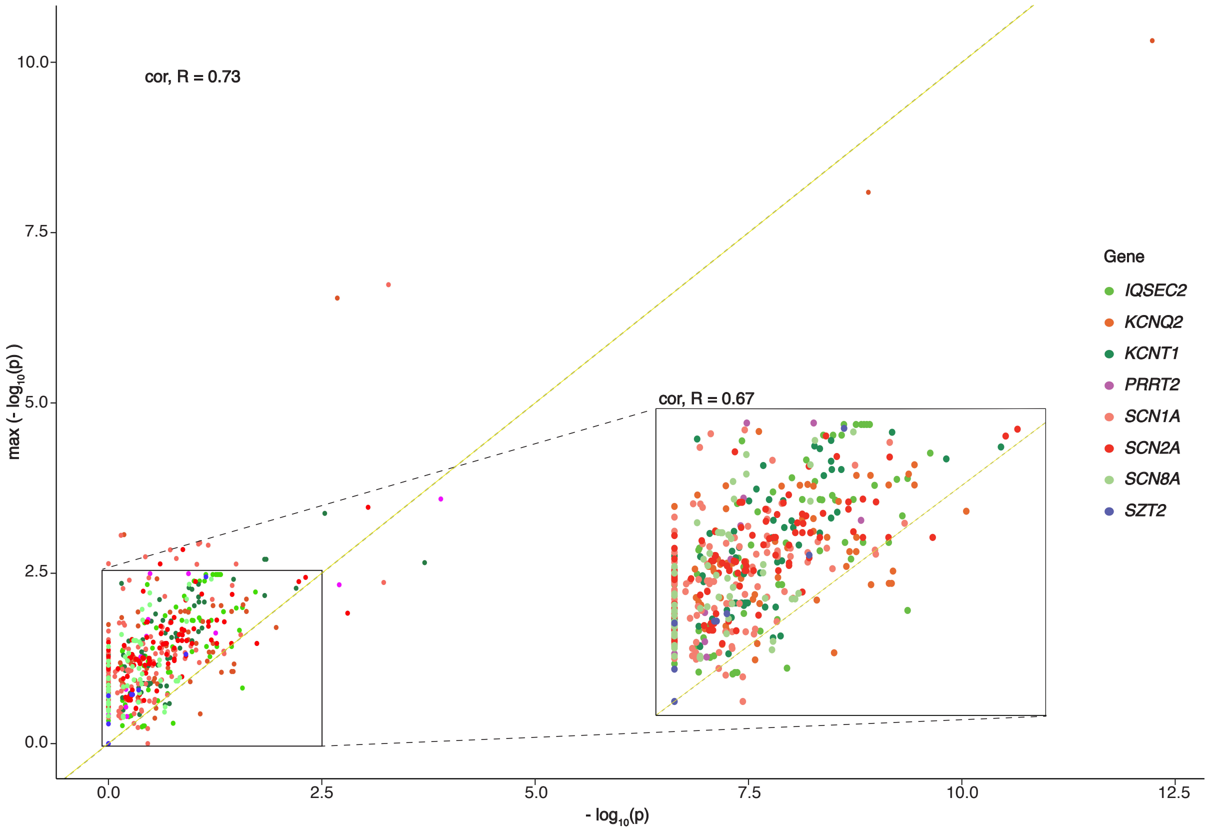

Figure S11. The correlation between time-dependent and collapsed genotype-phenotype information is moderate. X-axis demonstrates the p-value for a phenotypic features per gene when data for all 100 time periods is collapsed. Y-axis demonstrates the most significant association between each gene and each phenotypic feature, selecting the time point with the highest significance. P-values are shown as -$\log_{10}$, color represents genes. Time-dependent associations are generally more significant than collapsed data, and there is an overall moderate correlation.

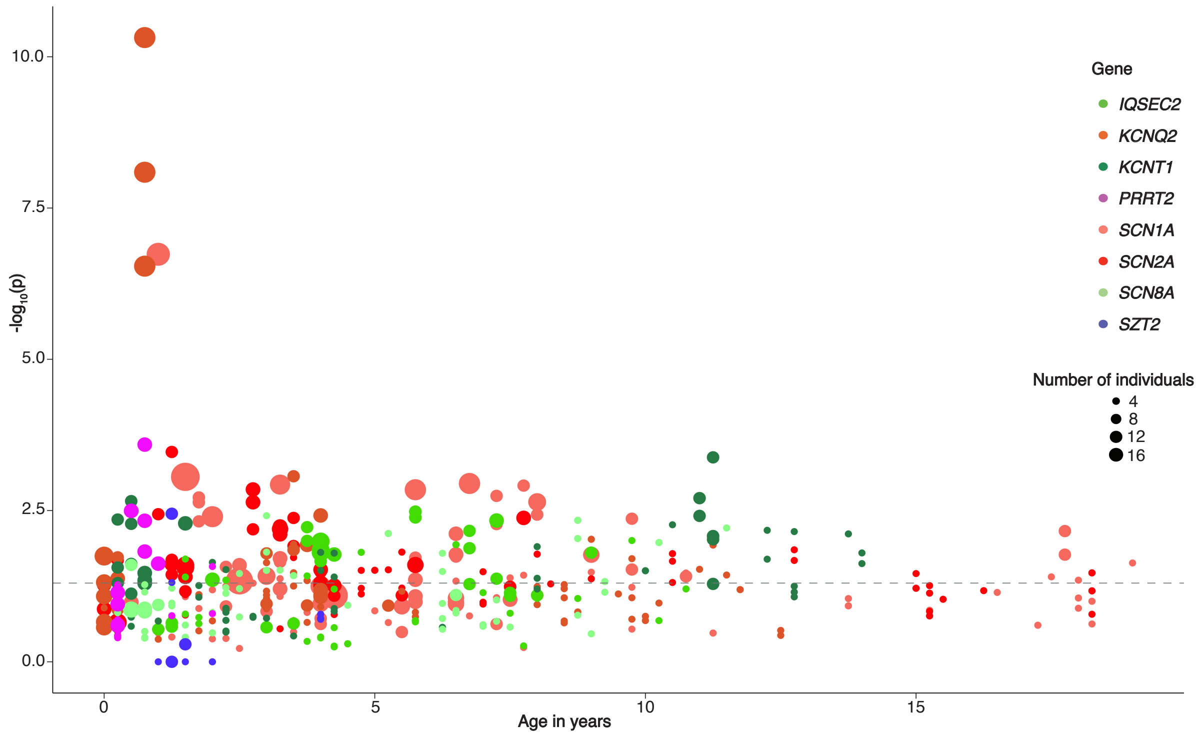

Figure S12. Patterns of significant gene-phenotype associations differ over time and are dependent on the number of individuals with a distinct gene. X-axis indicates age and y-axis indicates -$\log_{10}$ for gene-phenotype associations. Size of the bubble indicates number of individuals with a specific gene and color indicates gene. While significant associations require a larger number of individuals, non-significant associations are also observed with an equal number of individuals. Over time, both the significance as well as the number of individuals per gene decrease. We did not find a general bias towards more significant associations in time intervals with a higher number of individuals across all 528 terms.

Supplementary References

1. Resnik P, editor. Using information content to evaluate semantic similarity in a taxonomy. 14th International Joint Conference on Artificial Intelligence; 1995; Montreal: Morgan Kaufmann Publishers Inc. San Francisco, CA, USA.
2. Mignot C, McMahon AC, Bar C, et al. IQSEC2-related encephalopathy in males and females: a comparative study including 37 novel patients. Genet Med. 2019 Apr;21(4):837-49.
3. Radley JA, O'Sullivan RBG, Turton SE, et al. Deep phenotyping of 14 new patients with IQSEC2 variants, including monozygotic twins of discordant phenotype. Clin Genet. 2019 Apr;95(4):496-506.
4. Claes L, Del-Favero J, Ceulemans B, Lagae L, Van Broeckhoven C, De Jonghe P. De novo mutations in the sodium-channel gene SCN1A cause severe myoclonic epilepsy of infancy. Am J Hum Genet. 2001 Jun;68(6):1327-32.
5. Miller IO, Sotero de Menezes MA. SCN1A Seizure Disorders. In: Adam MP, Ardinger HH, Pagon RA, Wallace SE, Bean LJH, Stephens K, et al., editors. GeneReviews((R)). Seattle (WA)1993.

Additional supplementary figures

Phenotypic features associated with discrete genetic etiologies at specific time points when binned into 3-month intervals. Clinical terms associated with individual genes occur at different time intervals. The HPO terms associated with all the 36 genes identified in two or more individuals in our cohort are shown as an example with only the time interval with the most significant association for each HPO term shown. X-axis denotes patient age, y-axis denotes $-\log_{10}$ of the p-value (Fisher's exact test).

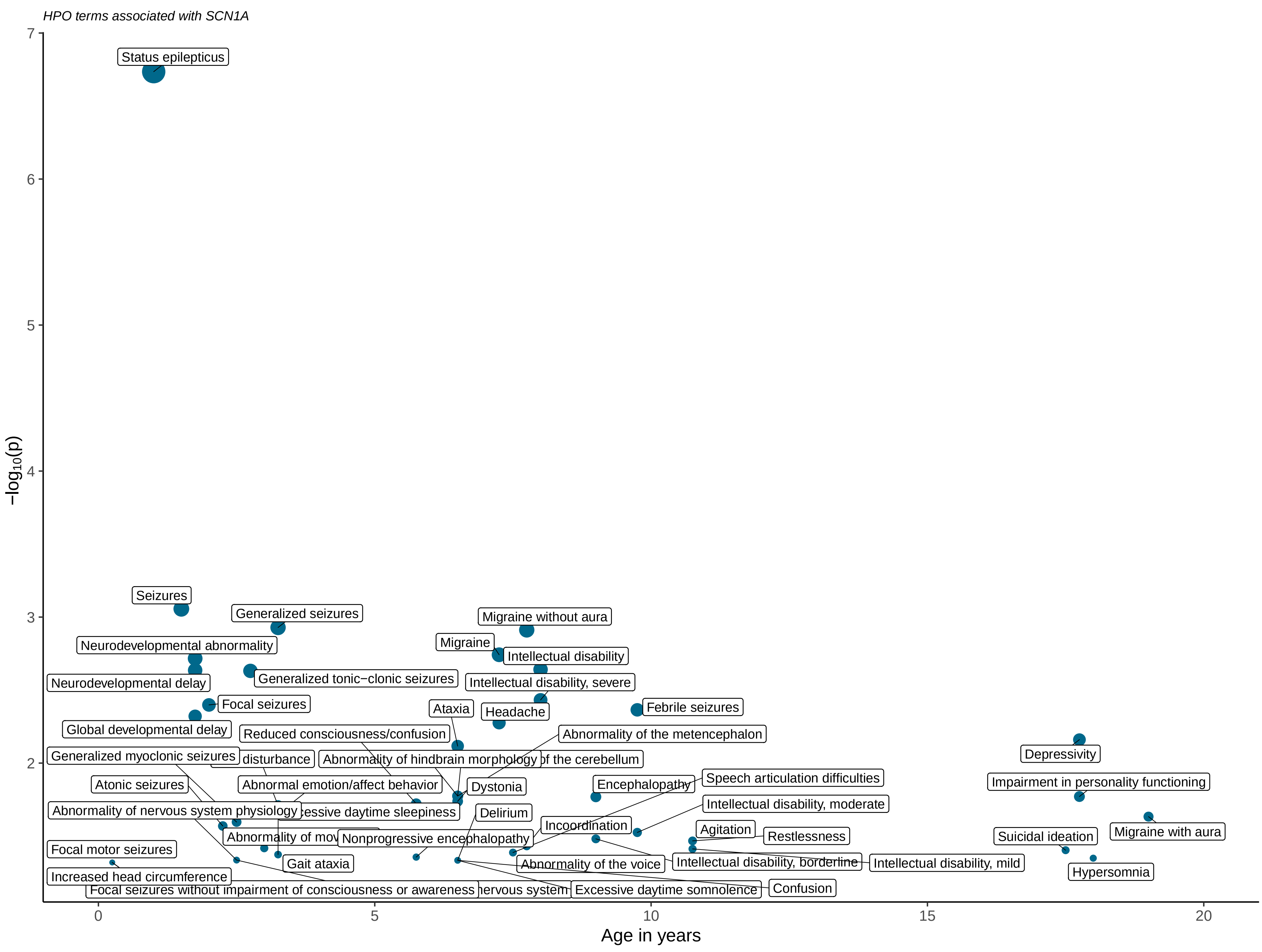

HPO terms associated with SCN1A
Status epilepticus
Seizures
Generalized seizures
Migraine without aura
Migraine
Neurodevelopmental abnormality
Intellectual disability
Neurodevelopmental delay
Generalized tonic−clonic seizures
Intellectual disability, severe
Focal seizures
Ataxia
Headache
Febrile seizures
Global developmental delay
Reduced consciousness/confusion
Abnormality of the metencephalon
Depressivity
Generalized myoclonic seizures
disturbance
Abnormality of hindbrain morphology
of the cerebellum
Encephalopathy
Speech articulation difficulties
Impairment in personality functioning
Atonic seizures
Abnormal emotion/affect behavior
Dystonia
Abnormality of nervous system physiology
essive daytime sleepiness
Delirium
Intellectual disability, moderate
Incoordination
Agitation
Restlessness
Suicidal ideation
Migraine with aura
Abnormality of mov
Nonprogressive encephalopathy
Focal motor seizures
Gait ataxia
Abnormality of the voice
Intellectual disability, borderline
Intellectual disability, mild
Hypersomnia
Increased head circumference
Focal seizures without impairment of consciousness or awareness
nervous system
Excessive daytime somnolence
Confusion
−log10(p)
Age in years
7
6
5
4
3
2
0
5
10
15
20

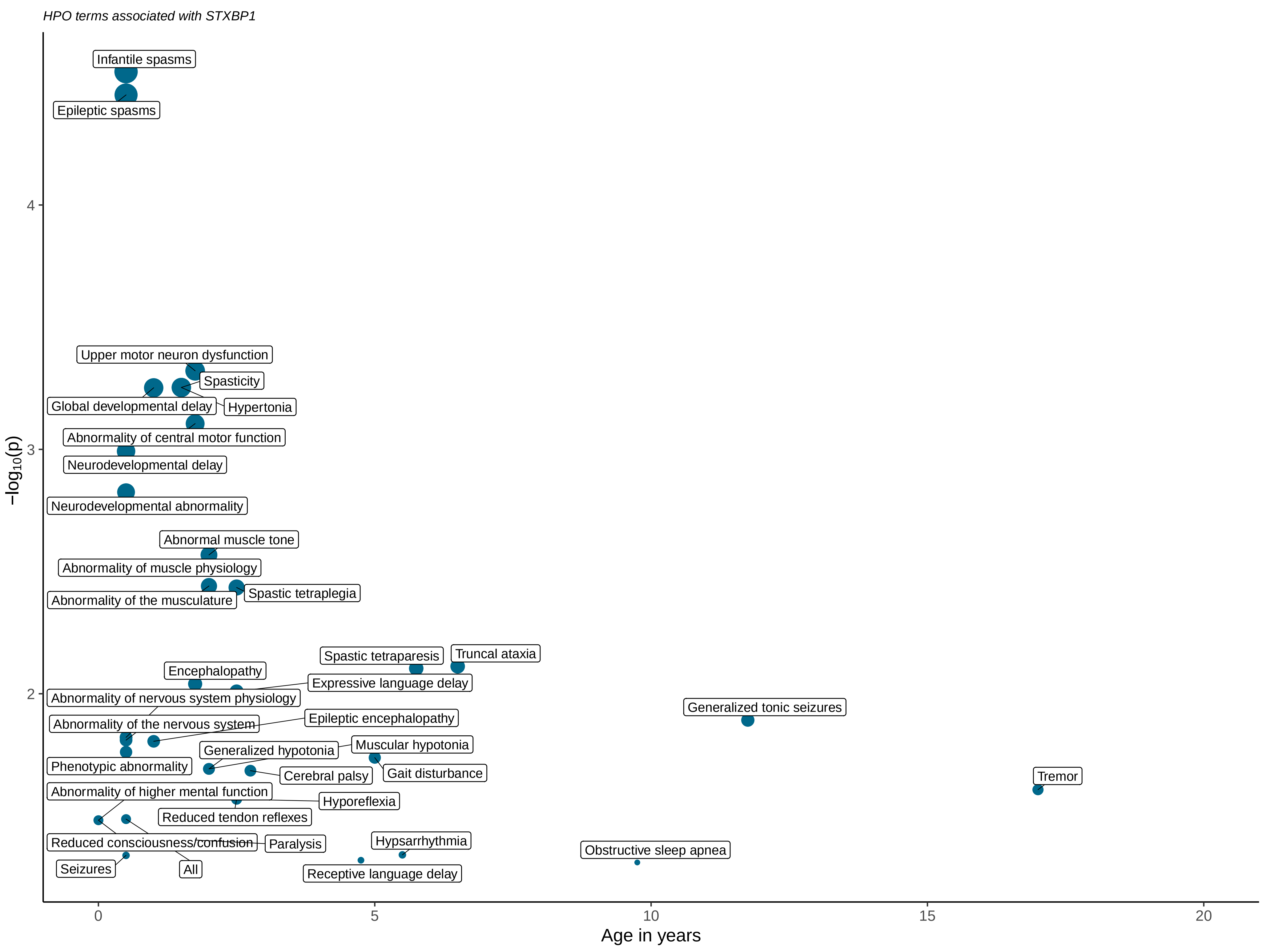

HPO terms associated with STXBP1
Infantile spasms
Epileptic spasms
Upper motor neuron dysfunction
Spasticity
Global developmental delay
Hypertonia
Abnormality of central motor function
Neurodevelopmental delay
Neurodevelopmental abnormality
Abnormal muscle tone
Abnormality of muscle physiology
Abnormality of the musculature
Spastic tetraplegia
Spastic tetraparesis
Truncal ataxia
Encephalopathy
Expressive language delay
Abnormality of nervous system physiology
Generalized tonic seizures
Abnormality of the nervous system
Epileptic encephalopathy
Generalized hypotonia
Muscular hypotonia
Phenotypic abnormality
Cerebral palsy
Gait disturbance
Tremor
Abnormality of higher mental function
Hyporeflexia
Reduced tendon reflexes
Reduced consciousness/confusion
Paralysis
Hypsarrhythmia
Obstructive sleep apnea
Seizures
All
Receptive language delay
−log10(p)
4
3
2
0
5
10
15
20
Age in years

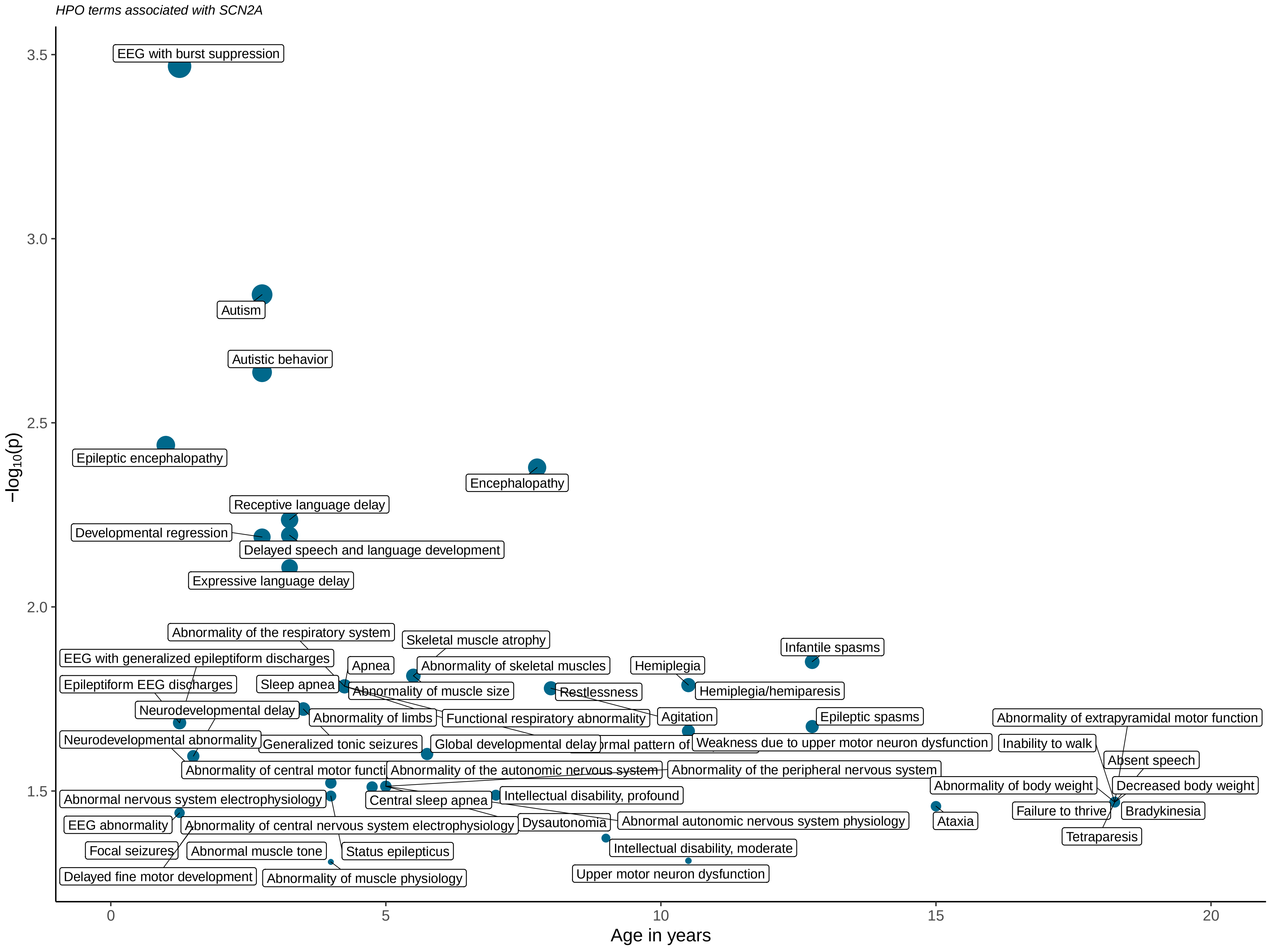
HPO terms associated with SCN2A
−log10(p)
3.5
3.0
2.5
2.0
1.5
EEG with burst suppression
Autism
Autistic behavior
Epileptic encephalopathy
Encephalopathy
Receptive language delay
Developmental regression
Delayed speech and language development
Expressive language delay
Abnormality of the respiratory system
Skeletal muscle atrophy
Infantile spasms
EEG with generalized epileptiform discharges
Apnea
Abnormality of skeletal muscles
Hemiplegia
Epileptiform EEG discharges
Sleep apnea
Abnormality of muscle size
Restlessness
Hemiplegia/hemiparesis
Neurodevelopmental delay
Abnormality of limbs
Functional respiratory abnormality
Agitation
Epileptic spasms
Abnormality of extrapyramidal motor function
Neurodevelopmental abnormality
Generalized tonic seizures
Global developmental delay
Weakness due to upper motor neuron dysfunction
Inability to walk
Absent speech
Abnormality of the autonomic nervous system
Abnormality of the peripheral nervous system
Abnormality of body weight
Decreased body weight
Abnormal nervous system electrophysiology
Central sleep apnea
Intellectual disability, profound
Failure to thrive
Bradykinesia
EEG abnormality
Abnormality of central nervous system electrophysiology
Dysautonomia
Abnormal autonomic nervous system physiology
Ataxia
Tetraparesis
Focal seizures
Abnormal muscle tone
Status epilepticus
Intellectual disability, moderate
Delayed fine motor development
Abnormality of muscle physiology
Upper motor neuron dysfunction
0
5
10
15
20
Age in years

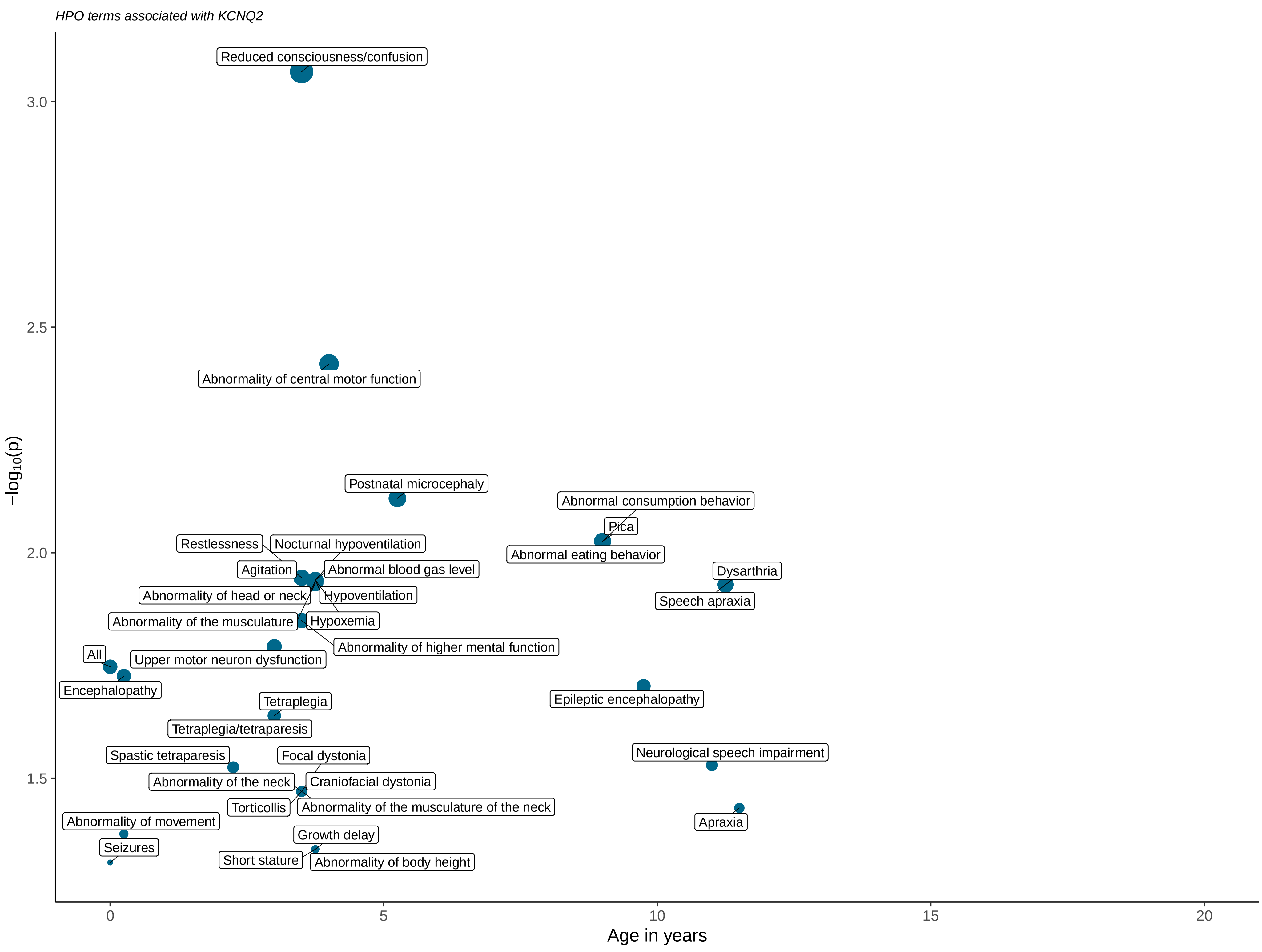

HPO terms associated with KCNQ2
Reduced consciousness/confusion
Abnormality of central motor function
Postnatal microcephaly
Abnormal consumption behavior
Pica
Abnormal eating behavior
Restlessness
Nocturnal hypoventilation
Agitation
Abnormal blood gas level
Abnormality of head or neck
Hypoventilation
Abnormality of the musculature
Hypoxemia
Abnormality of higher mental function
Dysarthria
Speech apraxia
All
Upper motor neuron dysfunction
Encephalopathy
Epileptic encephalopathy
Tetraplegia
Tetraplegia/tetraparesis
Spastic tetraparesis
Focal dystonia
Neurological speech impairment
Abnormality of the neck
Craniofacial dystonia
Torticollis
Abnormality of the musculature of the neck
Apraxia
Abnormality of movement
Seizures
Growth delay
Short stature
Abnormality of body height
−log10(p)
3.0
2.5
2.0
1.5
0
5
10
15
20
Age in years

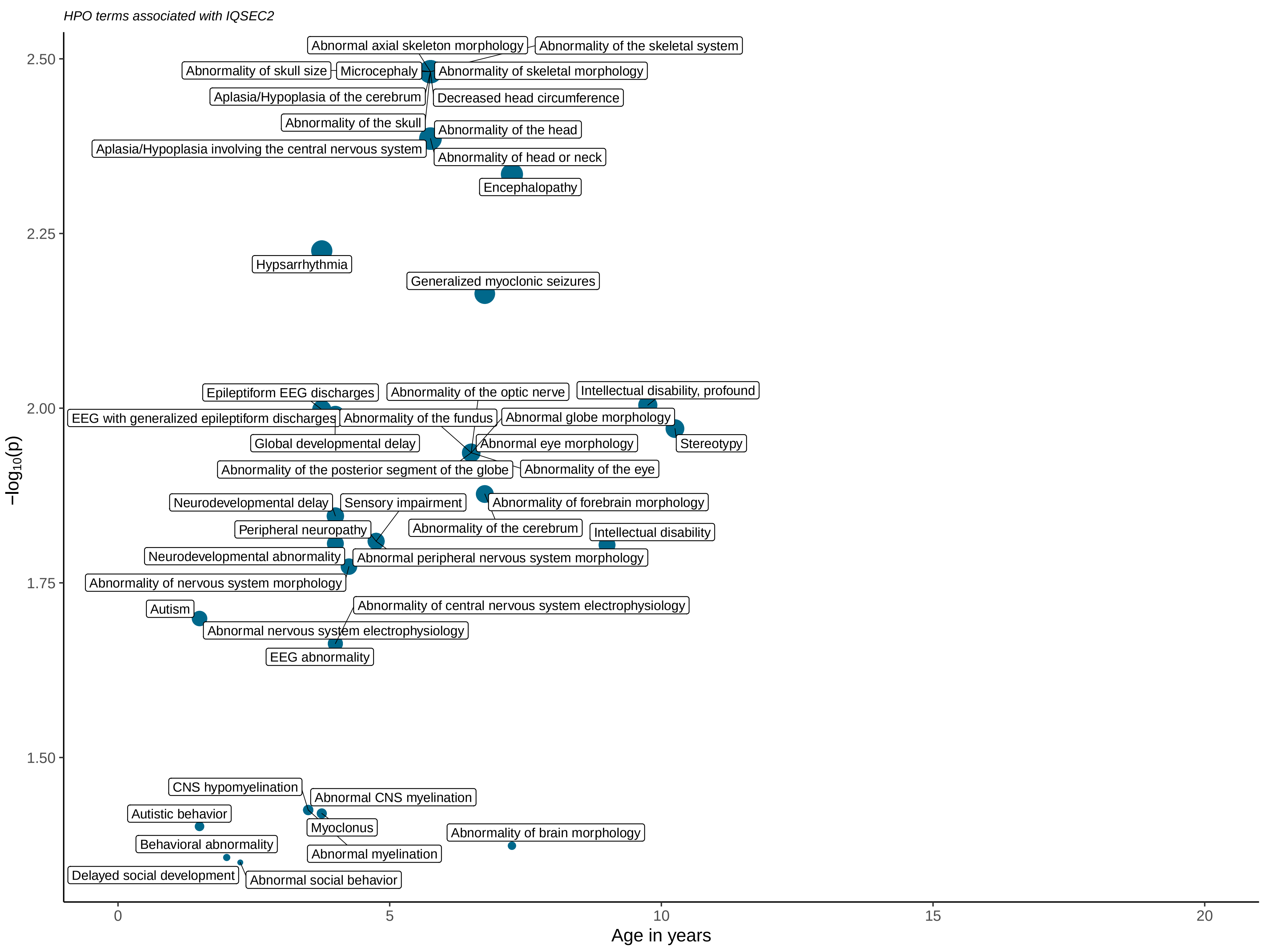

HPO terms associated with IQSEC2
Abnormal axial skeleton morphology
Abnormality of the skeletal system
Abnormality of skull size
Microcephaly
Abnormality of skeletal morphology
Aplasia/Hypoplasia of the cerebrum
Decreased head circumference
Abnormality of the skull
Abnormality of the head
Aplasia/Hypoplasia involving the central nervous system
Abnormality of head or neck
Encephalopathy
Hypsarrhythmia
Generalized myoclonic seizures
Epileptiform EEG discharges
Abnormality of the optic nerve
Intellectual disability, profound
EEG with generalized epileptiform discharges
Abnormality of the fundus
Abnormal globe morphology
Global developmental delay
Abnormal eye morphology
Stereotypy
Abnormality of the posterior segment of the globe
Abnormality of the eye
Neurodevelopmental delay
Sensory impairment
Abnormality of forebrain morphology
Peripheral neuropathy
Abnormality of the cerebrum
Intellectual disability
Neurodevelopmental abnormality
Abnormal peripheral nervous system morphology
Abnormality of nervous system morphology
Abnormality of central nervous system electrophysiology
Autism
Abnormal nervous system electrophysiology
EEG abnormality
CNS hypomyelination
Abnormal CNS myelination
Autistic behavior
Myoclonus
Abnormality of brain morphology
Behavioral abnormality
Abnormal myelination
Delayed social development
Abnormal social behavior
2.50
2.25
2.00
1.75
1.50
−log10(p)
0
5
10
15
20
Age in years

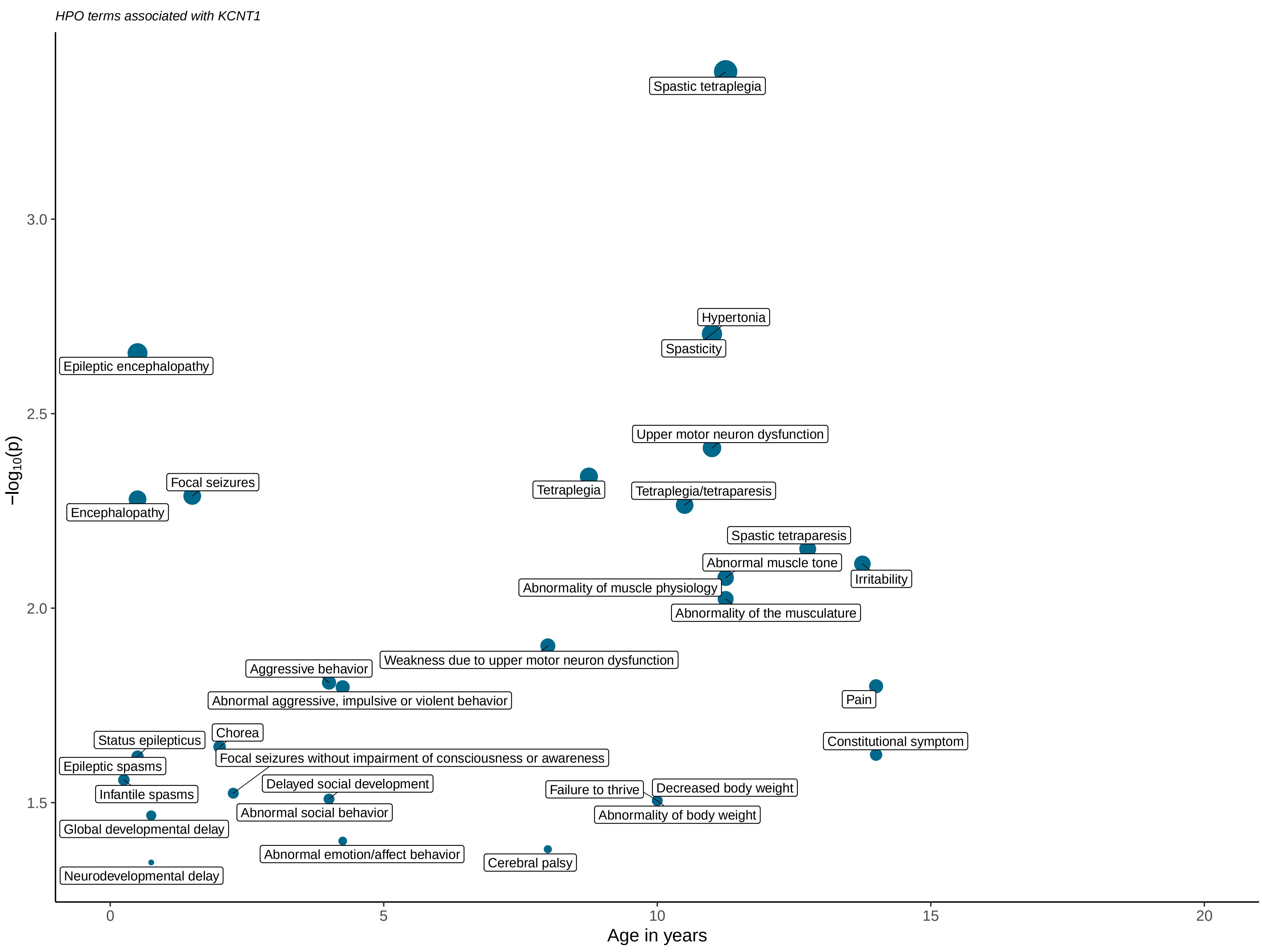
HPO terms associated with KCNT1
Spastic tetraplegia
Hypertonia
Spasticity
Epileptic encephalopathy
Upper motor neuron dysfunction
Focal seizures
Encephalopathy
Tetraplegia
Tetraplegia/tetraparesis
Spastic tetraparesis
Abnormal muscle tone
Irritability
Abnormality of muscle physiology
Abnormality of the musculature
Weakness due to upper motor neuron dysfunction
Aggressive behavior
Abnormal aggressive, impulsive or violent behavior
Pain
Chorea
Status epilepticus
Constitutional symptom
Focal seizures without impairment of consciousness or awareness
Epileptic spasms
Delayed social development
Infantile spasms
Failure to thrive
Decreased body weight
Abnormal social behavior
Abnormality of body weight
Global developmental delay
Abnormal emotion/affect behavior
Cerebral palsy
Neurodevelopmental delay
−log10(p)
3.0
2.5
2.0
1.5
0
5
10
15
20
Age in years

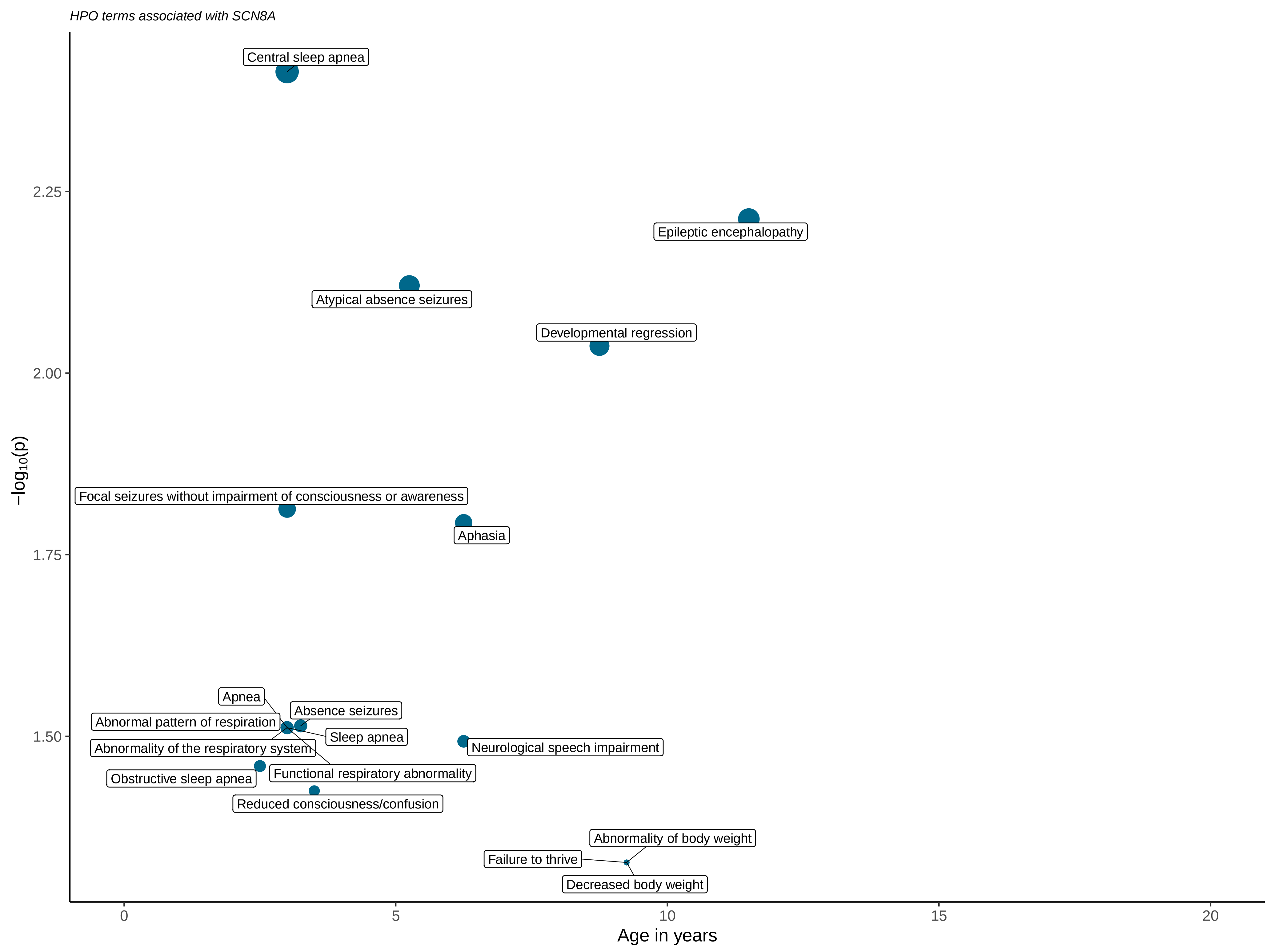

HPO terms associated with SCN8A
Central sleep apnea
Epileptic encephalopathy
Atypical absence seizures
Developmental regression
Focal seizures without impairment of consciousness or awareness
Aphasia
Apnea
Absence seizures
Abnormal pattern of respiration
Sleep apnea
Abnormality of the respiratory system
Neurological speech impairment
Obstructive sleep apnea
Functional respiratory abnormality
Reduced consciousness/confusion
Abnormality of body weight
Failure to thrive
Decreased body weight
2.25
2.00
1.75
1.50
−log10(p)
0
5
10
15
20
Age in years

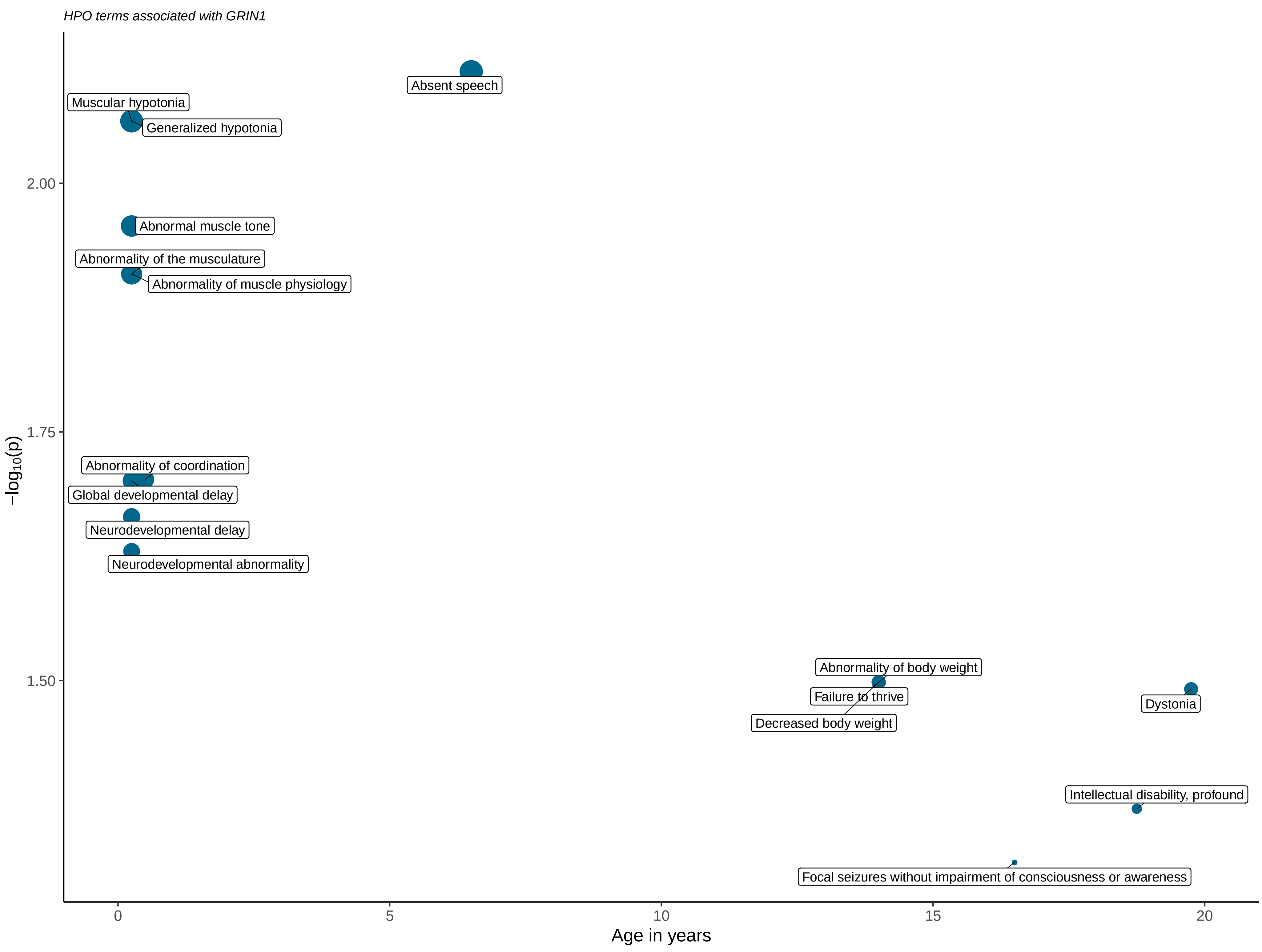

HPO terms associated with GRIN1
−log10(p)
2.00
1.75
1.50
Absent speech
Muscular hypotonia
Generalized hypotonia
Abnormal muscle tone
Abnormality of the musculature
Abnormality of muscle physiology
Abnormality of coordination
Global developmental delay
Neurodevelopmental delay
Neurodevelopmental abnormality
Abnormality of body weight
Failure to thrive
Decreased body weight
Dystonia
Intellectual disability, profound
Focal seizures without impairment of consciousness or awareness
0
5
10
15
20
Age in years

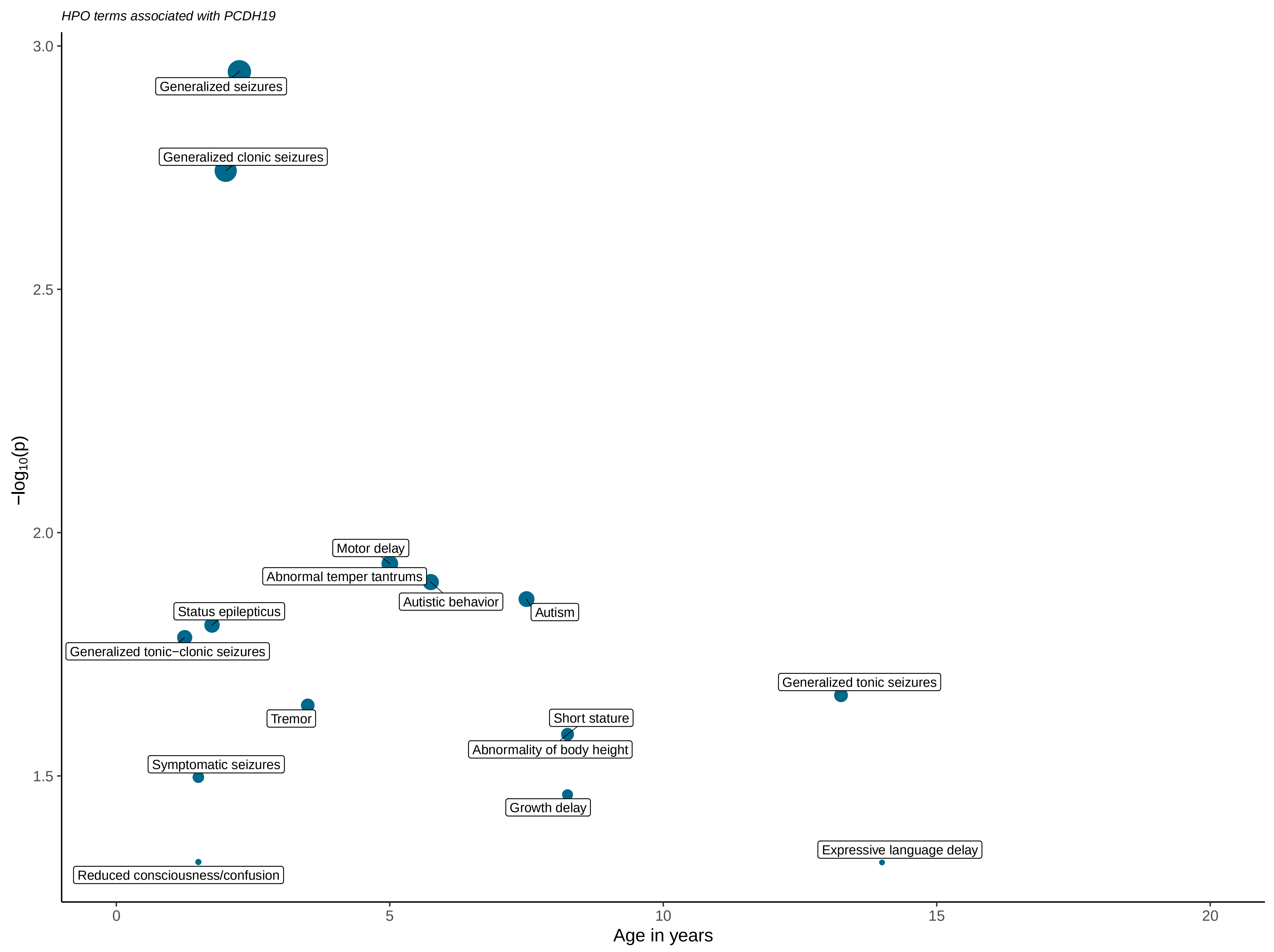
HPO terms associated with PCDH19
Generalized seizures
Generalized clonic seizures
Motor delay
Abnormal temper tantrums
Autistic behavior
Autism
Status epilepticus
Generalized tonic−clonic seizures
Generalized tonic seizures
Tremor
Short stature
Abnormality of body height
Symptomatic seizures
Growth delay
Expressive language delay
Reduced consciousness/confusion
−log10(p)
3.0
2.5
2.0
1.5
0
5
10
15
20
Age in years

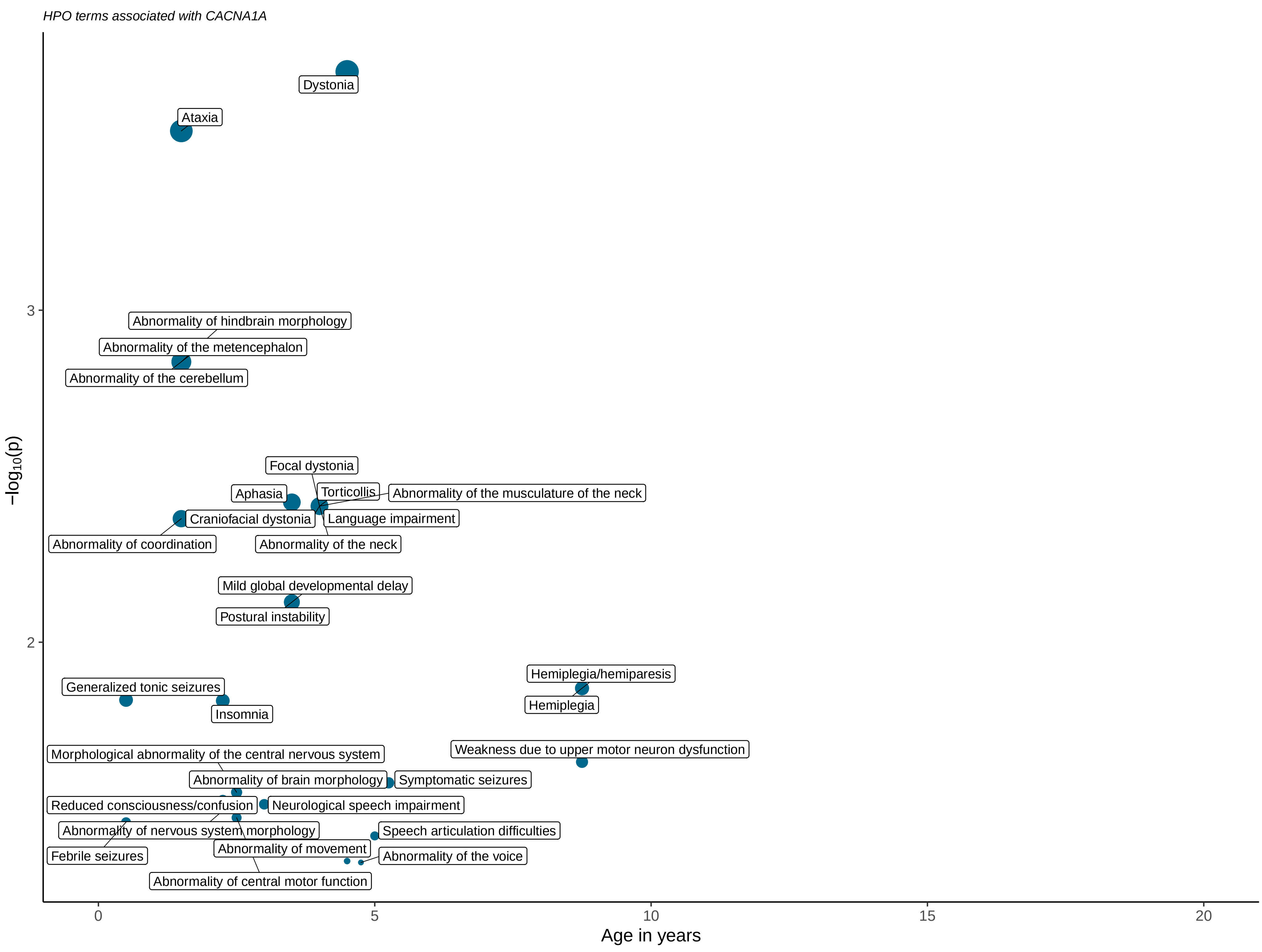

HPO terms associated with CACNA1A
Dystonia
Ataxia
Abnormality of hindbrain morphology
Abnormality of the metencephalon
Abnormality of the cerebellum
Focal dystonia
Aphasia
Torticollis
Abnormality of the musculature of the neck
Craniofacial dystonia
Language impairment
Abnormality of coordination
Abnormality of the neck
Mild global developmental delay
Postural instability
Hemiplegia/hemiparesis
Generalized tonic seizures
Hemiplegia
Insomnia
Weakness due to upper motor neuron dysfunction
Morphological abnormality of the central nervous system
Abnormality of brain morphology
Symptomatic seizures
Reduced consciousness/confusion
Neurological speech impairment
Abnormality of nervous system morphology
Speech articulation difficulties
Abnormality of movement
Febrile seizures
Abnormality of the voice
Abnormality of central motor function
−log10(p)
3
2
0
5
10
15
20
Age in years

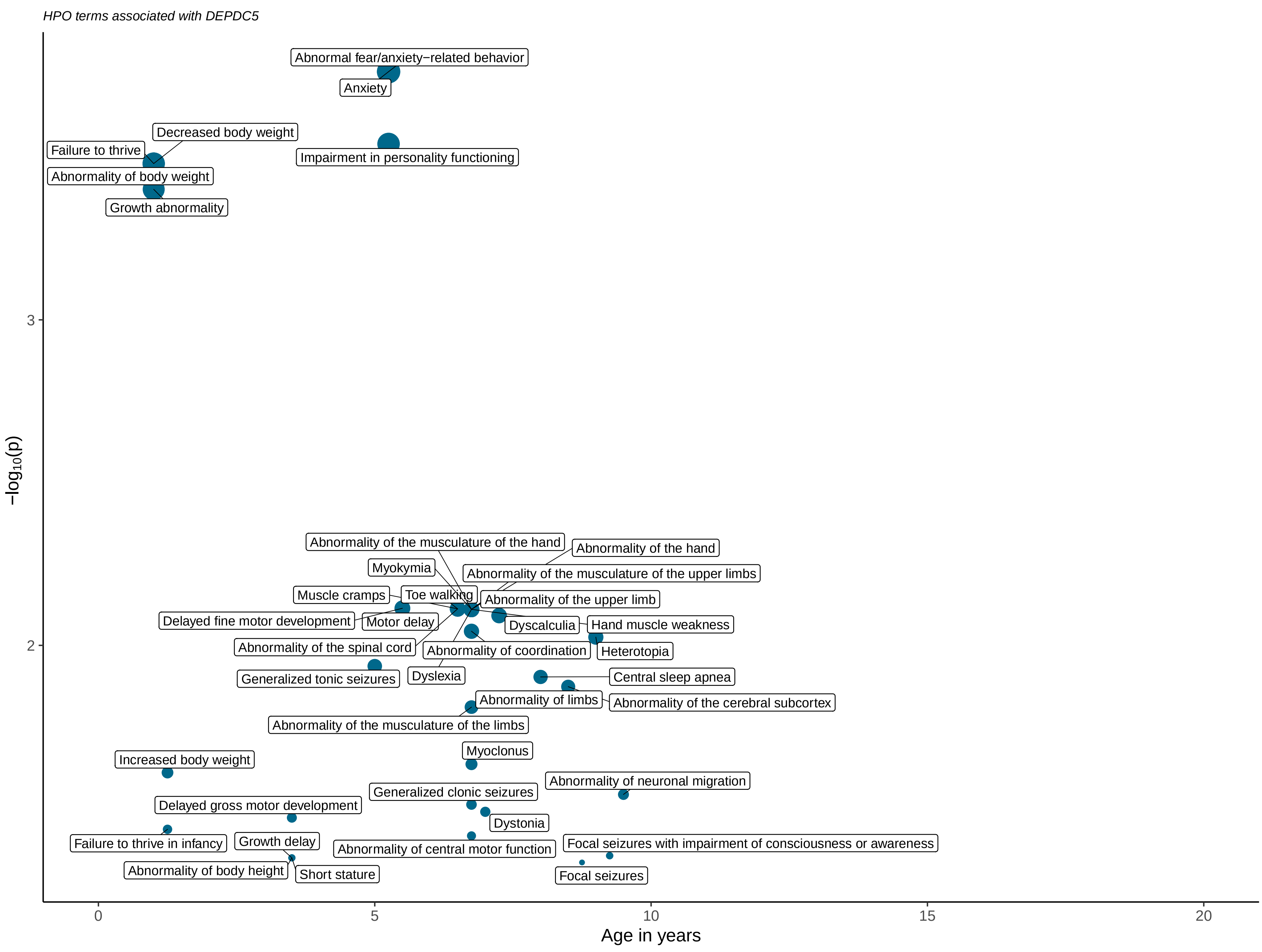

HPO terms associated with DEPDC5
Abnormal fear/anxiety−related behavior
Anxiety
Decreased body weight
Failure to thrive
Impairment in personality functioning
Abnormality of body weight
Growth abnormality
3
−log10(p)
Abnormality of the musculature of the hand
Abnormality of the hand
Myokymia
Abnormality of the musculature of the upper limbs
Muscle cramps
Toe walking
Abnormality of the upper limb
Delayed fine motor development
Motor delay
Dyscalculia
Hand muscle weakness
2
Abnormality of the spinal cord
Abnormality of coordination
Heterotopia
Generalized tonic seizures
Dyslexia
Central sleep apnea
Abnormality of limbs
Abnormality of the cerebral subcortex
Abnormality of the musculature of the limbs
Myoclonus
Increased body weight
Abnormality of neuronal migration
Generalized clonic seizures
Delayed gross motor development
Dystonia
Failure to thrive in infancy
Growth delay
Abnormality of central motor function
Focal seizures with impairment of consciousness or awareness
Abnormality of body height
Short stature
Focal seizures
0
5
10
15
20
Age in years

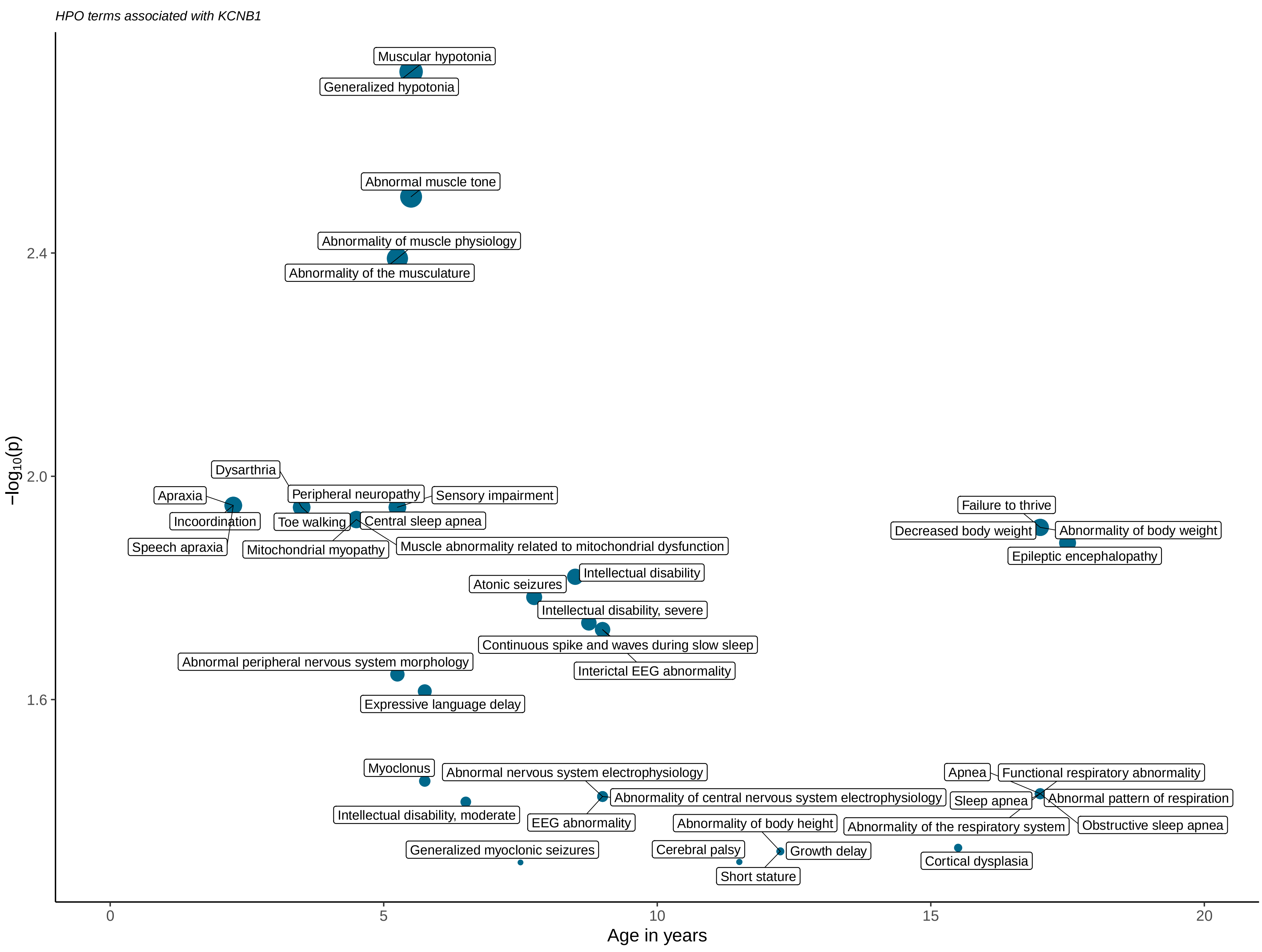

HPO terms associated with KCNB1
Muscular hypotonia
Generalized hypotonia
Abnormal muscle tone
Abnormality of muscle physiology
Abnormality of the musculature
Dysarthria
Apraxia
Incoordination
Speech apraxia
Peripheral neuropathy
Sensory impairment
Toe walking
Central sleep apnea
Mitochondrial myopathy
Muscle abnormality related to mitochondrial dysfunction
Failure to thrive
Decreased body weight
Abnormality of body weight
Epileptic encephalopathy
Intellectual disability
Atonic seizures
Intellectual disability, severe
Continuous spike and waves during slow sleep
Interictal EEG abnormality
Abnormal peripheral nervous system morphology
Expressive language delay
Myoclonus
Abnormal nervous system electrophysiology
Abnormality of central nervous system electrophysiology
Intellectual disability, moderate
EEG abnormality
Generalized myoclonic seizures
Abnormality of body height
Cerebral palsy
Growth delay
Short stature
Apnea
Functional respiratory abnormality
Sleep apnea
Abnormal pattern of respiration
Abnormality of the respiratory system
Obstructive sleep apnea
Cortical dysplasia
−log10(p)
2.4
2.0
1.6
0
5
10
15
20
Age in years

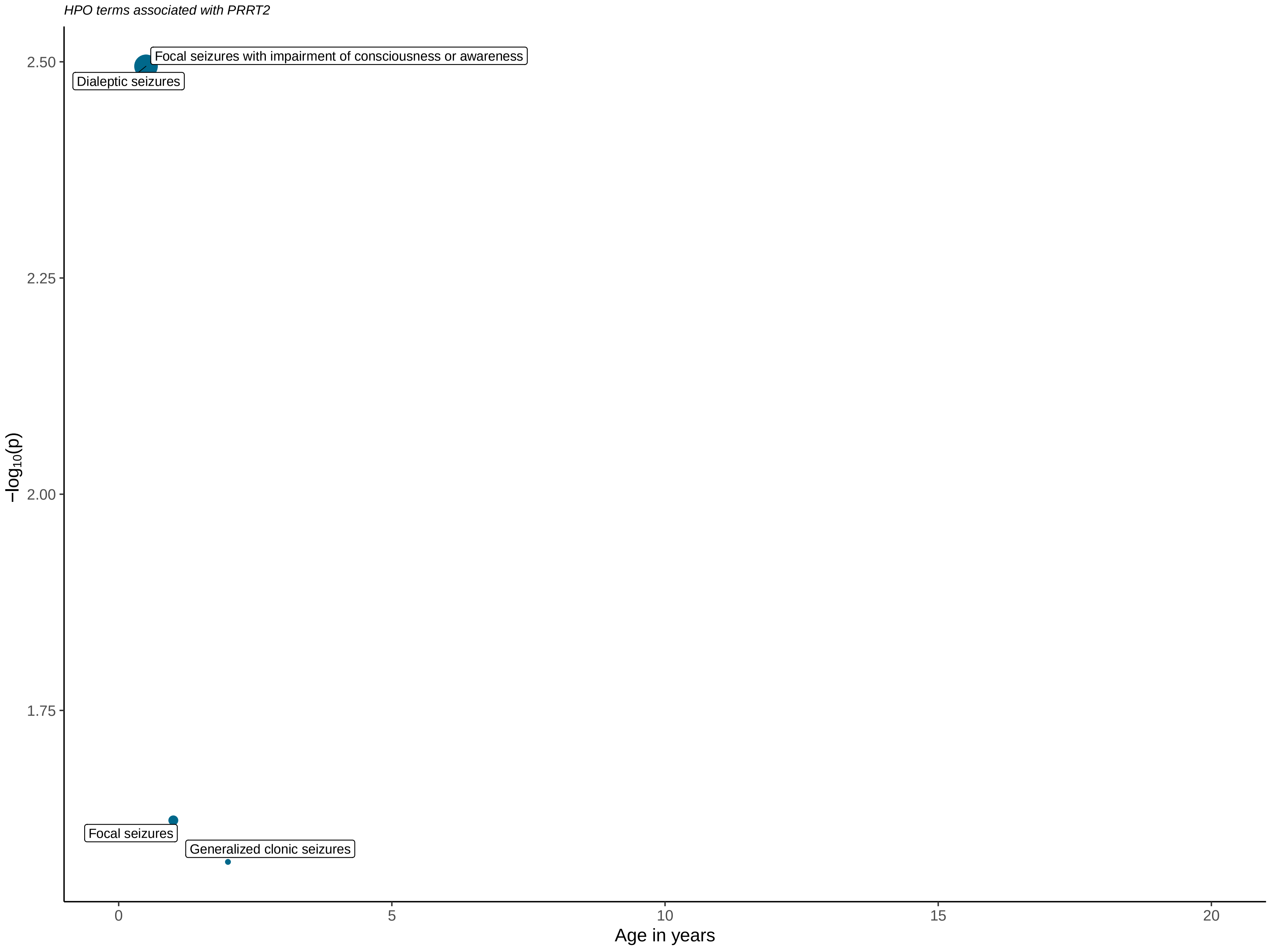
HPO terms associated with PRRT2
Focal seizures with impairment of consciousness or awareness
Dialeptic seizures
Focal seizures
Generalized clonic seizures
2.50
2.25
2.00
1.75
−log10(p)
0
5
10
15
20
Age in years

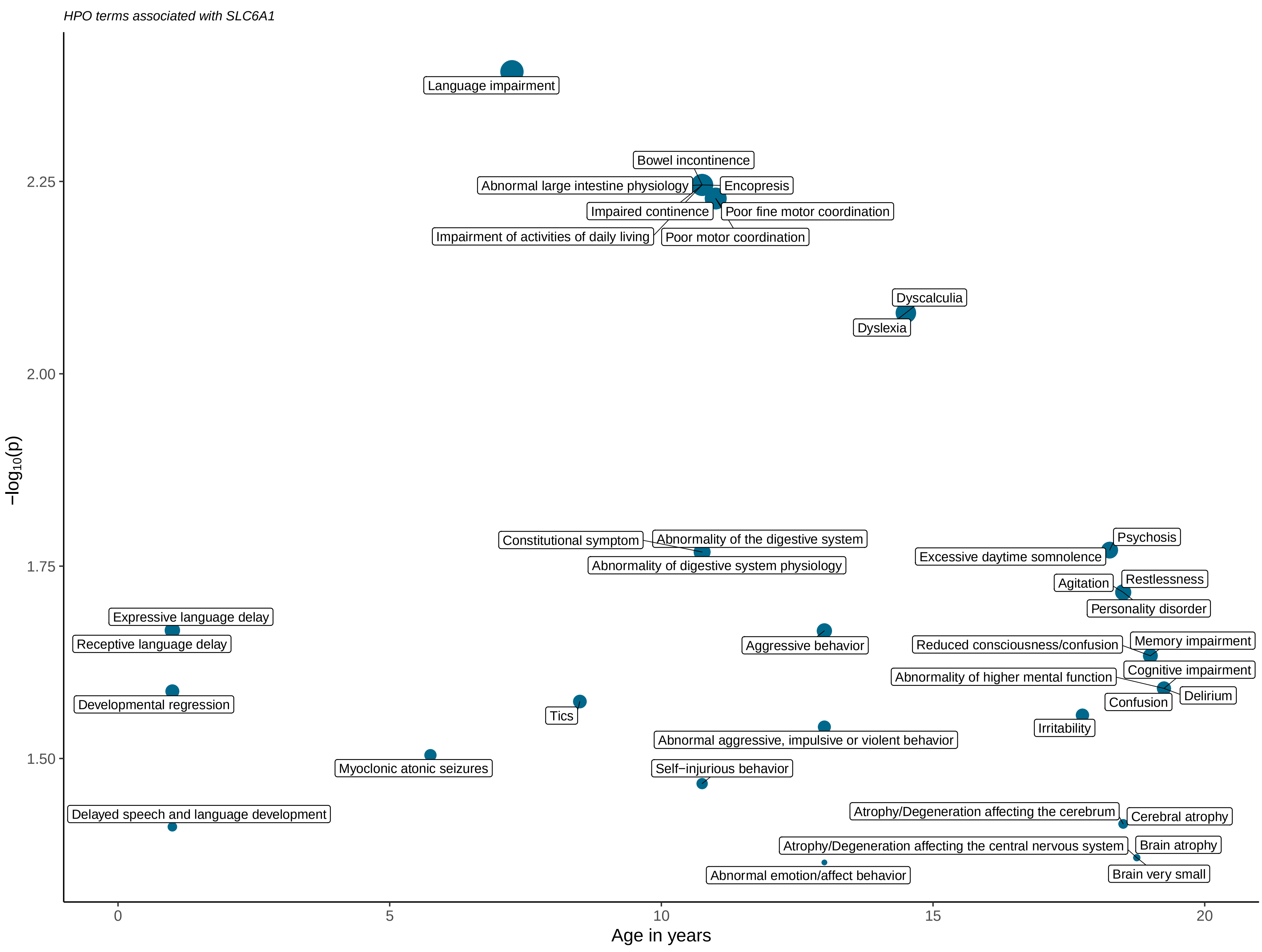
HPO terms associated with SLC6A1
Language impairment
Bowel incontinence
Abnormal large intestine physiology
Encopresis
Impaired continence
Poor fine motor coordination
Impairment of activities of daily living
Poor motor coordination
Dyscalculia
Dyslexia
Constitutional symptom
Abnormality of the digestive system
Abnormality of digestive system physiology
Psychosis
Excessive daytime somnolence
Agitation
Restlessness
Personality disorder
Expressive language delay
Receptive language delay
Aggressive behavior
Reduced consciousness/confusion
Memory impairment
Cognitive impairment
Abnormality of higher mental function
Delirium
Confusion
Developmental regression
Tics
Irritability
Abnormal aggressive, impulsive or violent behavior
Myoclonic atonic seizures
Self−injurious behavior
Delayed speech and language development
Atrophy/Degeneration affecting the cerebrum
Cerebral atrophy
Atrophy/Degeneration affecting the central nervous system
Brain atrophy
Abnormal emotion/affect behavior
Brain very small
−log10(p)
2.25
2.00
1.75
1.50
0
5
10
15
20
Age in years

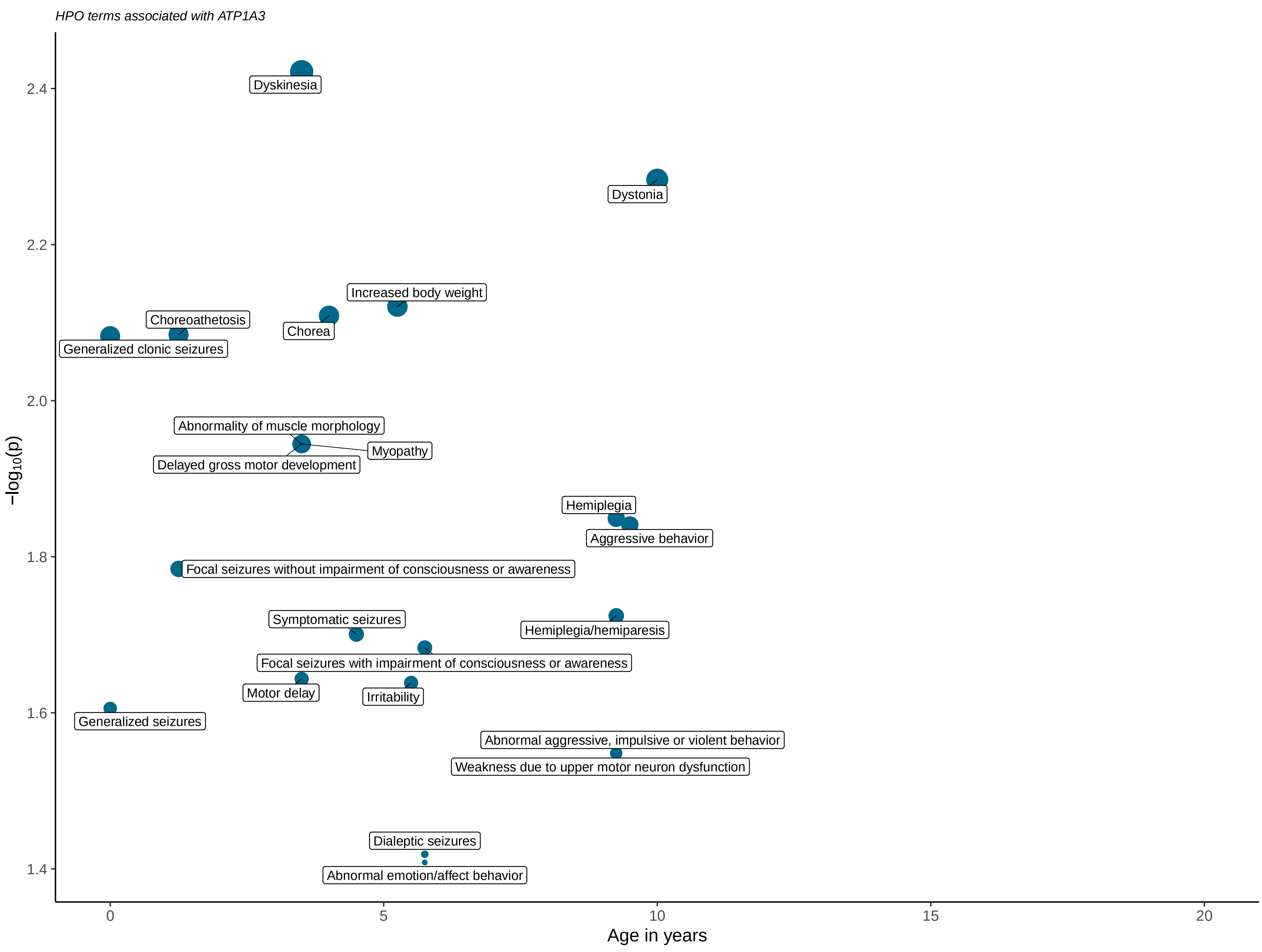
HPO terms associated with ATP1A3
Dyskinesia
Dystonia
Increased body weight
Choreoathetosis
Chorea
Generalized clonic seizures
Abnormality of muscle morphology
Myopathy
Delayed gross motor development
Hemiplegia
Aggressive behavior
Focal seizures without impairment of consciousness or awareness
Symptomatic seizures
Hemiplegia/hemiparesis
Focal seizures with impairment of consciousness or awareness
Motor delay
Irritability
Generalized seizures
Abnormal aggressive, impulsive or violent behavior
Weakness due to upper motor neuron dysfunction
Dialeptic seizures
Abnormal emotion/affect behavior
$-\log_{10}(p)$
2.4
2.2
2.0
1.8
1.6
1.4
0
5
10
15
20
Age in years

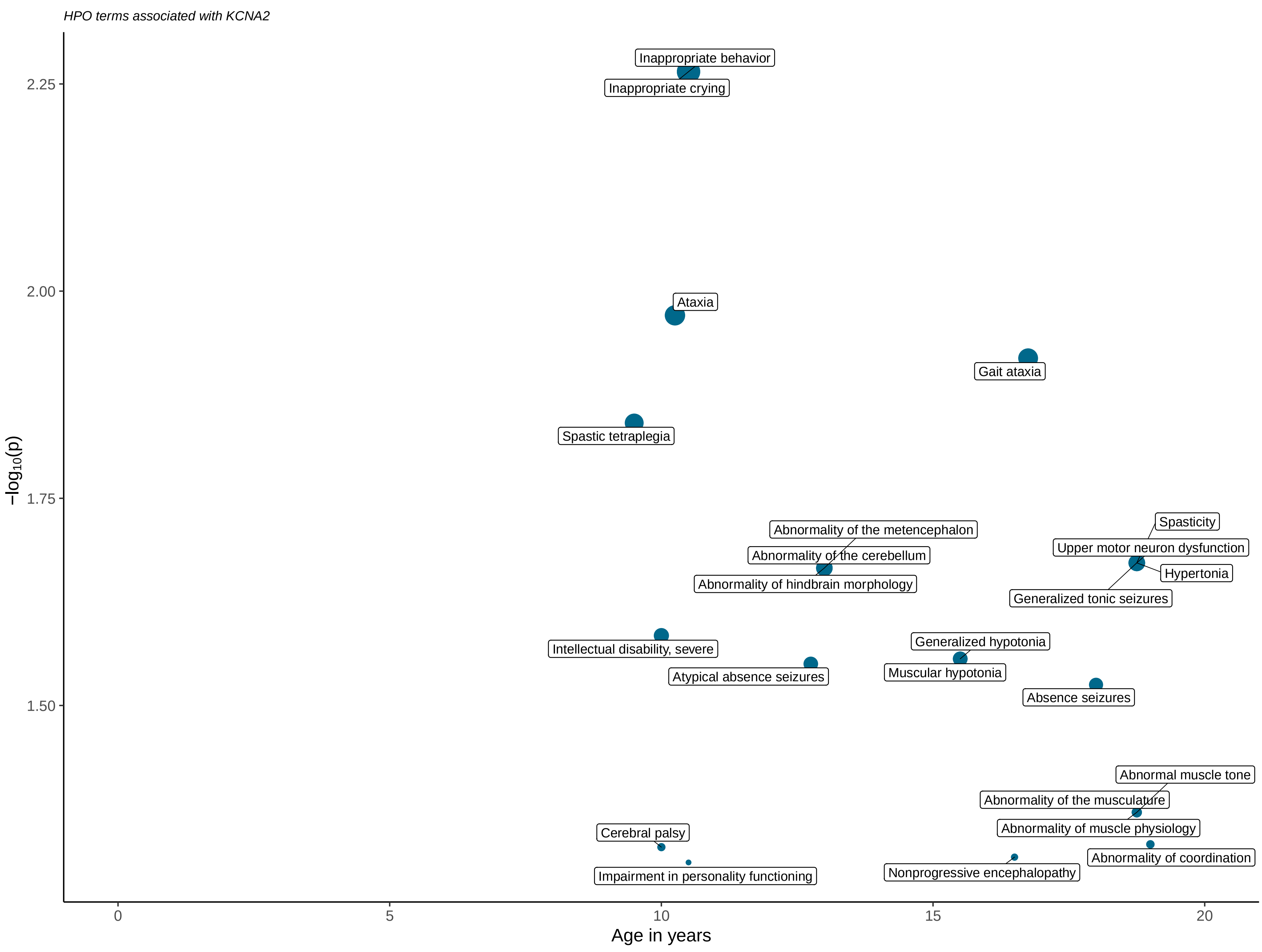

HPO terms associated with KCNA2
Inappropriate behavior
Inappropriate crying
Ataxia
Gait ataxia
Spastic tetraplegia
Abnormality of the metencephalon
Abnormality of the cerebellum
Abnormality of hindbrain morphology
Spasticity
Upper motor neuron dysfunction
Hypertonia
Generalized tonic seizures
Intellectual disability, severe
Atypical absence seizures
Generalized hypotonia
Muscular hypotonia
Absence seizures
Abnormal muscle tone
Abnormality of the musculature
Abnormality of muscle physiology
Abnormality of coordination
Cerebral palsy
Impairment in personality functioning
Nonprogressive encephalopathy
−log10(p)
2.25
2.00
1.75
1.50
0
5
10
15
20
Age in years

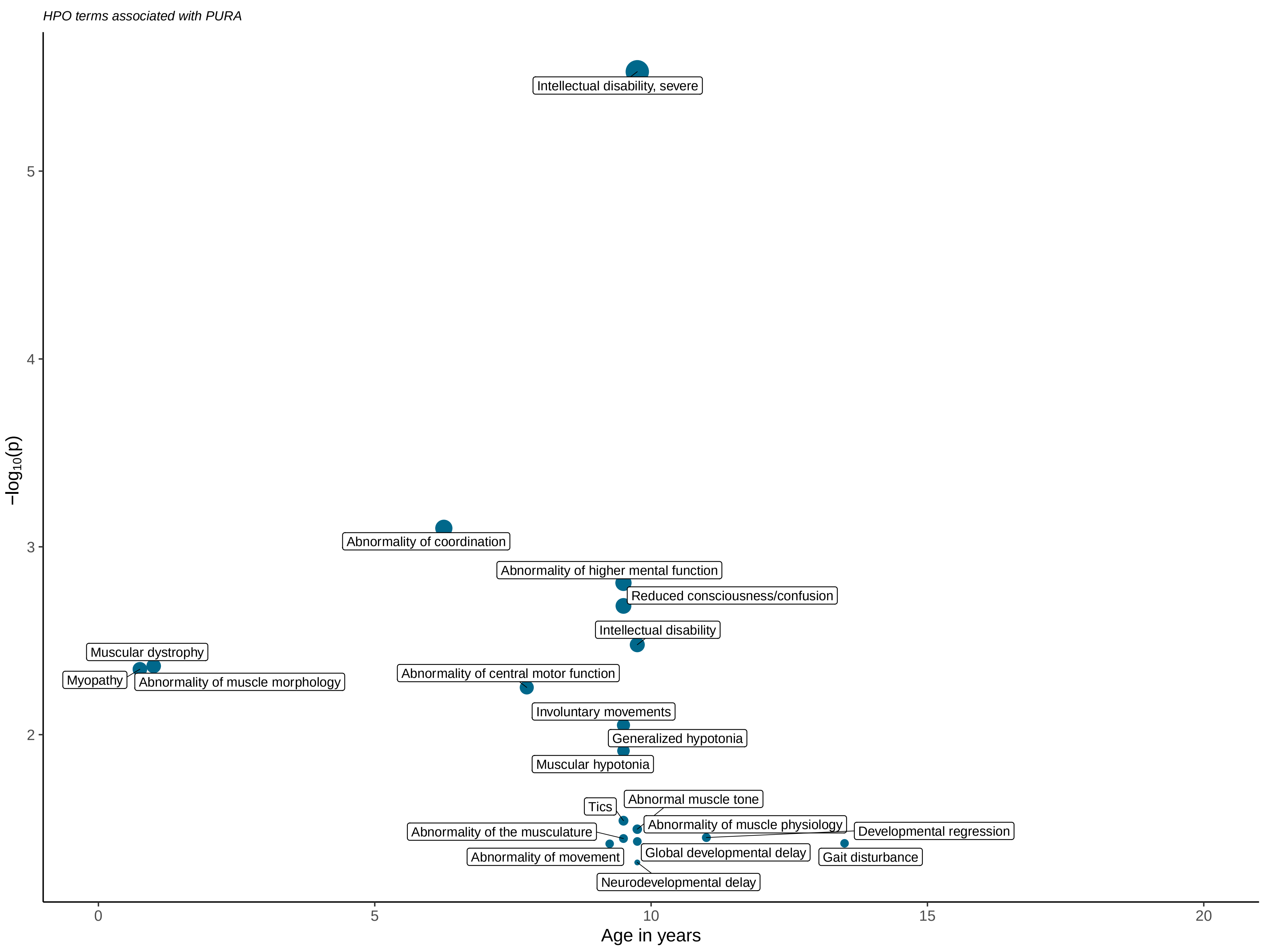
HPO terms associated with PURA
Intellectual disability, severe
Abnormality of coordination
Abnormality of higher mental function
Reduced consciousness/confusion
Intellectual disability
Muscular dystrophy
Myopathy
Abnormality of muscle morphology
Abnormality of central motor function
Involuntary movements
Generalized hypotonia
Muscular hypotonia
Tics
Abnormal muscle tone
Abnormality of muscle physiology
Abnormality of the musculature
Developmental regression
Abnormality of movement
Global developmental delay
Gait disturbance
Neurodevelopmental delay
−log10(p)
2
3
4
5
0
5
10
15
20
Age in years

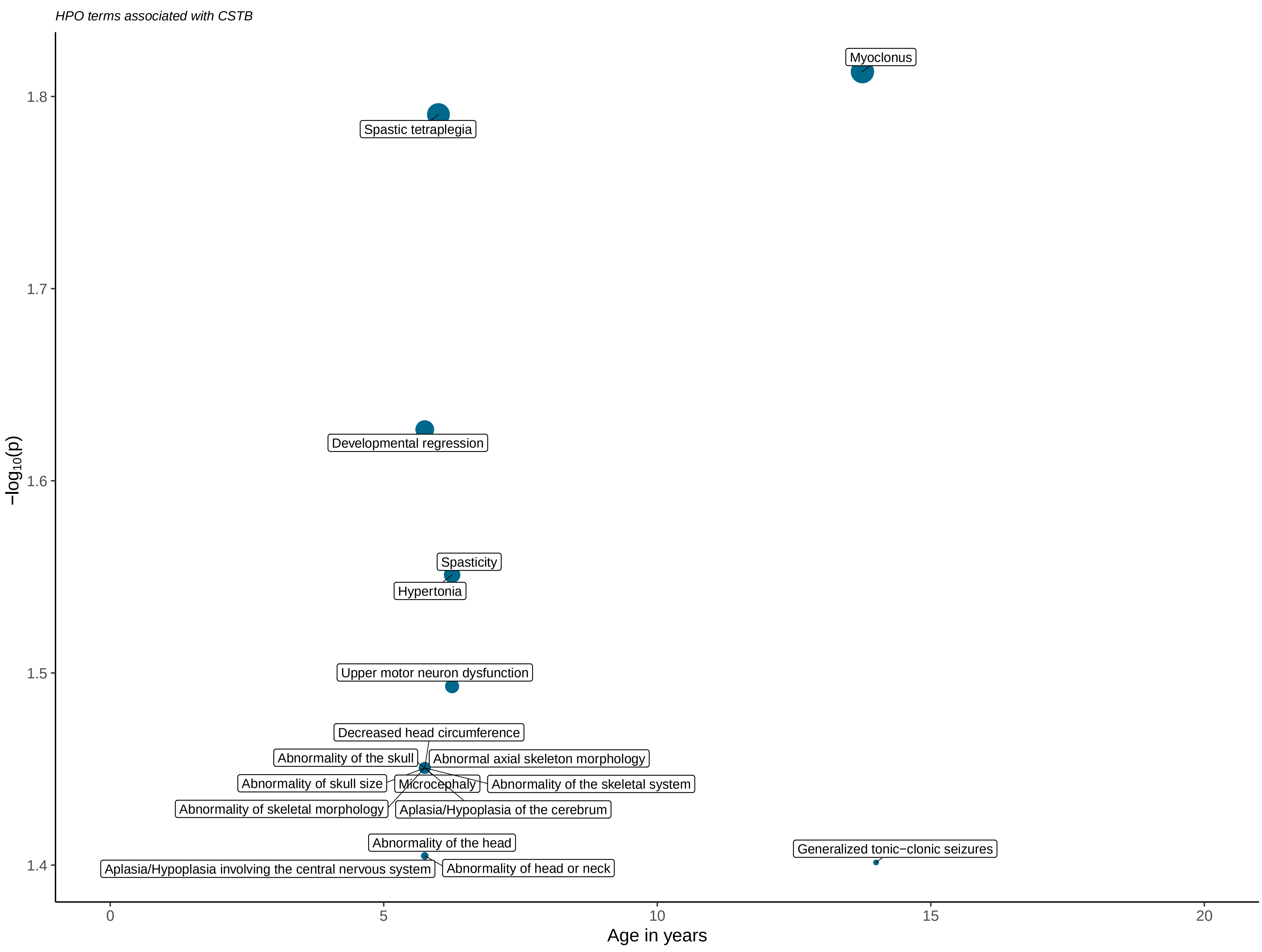
HPO terms associated with CSTB
Myoclonus
Spastic tetraplegia
Developmental regression
Spasticity
Hypertonia
Upper motor neuron dysfunction
Decreased head circumference
Abnormality of the skull
Abnormal axial skeleton morphology
Abnormality of skull size
Microcephaly
Abnormality of the skeletal system
Abnormality of skeletal morphology
Aplasia/Hypoplasia of the cerebrum
Abnormality of the head
Generalized tonic−clonic seizures
Aplasia/Hypoplasia involving the central nervous system
Abnormality of head or neck
−log10(p)
1.8
1.7
1.6
1.5
1.4
0
5
10
15
20
Age in years

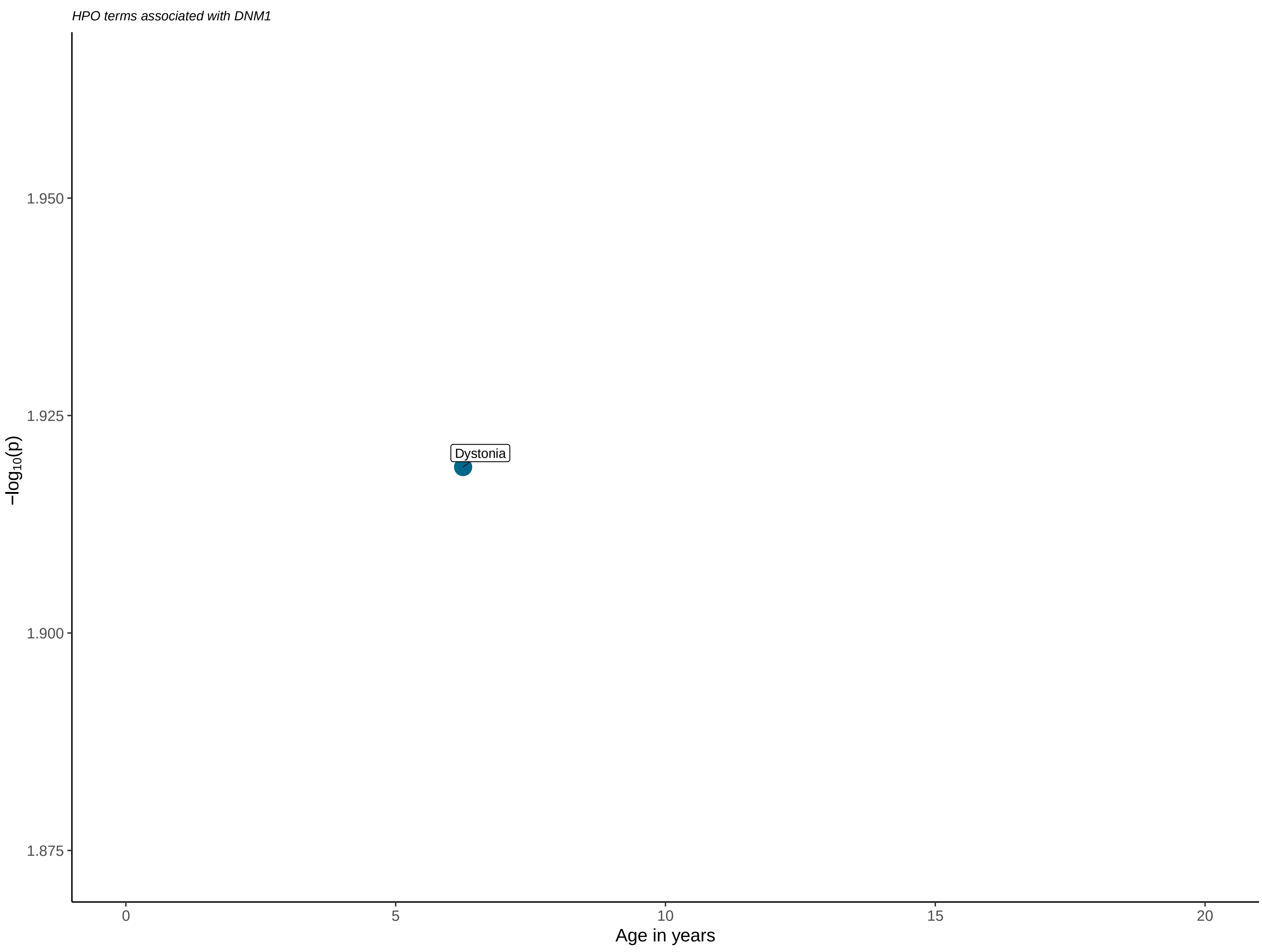

HPO terms associated with DNM1
−log10(p)
1.950
1.925
1.900
1.875
Dystonia
0
5
10
15
20
Age in years

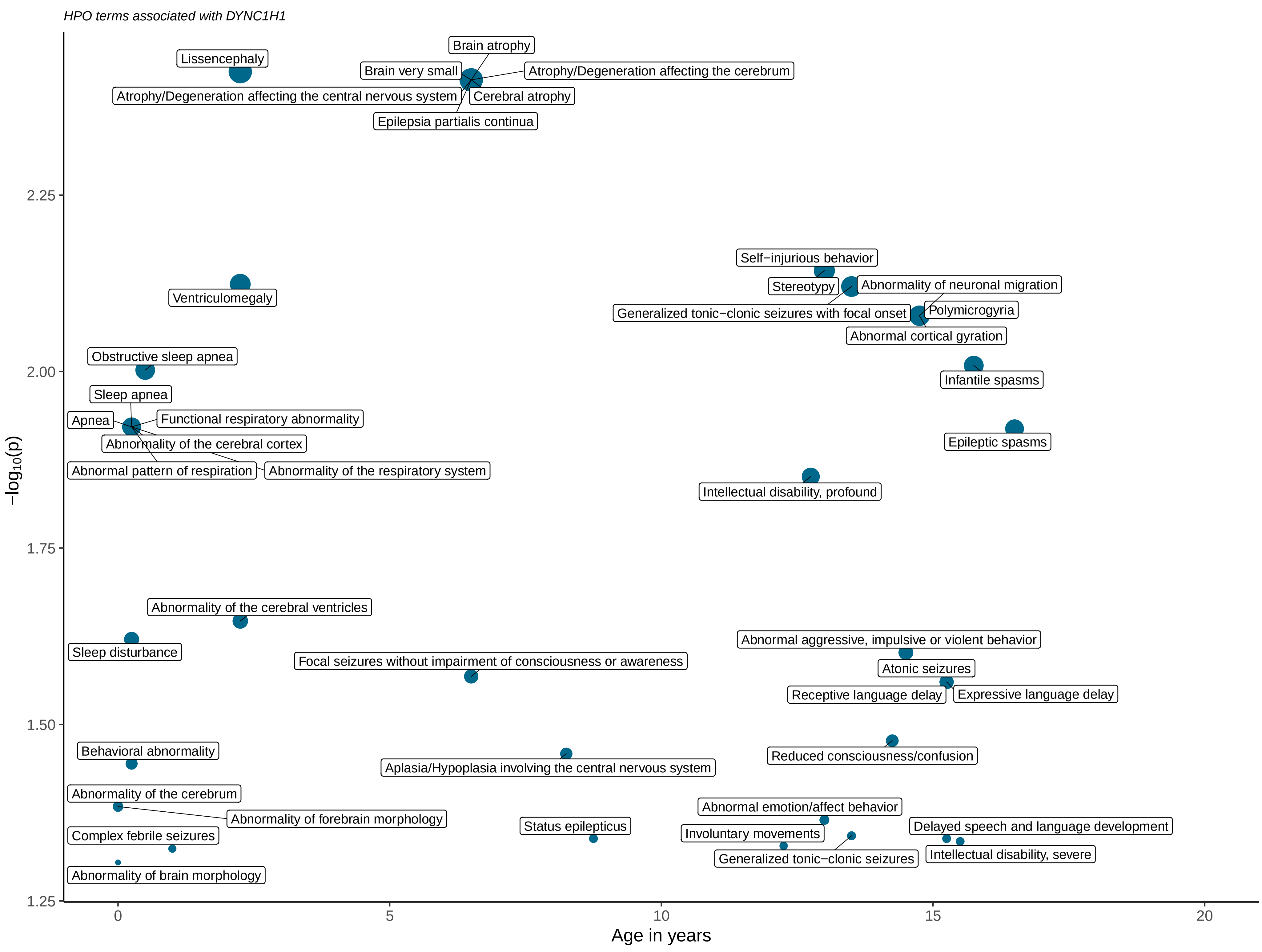
HPO terms associated with DYNC1H1
Lissencephaly
Brain atrophy
Brain very small
Atrophy/Degeneration affecting the cerebrum
Atrophy/Degeneration affecting the central nervous system
Cerebral atrophy
Epilepsia partialis continua
Ventriculomegaly
Self−injurious behavior
Stereotypy
Abnormality of neuronal migration
Generalized tonic−clonic seizures with focal onset
Polymicrogyria
Abnormal cortical gyration
Obstructive sleep apnea
Infantile spasms
Sleep apnea
Apnea
Functional respiratory abnormality
Abnormality of the cerebral cortex
Epileptic spasms
Abnormal pattern of respiration
Abnormality of the respiratory system
Intellectual disability, profound
Abnormality of the cerebral ventricles
Abnormal aggressive, impulsive or violent behavior
Sleep disturbance
Focal seizures without impairment of consciousness or awareness
Atonic seizures
Receptive language delay
Expressive language delay
Behavioral abnormality
Reduced consciousness/confusion
Aplasia/Hypoplasia involving the central nervous system
Abnormality of the cerebrum
Abnormal emotion/affect behavior
Abnormality of forebrain morphology
Status epilepticus
Delayed speech and language development
Involuntary movements
Complex febrile seizures
Generalized tonic−clonic seizures
Intellectual disability, severe
Abnormality of brain morphology
−log10(p)
2.25
2.00
1.75
1.50
1.25
0
5
10
15
20
Age in years

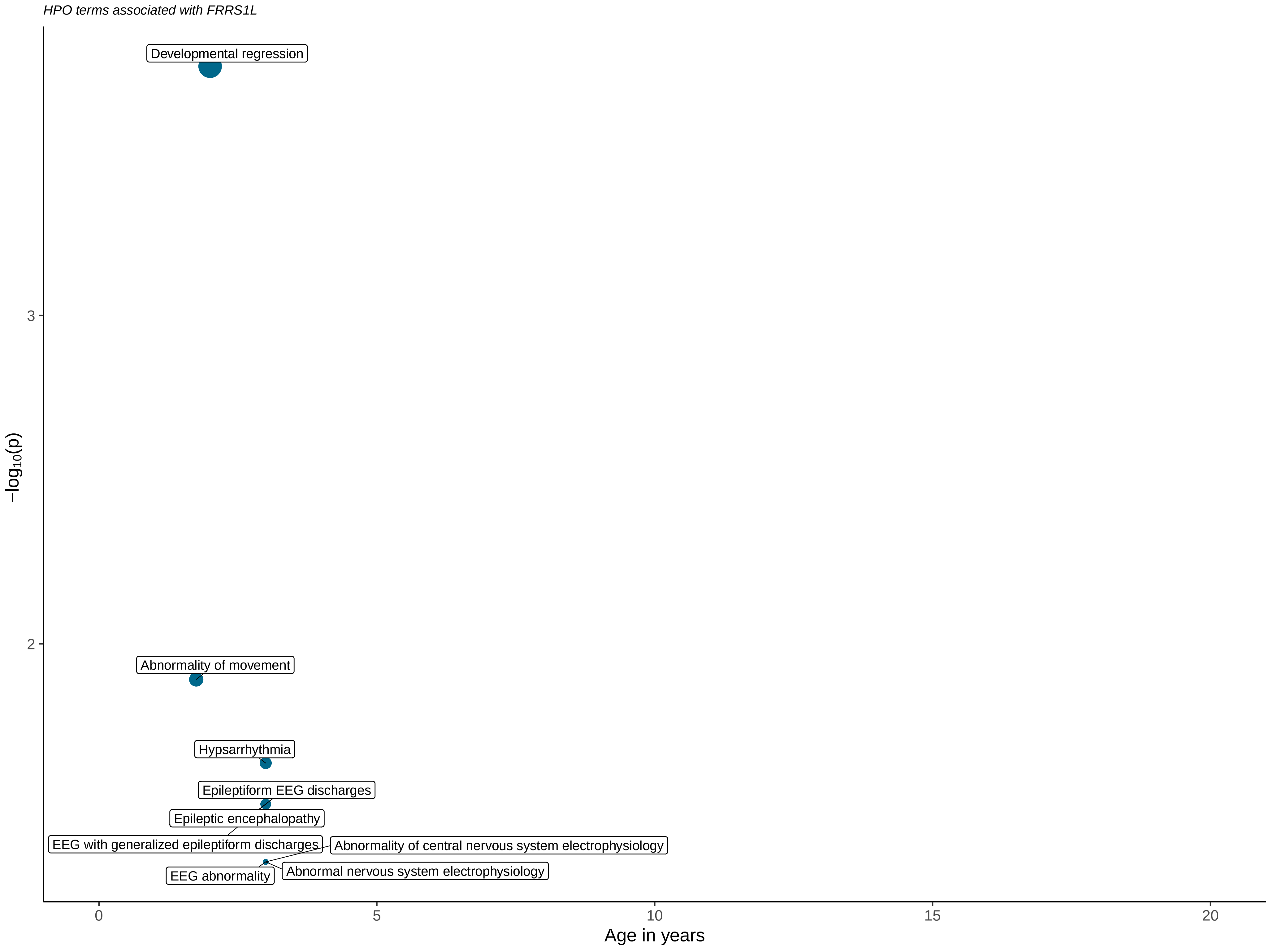
HPO terms associated with FRRS1L
Developmental regression
Abnormality of movement
Hypsarrhythmia
Epileptiform EEG discharges
Epileptic encephalopathy
EEG with generalized epileptiform discharges
Abnormality of central nervous system electrophysiology
Abnormal nervous system electrophysiology
EEG abnormality
−log10(p)
3
2
0
5
10
15
20
Age in years

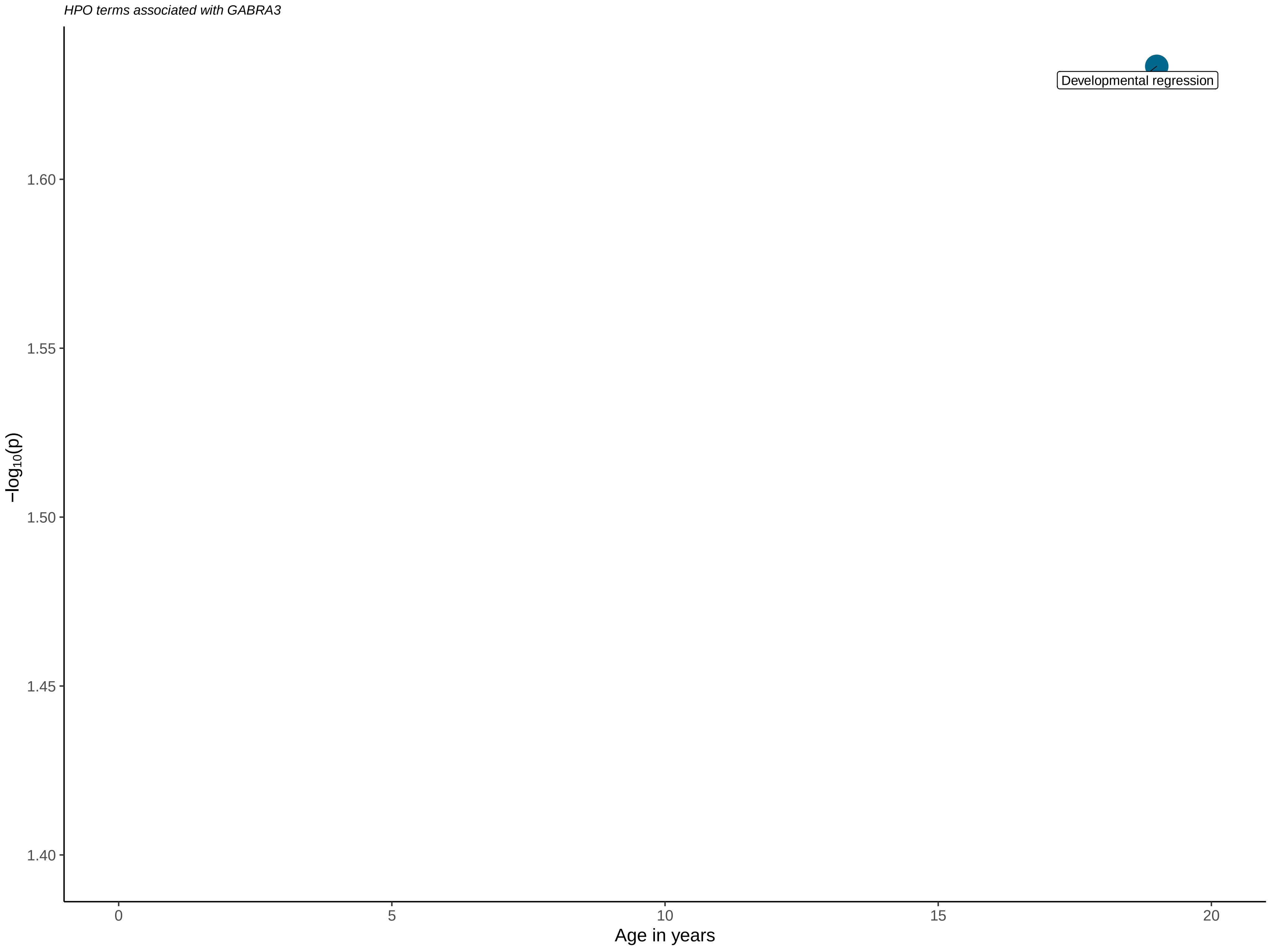
HPO terms associated with GABRA3
Developmental regression
−log10(p)
1.60
1.55
1.50
1.45
1.40
0
5
10
15
20
Age in years

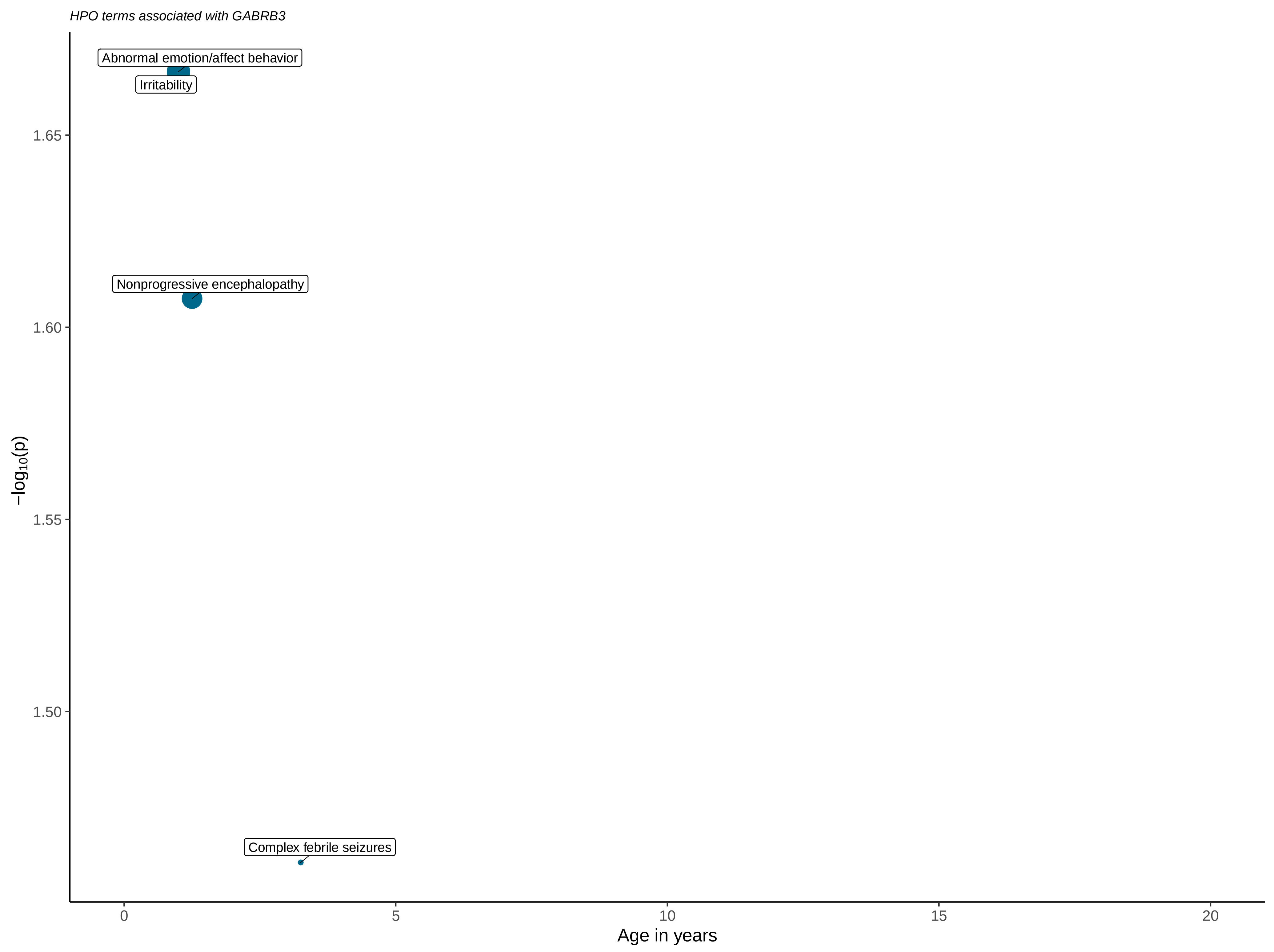

HPO terms associated with GABRB3
Abnormal emotion/affect behavior
Irritability
Nonprogressive encephalopathy
Complex febrile seizures
−log10(p)
1.65
1.60
1.55
1.50
0
5
10
15
20
Age in years

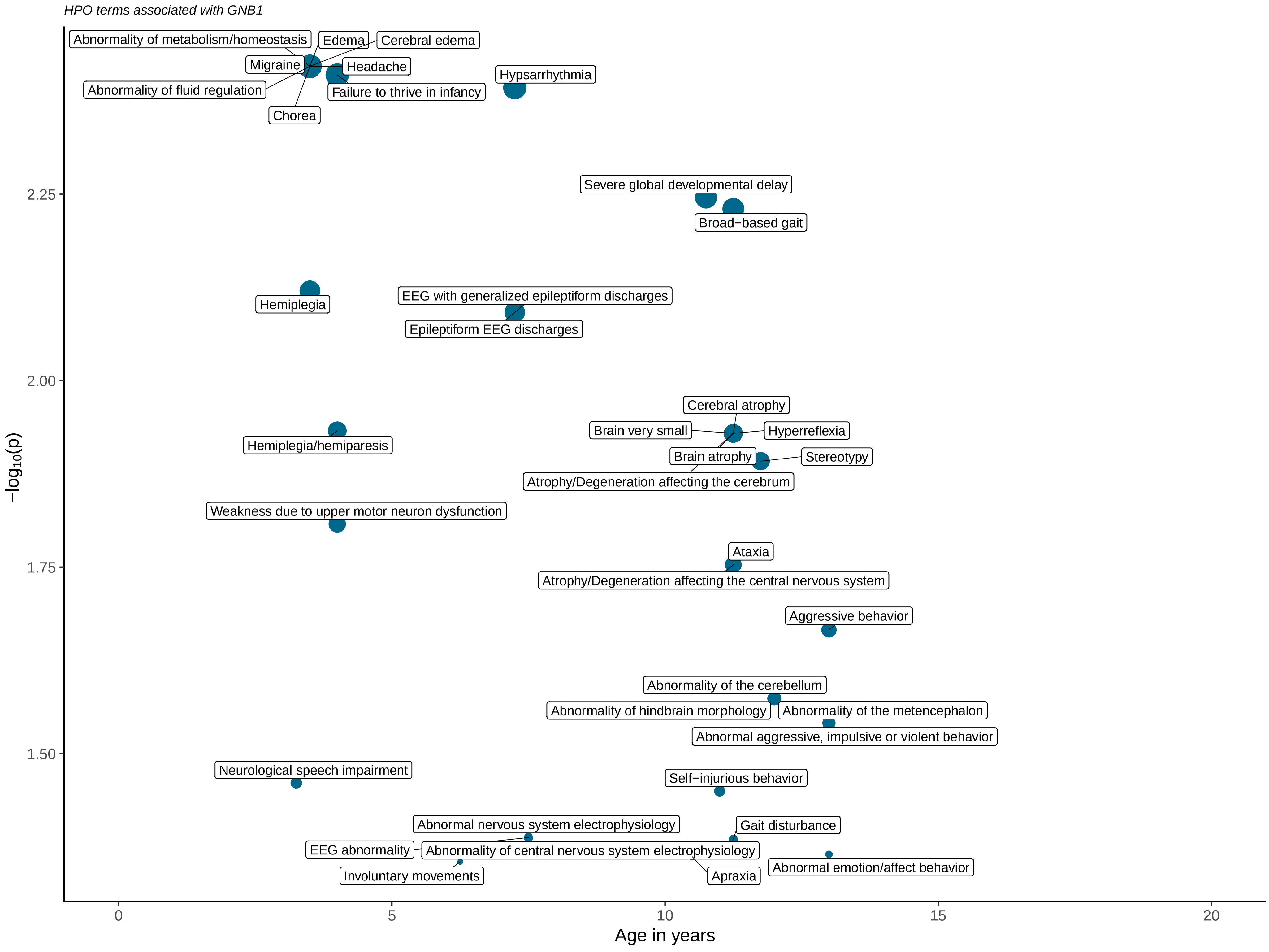
HPO terms associated with GNB1
Abnormality of metabolism/homeostasis
Edema
Cerebral edema
Migraine
Headache
Abnormality of fluid regulation
Failure to thrive in infancy
Chorea
Hypsarrhythmia
Severe global developmental delay
Broad−based gait
Hemiplegia
EEG with generalized epileptiform discharges
Epileptiform EEG discharges
Cerebral atrophy
Brain very small
Hyperreflexia
Hemiplegia/hemiparesis
Brain atrophy
Stereotypy
Atrophy/Degeneration affecting the cerebrum
Weakness due to upper motor neuron dysfunction
Ataxia
Atrophy/Degeneration affecting the central nervous system
Aggressive behavior
Abnormality of the cerebellum
Abnormality of hindbrain morphology
Abnormality of the metencephalon
Abnormal aggressive, impulsive or violent behavior
Neurological speech impairment
Self−injurious behavior
Abnormal nervous system electrophysiology
Gait disturbance
EEG abnormality
Abnormality of central nervous system electrophysiology
Involuntary movements
Apraxia
Abnormal emotion/affect behavior
−log10(p)
2.25
2.00
1.75
1.50
0
5
10
15
20
Age in years

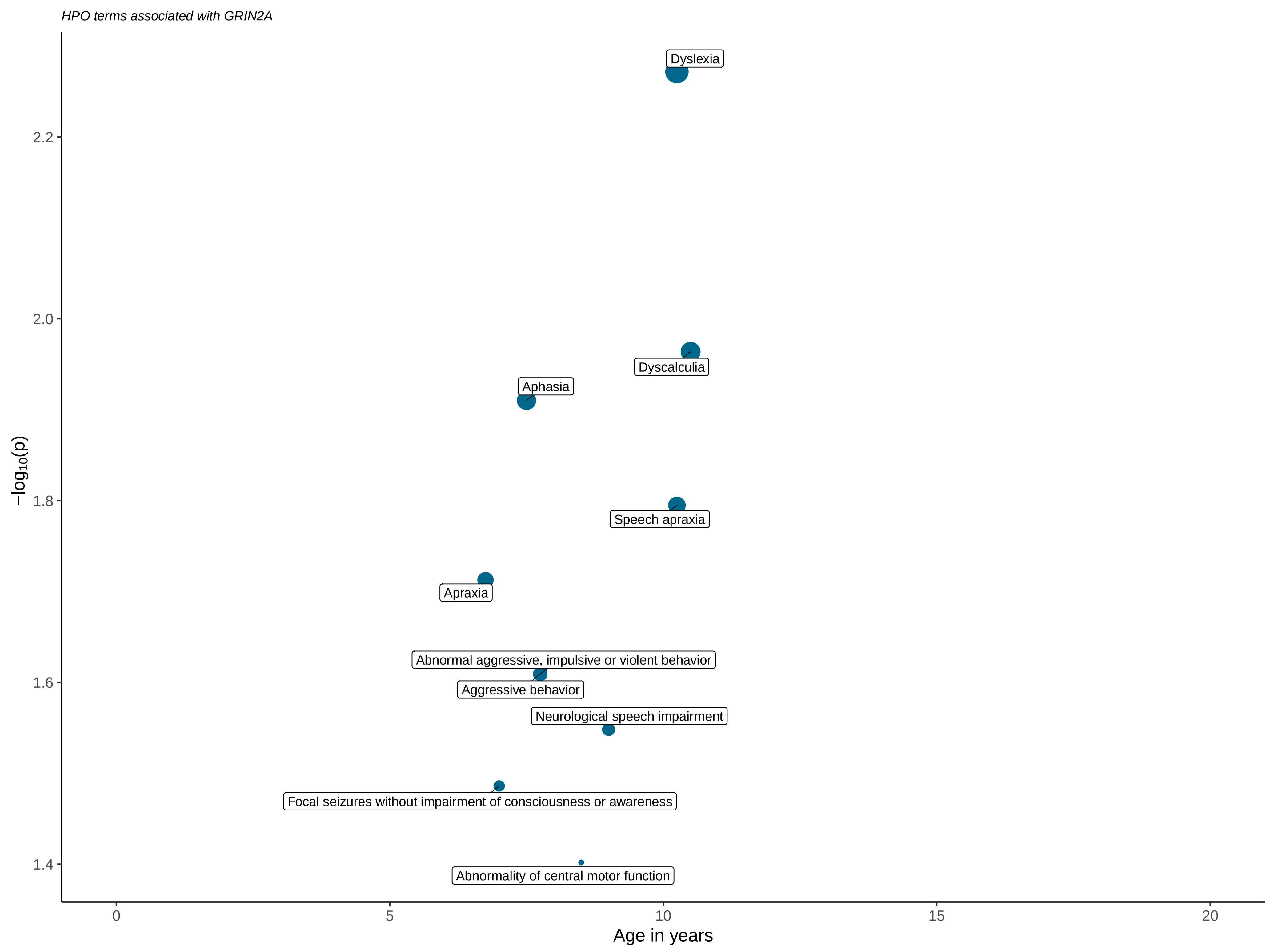

HPO terms associated with GRIN2A
Dyslexia
Dyscalculia
Aphasia
Speech apraxia
Apraxia
Abnormal aggressive, impulsive or violent behavior
Aggressive behavior
Neurological speech impairment
Focal seizures without impairment of consciousness or awareness
Abnormality of central motor function
−log10(p)
2.2
2.0
1.8
1.6
1.4
0
5
10
15
20
Age in years

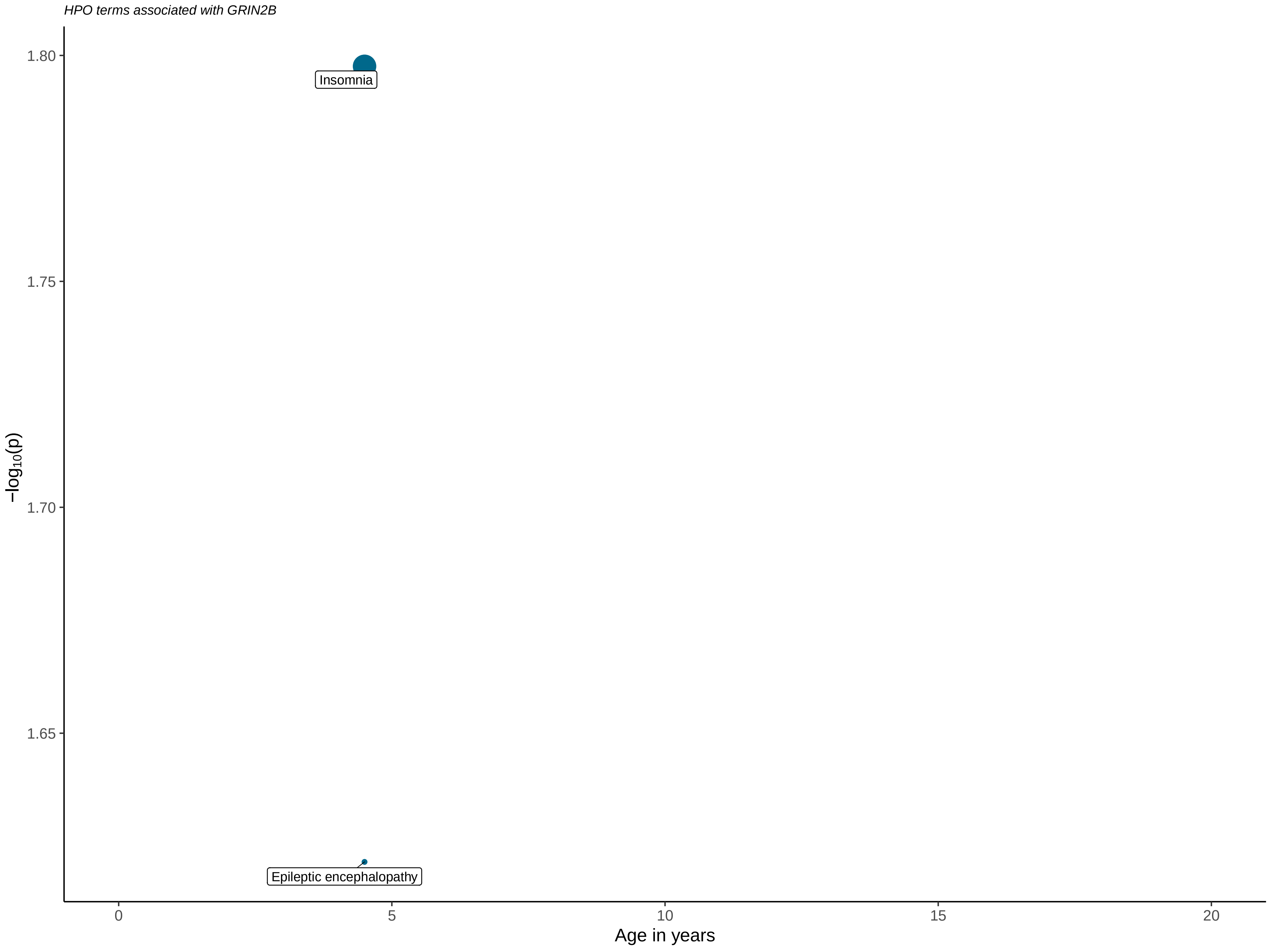
HPO terms associated with GRIN2B
1.80
1.75
1.70
1.65
−log10(p)
Insomnia
Epileptic encephalopathy
0
5
10
15
20
Age in years

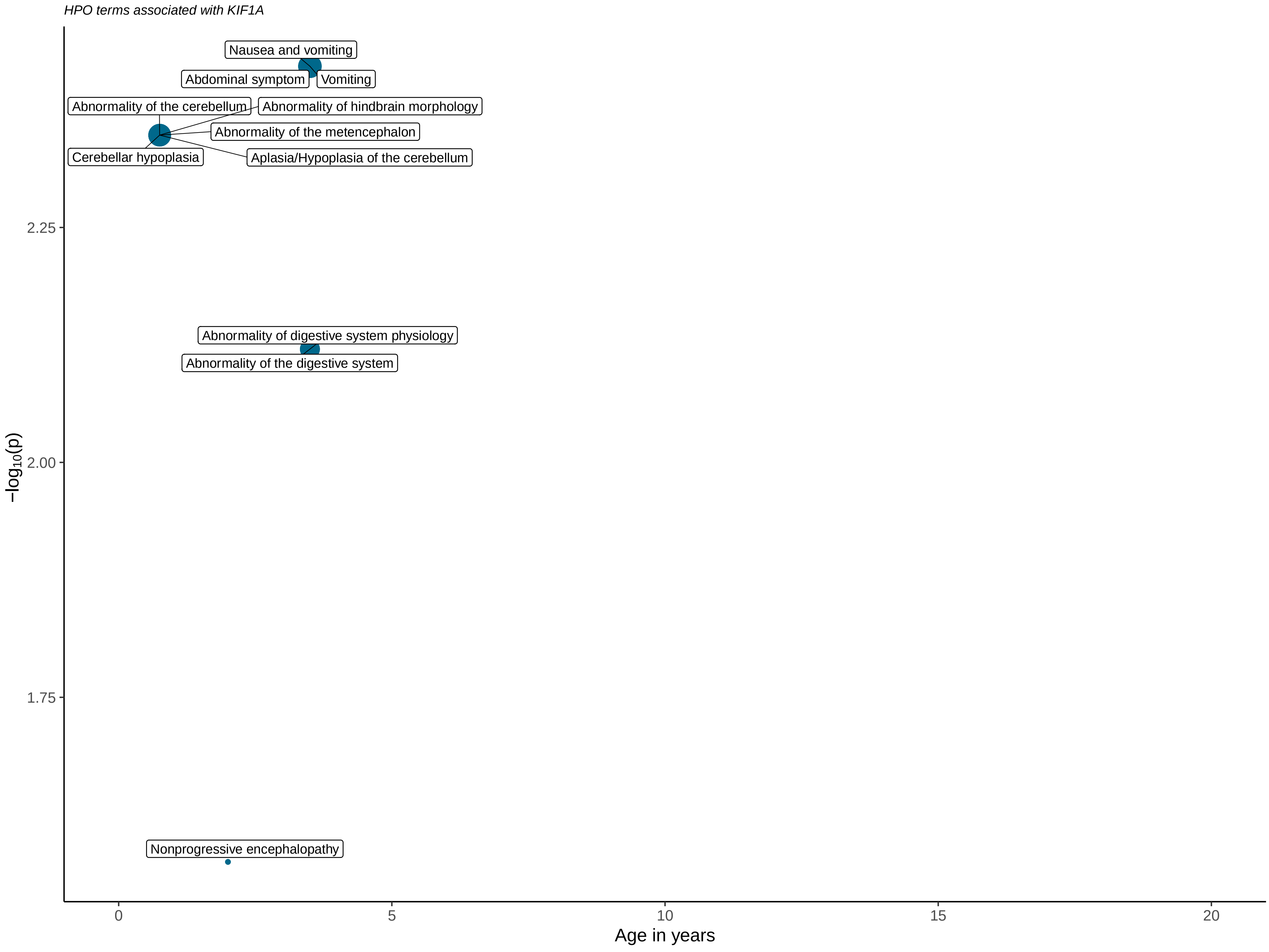

HPO terms associated with KIF1A
Nausea and vomiting
Abdominal symptom
Vomiting
Abnormality of the cerebellum
Abnormality of hindbrain morphology
Abnormality of the metencephalon
Cerebellar hypoplasia
Aplasia/Hypoplasia of the cerebellum
Abnormality of digestive system physiology
Abnormality of the digestive system
Nonprogressive encephalopathy
2.25
2.00
1.75
$-\log_{10}(p)$
0
5
10
15
20
Age in years

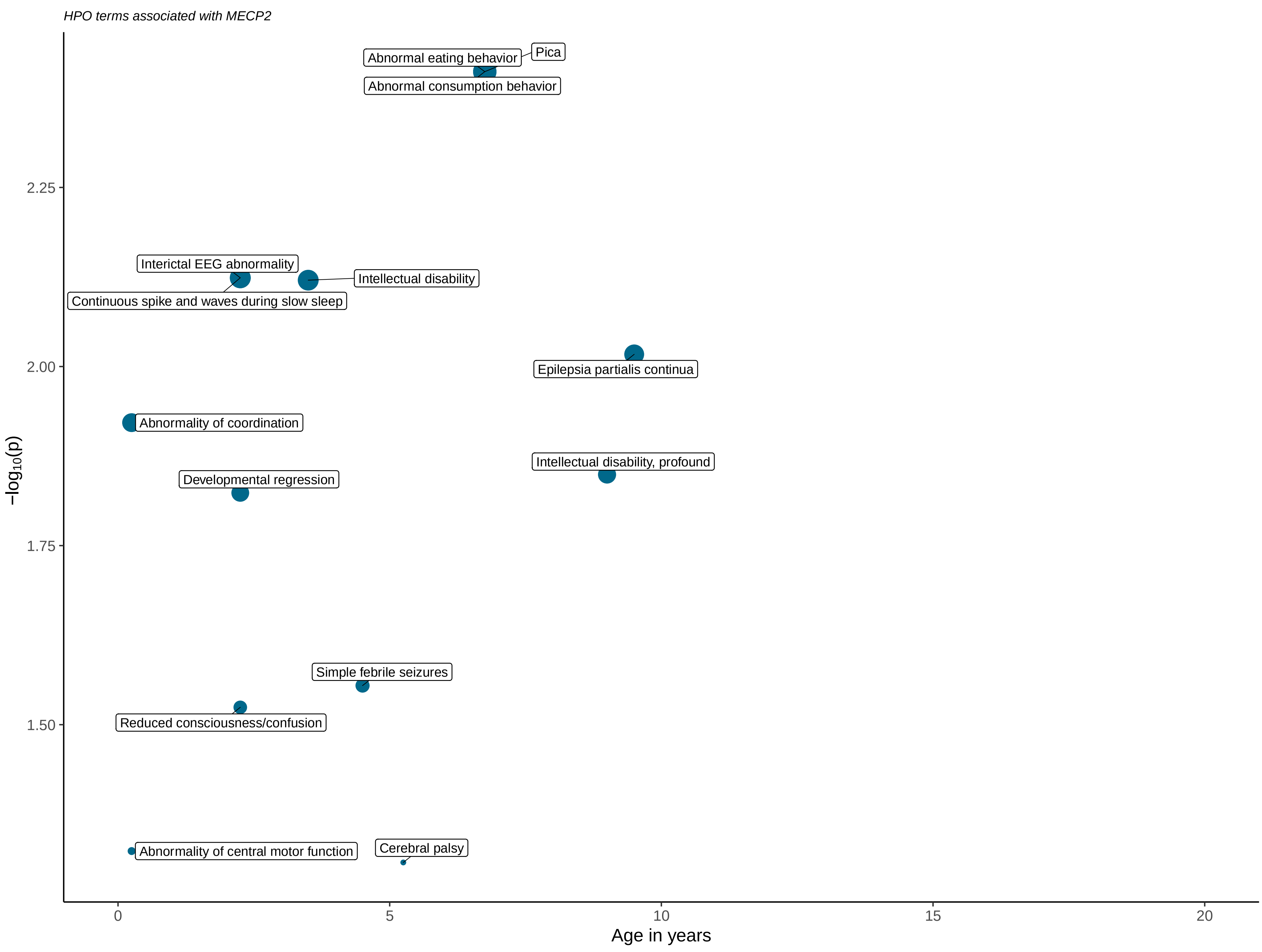

HPO terms associated with MECP2
Abnormal eating behavior
Pica
Abnormal consumption behavior
Interictal EEG abnormality
Intellectual disability
Continuous spike and waves during slow sleep
Epilepsia partialis continua
Abnormality of coordination
Intellectual disability, profound
Developmental regression
Simple febrile seizures
Reduced consciousness/confusion
Abnormality of central motor function
Cerebral palsy
2.25
2.00
1.75
1.50
−log10(p)
0
5
10
15
20
Age in years

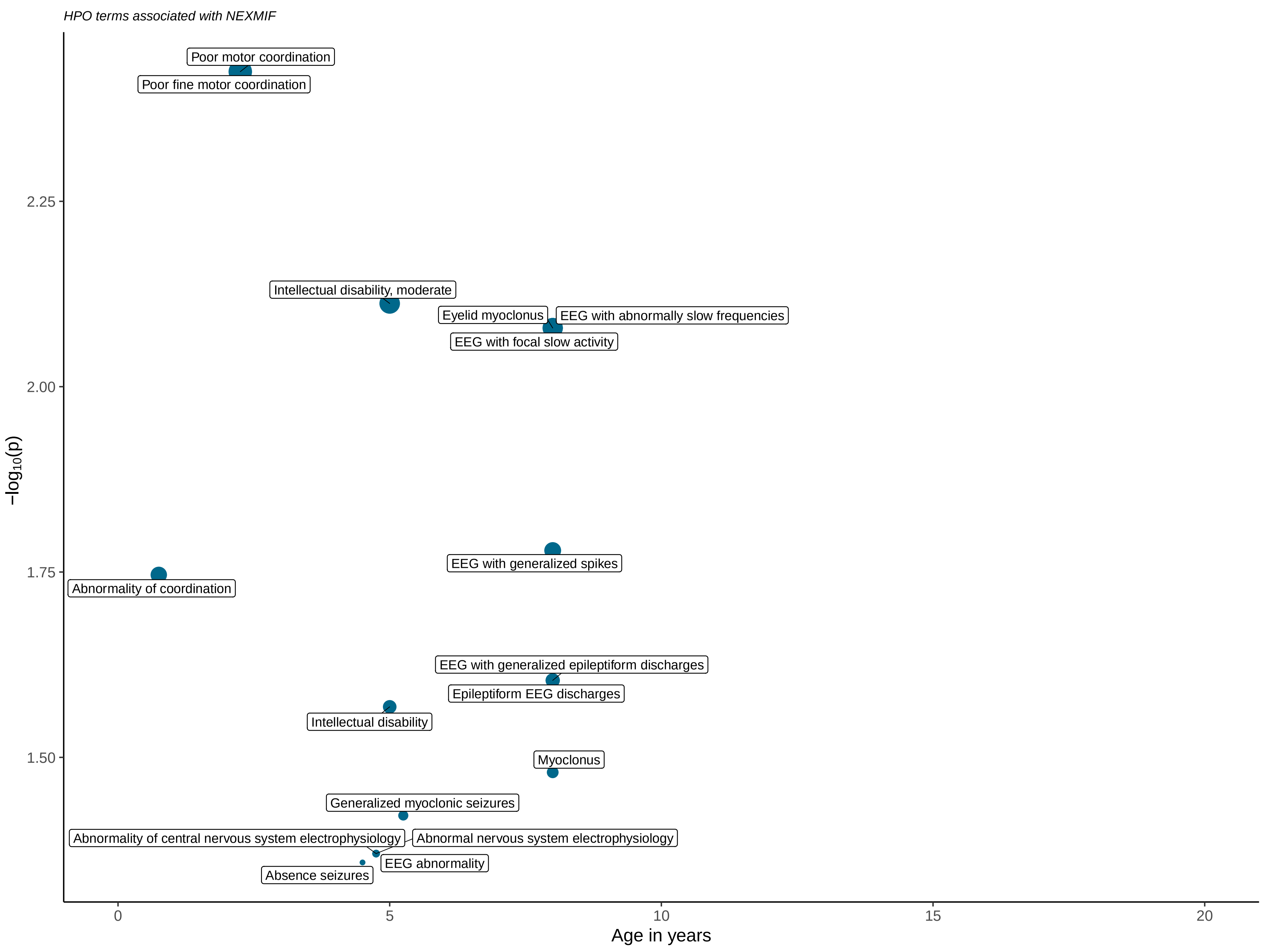

HPO terms associated with NEXMIF
Poor motor coordination
Poor fine motor coordination
Intellectual disability, moderate
Eyelid myoclonus
EEG with abnormally slow frequencies
EEG with focal slow activity
EEG with generalized spikes
Abnormality of coordination
EEG with generalized epileptiform discharges
Epileptiform EEG discharges
Intellectual disability
Myoclonus
Generalized myoclonic seizures
Abnormality of central nervous system electrophysiology
Abnormal nervous system electrophysiology
EEG abnormality
Absence seizures
−log10(p)
2.25
2.00
1.75
1.50
0
5
10
15
20
Age in years

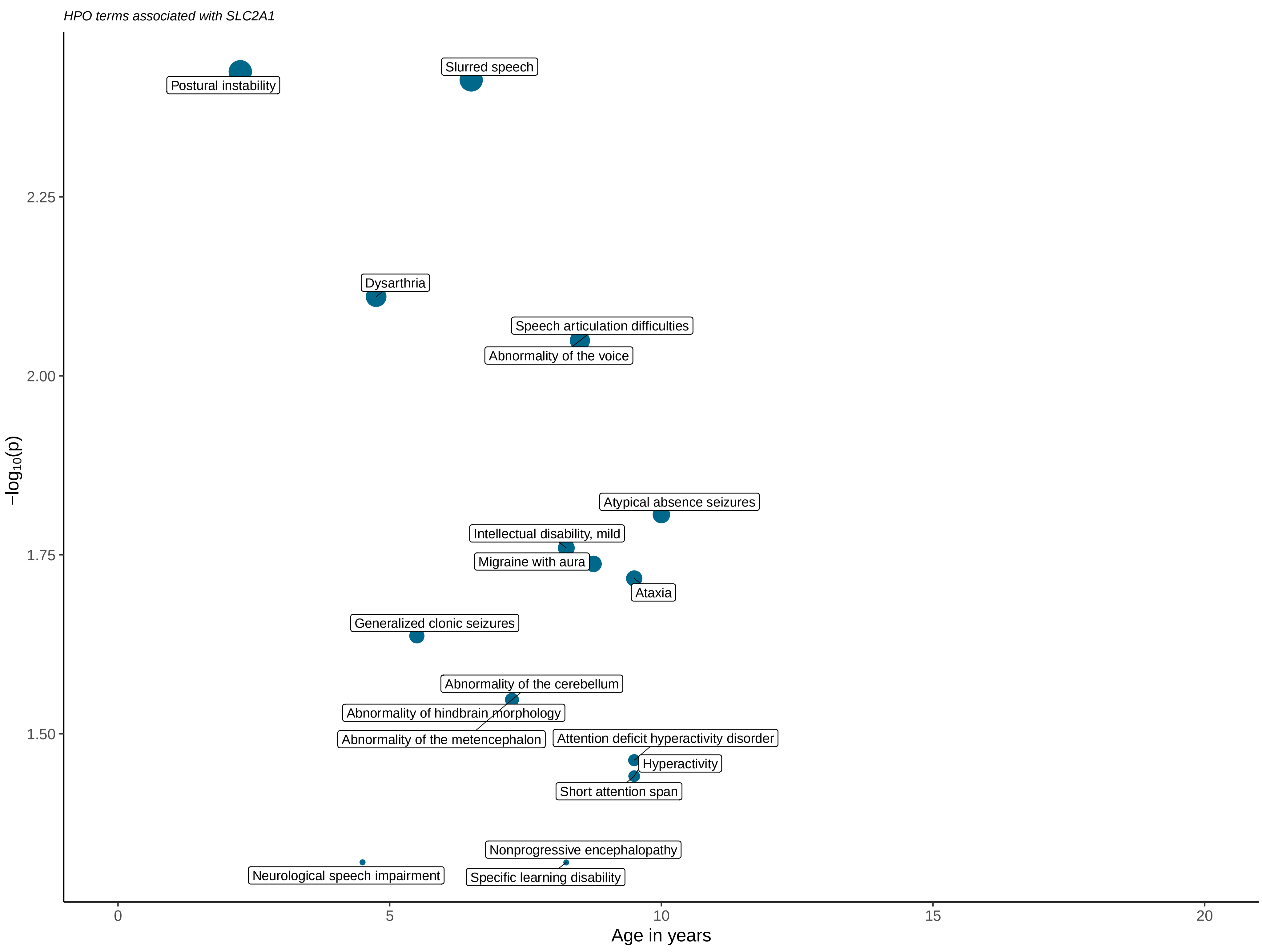

HPO terms associated with SLC2A1
Postural instability
Slurred speech
Dysarthria
Speech articulation difficulties
Abnormality of the voice
Atypical absence seizures
Intellectual disability, mild
Migraine with aura
Ataxia
Generalized clonic seizures
Abnormality of the cerebellum
Abnormality of hindbrain morphology
Abnormality of the metencephalon
Attention deficit hyperactivity disorder
Hyperactivity
Short attention span
Nonprogressive encephalopathy
Neurological speech impairment
Specific learning disability
2.25
2.00
1.75
1.50
−log10(p)
0
5
10
15
20
Age in years

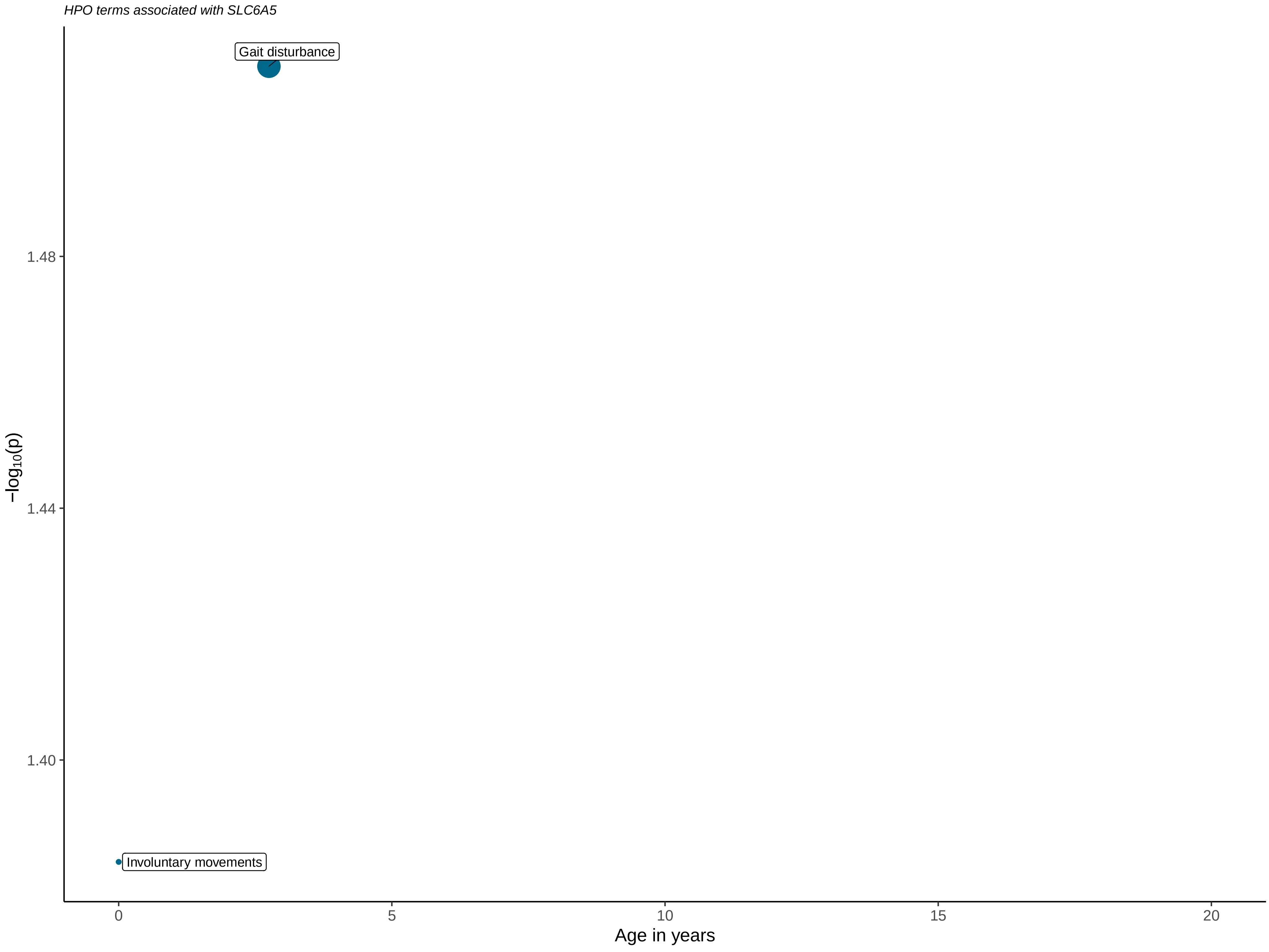
HPO terms associated with SLC6A5
Gait disturbance
Involuntary movements
1.48
1.44
1.40
−log10(p)
0
5
10
15
20
Age in years

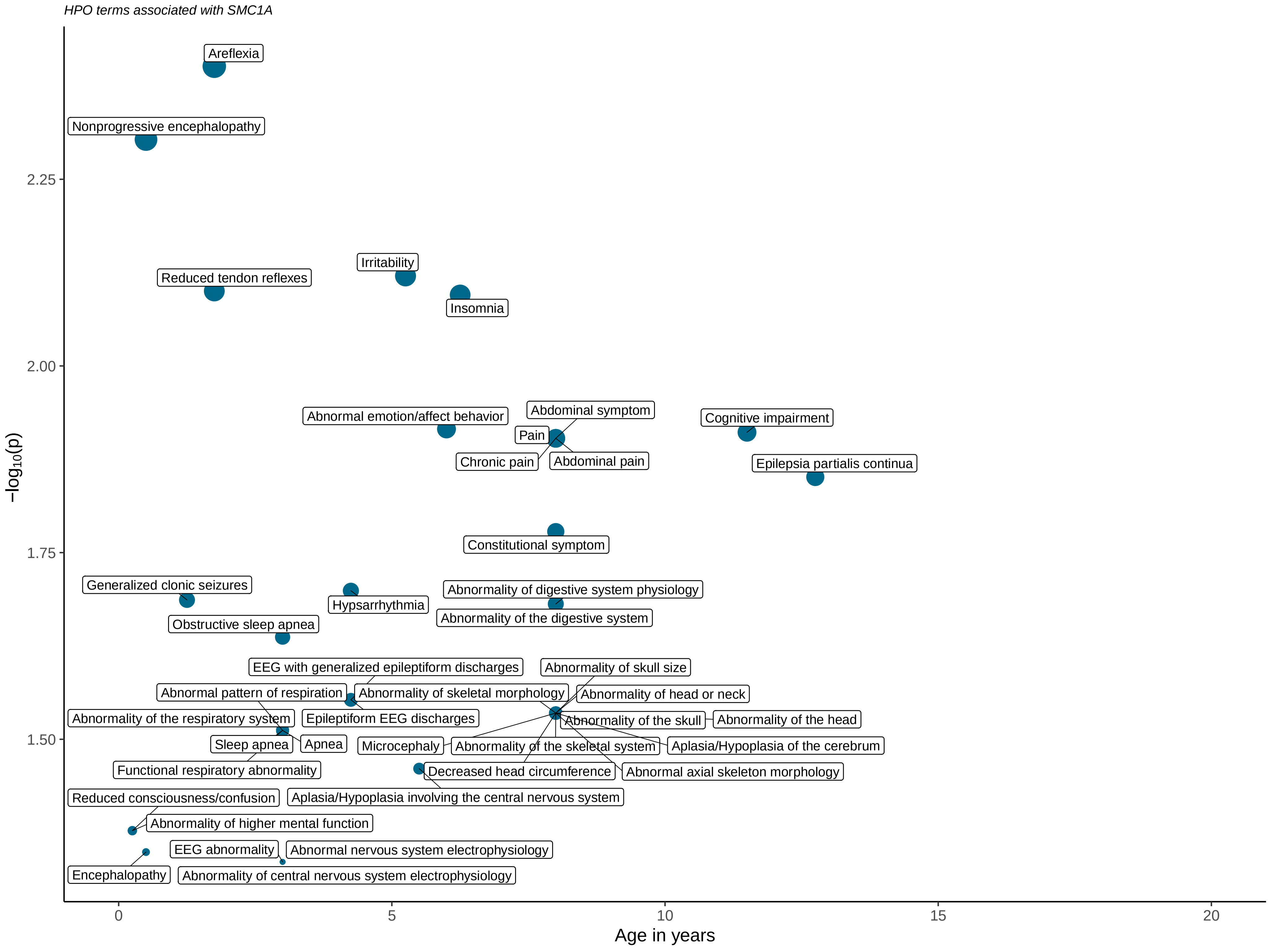

HPO terms associated with SMC1A
Areflexia
Nonprogressive encephalopathy
Reduced tendon reflexes
Irritability
Insomnia
Abnormal emotion/affect behavior
Abdominal symptom
Pain
Chronic pain
Abdominal pain
Cognitive impairment
Epilepsia partialis continua
Constitutional symptom
Generalized clonic seizures
Hypsarrhythmia
Abnormality of digestive system physiology
Abnormality of the digestive system
Obstructive sleep apnea
EEG with generalized epileptiform discharges
Abnormality of skull size
Abnormal pattern of respiration
Abnormality of skeletal morphology
Abnormality of head or neck
Abnormality of the respiratory system
Epileptiform EEG discharges
Abnormality of the skull
Abnormality of the head
Sleep apnea
Apnea
Microcephaly
Abnormality of the skeletal system
Aplasia/Hypoplasia of the cerebrum
Functional respiratory abnormality
Decreased head circumference
Abnormal axial skeleton morphology
Reduced consciousness/confusion
Aplasia/Hypoplasia involving the central nervous system
Abnormality of higher mental function
EEG abnormality
Abnormal nervous system electrophysiology
Encephalopathy
Abnormality of central nervous system electrophysiology
−log10(p)
2.25
2.00
1.75
1.50
0
5
10
15
20
Age in years

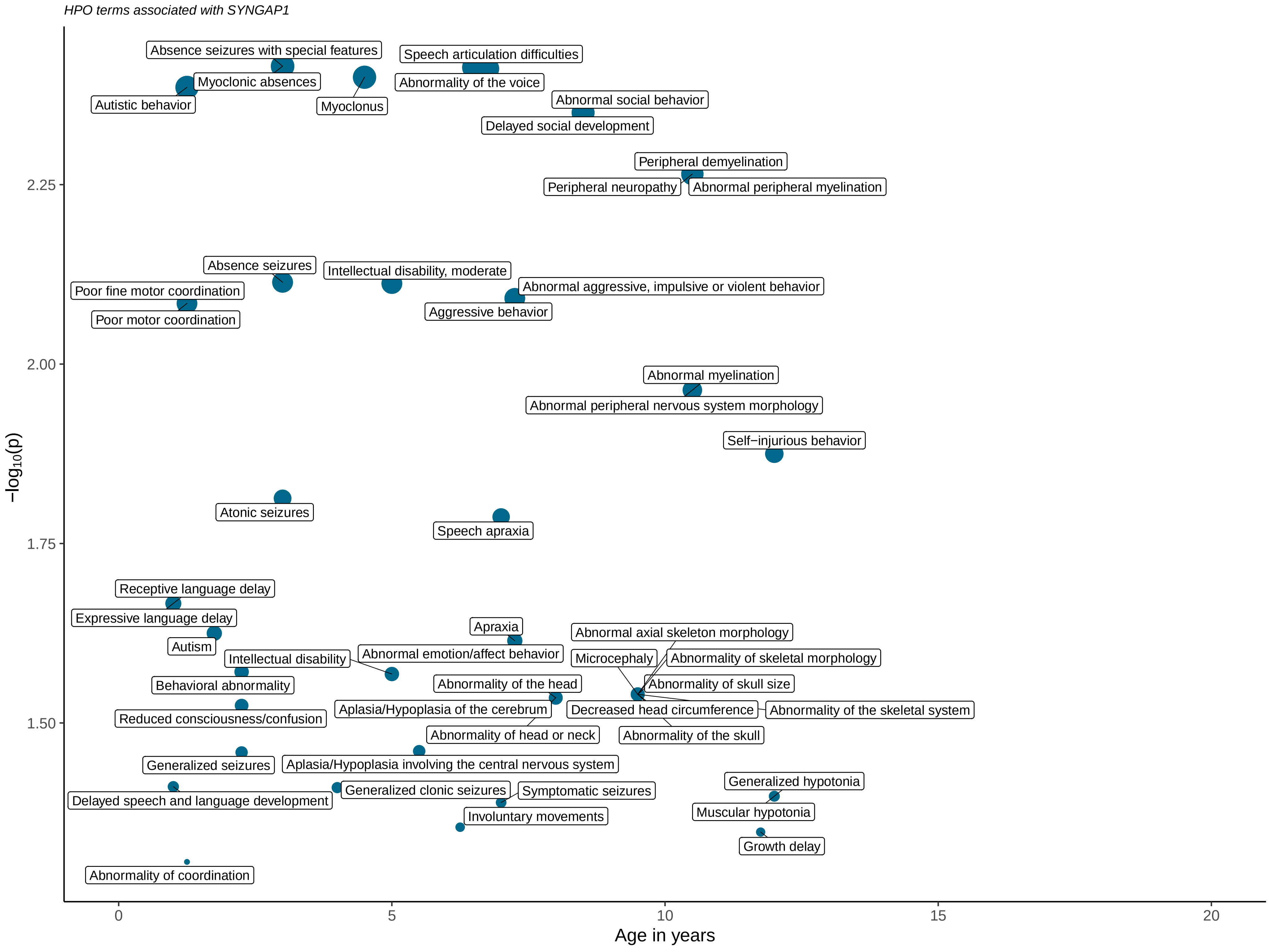
HPO terms associated with SYNGAP1
Absence seizures with special features
Myoclonic absences
Autistic behavior
Myoclonus
Speech articulation difficulties
Abnormality of the voice
Abnormal social behavior
Delayed social development
Peripheral demyelination
Peripheral neuropathy
Abnormal peripheral myelination
Absence seizures
Poor fine motor coordination
Poor motor coordination
Intellectual disability, moderate
Abnormal aggressive, impulsive or violent behavior
Aggressive behavior
Abnormal myelination
Abnormal peripheral nervous system morphology
Self−injurious behavior
Atonic seizures
Speech apraxia
Receptive language delay
Expressive language delay
Autism
Apraxia
Abnormal axial skeleton morphology
Intellectual disability
Abnormal emotion/affect behavior
Microcephaly
Abnormality of skeletal morphology
Behavioral abnormality
Abnormality of the head
Abnormality of skull size
Reduced consciousness/confusion
Aplasia/Hypoplasia of the cerebrum
Decreased head circumference
Abnormality of the skeletal system
Abnormality of head or neck
Abnormality of the skull
Generalized seizures
Aplasia/Hypoplasia involving the central nervous system
Generalized hypotonia
Delayed speech and language development
Generalized clonic seizures
Symptomatic seizures
Muscular hypotonia
Involuntary movements
Growth delay
Abnormality of coordination
−log10(p)
2.25
2.00
1.75
1.50
0
5
10
15
20
Age in years

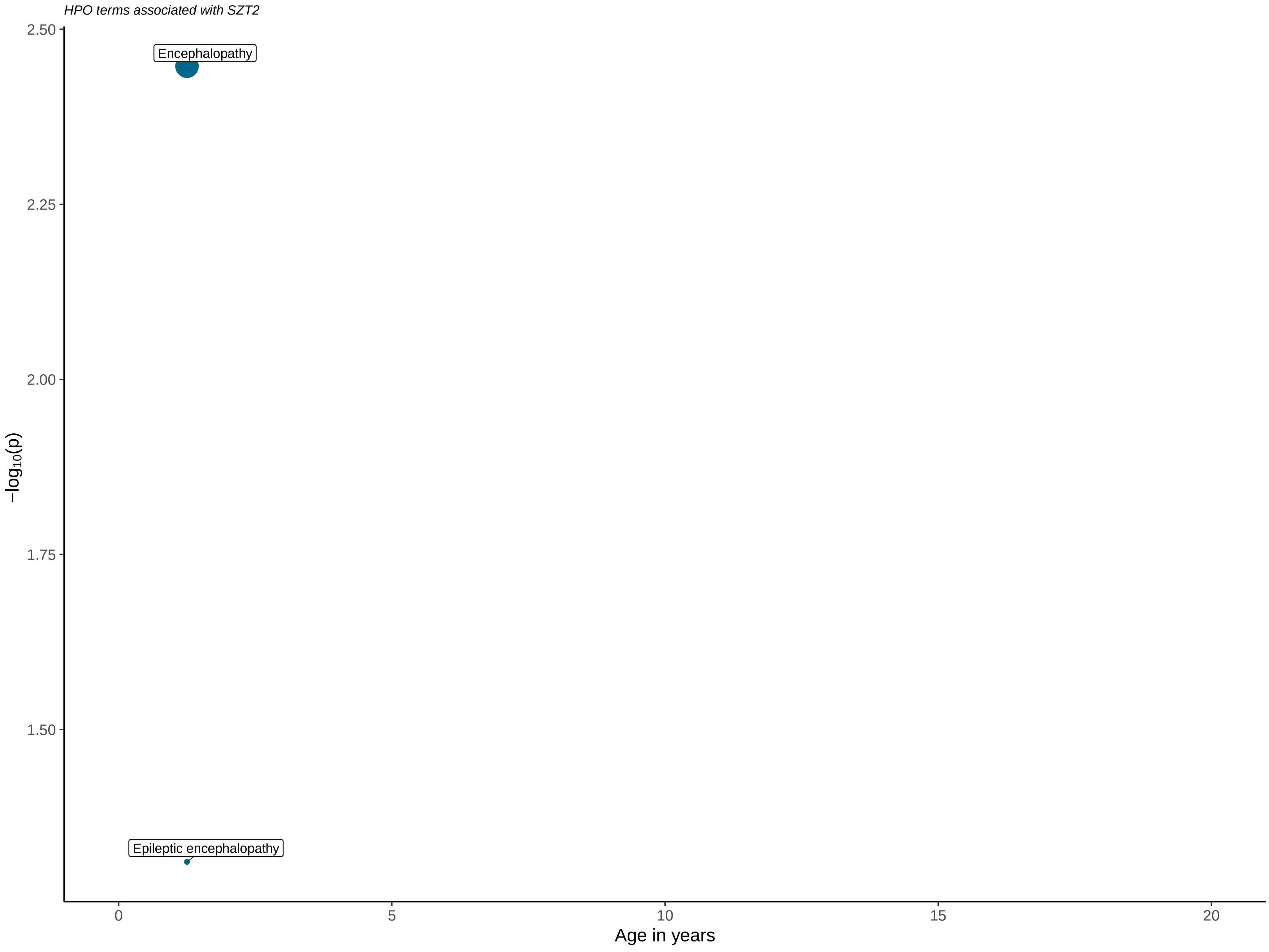
HPO terms associated with SZT2
Encephalopathy
Epileptic encephalopathy
2.50
2.25
2.00
1.75
1.50
−log10(p)
0
5
10
15
20
Age in years

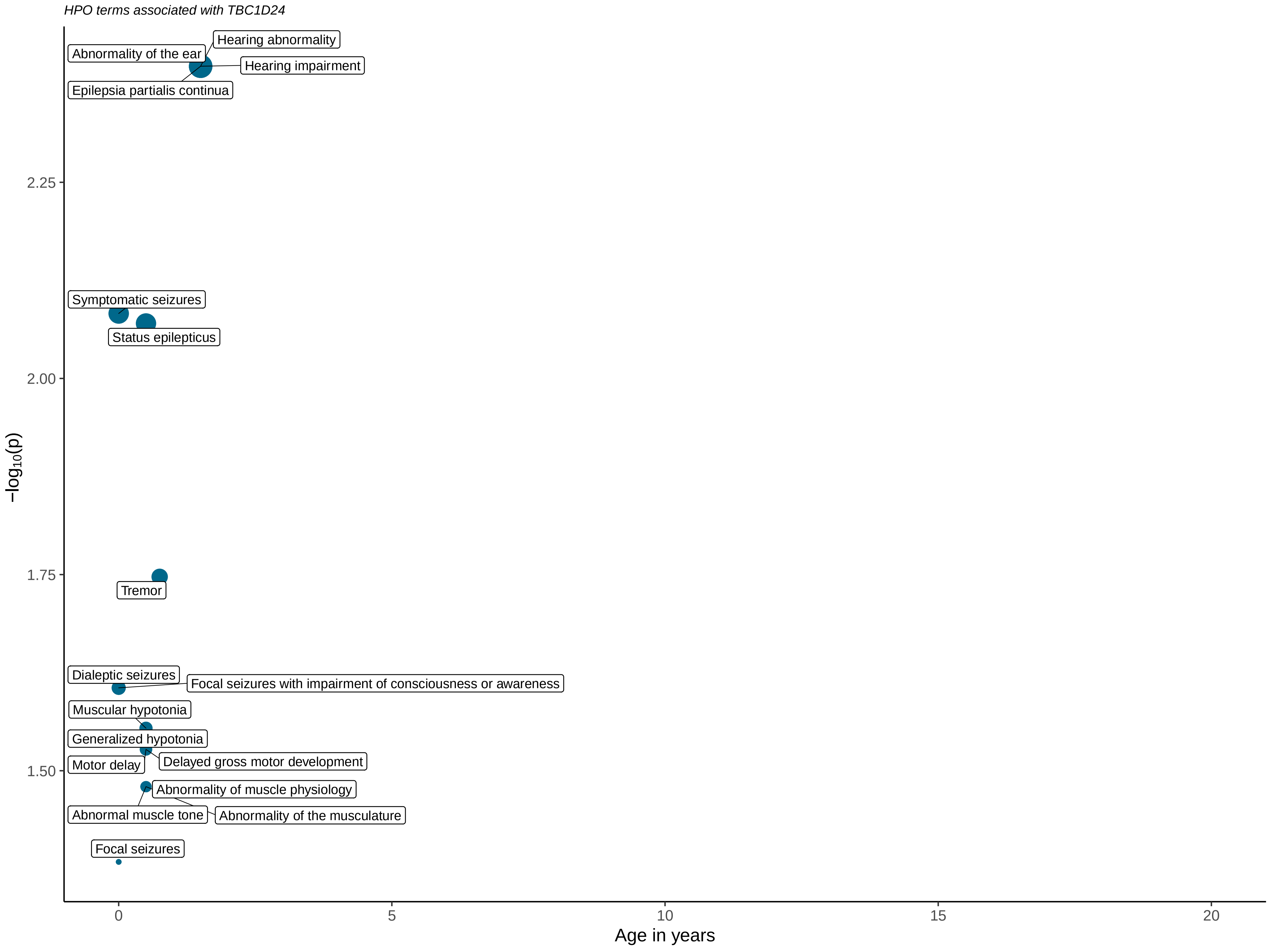

HPO terms associated with TBC1D24
Hearing abnormality
Abnormality of the ear
Hearing impairment
Epilepsia partialis continua
Symptomatic seizures
Status epilepticus
Tremor
Dialeptic seizures
Focal seizures with impairment of consciousness or awareness
Muscular hypotonia
Generalized hypotonia
Delayed gross motor development
Motor delay
Abnormality of muscle physiology
Abnormal muscle tone
Abnormality of the musculature
Focal seizures
2.25
2.00
1.75
1.50
−log10(p)
0
5
10
15
20
Age in years

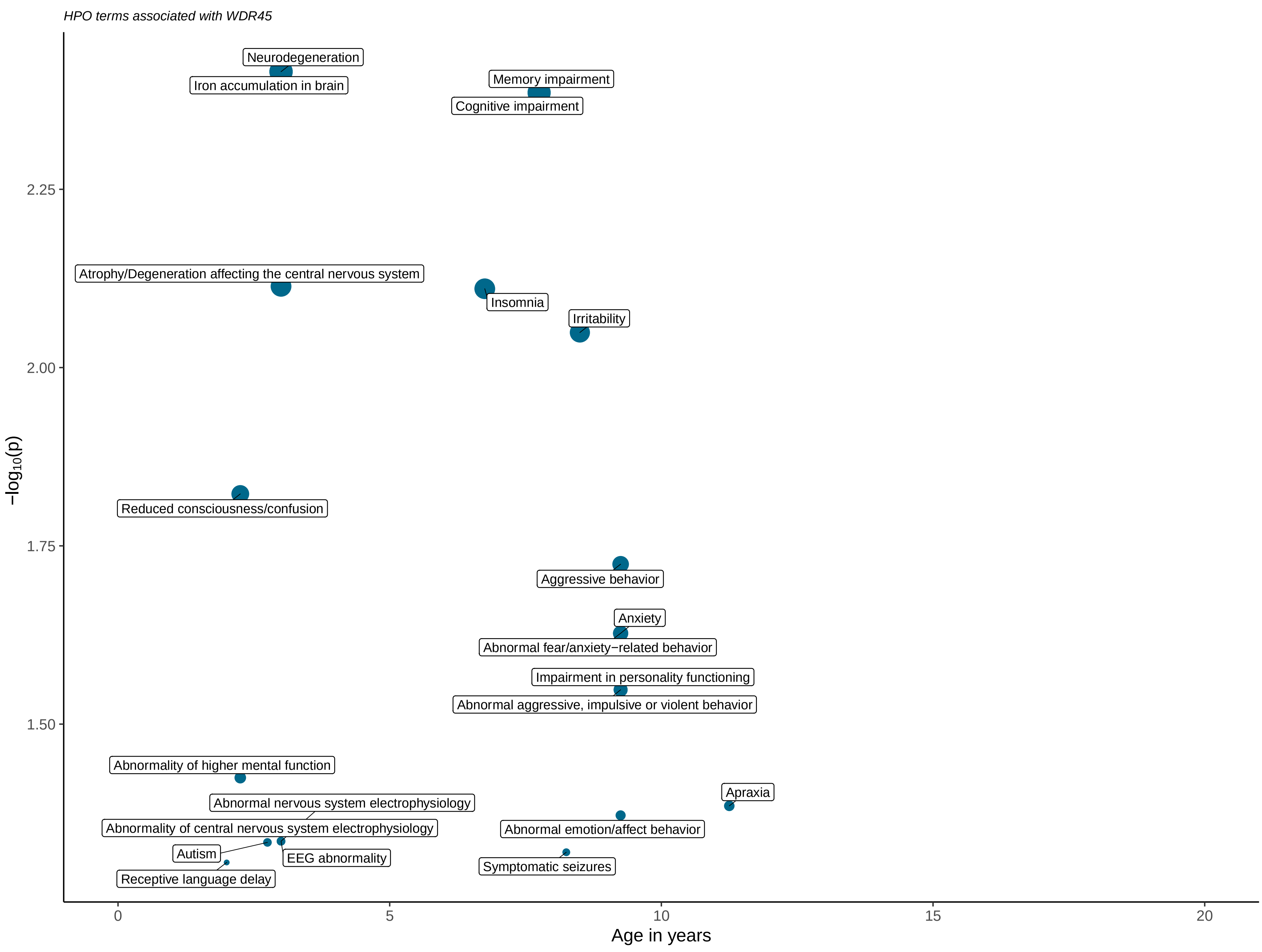

HPO terms associated with WDR45
Neurodegeneration
Iron accumulation in brain
Memory impairment
Cognitive impairment
Atrophy/Degeneration affecting the central nervous system
Insomnia
Irritability
Reduced consciousness/confusion
Aggressive behavior
Anxiety
Abnormal fear/anxiety−related behavior
Impairment in personality functioning
Abnormal aggressive, impulsive or violent behavior
Abnormality of higher mental function
Apraxia
Abnormal nervous system electrophysiology
Abnormality of central nervous system electrophysiology
Abnormal emotion/affect behavior
Autism
EEG abnormality
Symptomatic seizures
Receptive language delay
2.25
2.00
1.75
1.50
−log10(p)
0
5
10
15
20
Age in years
